# Comparative evaluation of spatiotemporal methods for effective dengue cluster detection with a case study of national surveillance data in Thailand

**DOI:** 10.1038/s41598-024-82212-1

**Published:** 2024-12-28

**Authors:** Chawarat Rotejanaprasert, Kawin Chinpong, Andrew B. Lawson, Richard J. Maude

**Affiliations:** 1 Department of Tropical Hygiene, Faculty of Tropical Medicine, Mahidol University, Bangkok, Thailand; 2 Mahidol-Oxford Tropical Medicine Research Unit, Faculty of Tropical Medicine, Mahidol University, Bangkok, Thailand; 3 Chulabhorn Learning and Research Centre, Chulabhorn Royal Academy, Bangkok, Thailand; 4 Department of Public Health Sciences, Medical University of South Carolina, Charleston, SC USA; 5 Usher Institute, University of Edinburgh, Edinburgh, UK; 6 Centre for Tropical Medicine and Global Health, Nuffield Department of Medicine, University of Oxford, Oxford, UK; 7 The Open University, Milton Keynes, UK

**Keywords:** Spatiotemporal, Cluster detection, Dengue, Surveillance, Thailand, Disease prevention, Health policy, Public health

## Abstract

Dengue fever poses a significant public health burden in tropical regions, including Thailand, where periodic epidemics strain healthcare resources. Effective disease surveillance is essential for timely intervention and resource allocation. Various methods exist for spatiotemporal cluster detection, but their comparative performance remains unclear. This study compared spatiotemporal cluster detection methods using simulated and real dengue surveillance data from Thailand. A simulation study explored diverse disease scenarios, characterized by varying magnitudes and spatial-temporal patterns, while real data analysis utilized monthly national dengue surveillance data from 2018 to 2020. Evaluation metrics included accuracy, sensitivity, specificity, positive predictive value, and negative predictive value. Bayesian models and FlexScan emerged as top performers, demonstrating superior accuracy and sensitivity. Traditional methods such as Getis Ord and Moran’s I showed poorer performance, while other scanning-based approaches like spatial SaTScan exhibited limitations in positive predictive value and tended to identify large clusters due to the inflexibility of its scanning window shape. Bayesian modeling with a space–time interaction term outperformed testing-based cluster detection methods, emphasizing the importance of incorporating spatiotemporal components. Our study highlights the superior performance of Bayesian models and FlexScan in spatiotemporal cluster detection for dengue surveillance. These findings offer valuable guidance for policymakers and public health authorities in refining disease surveillance strategies and resource allocation. Moreover, the insights gained from this research could be valuable for other diseases sharing similar characteristics and settings, broadening the applicability of our findings beyond dengue surveillance.

**Supplementary Information:**

The online version contains supplementary material available at 10.1038/s41598-024-82212-1.

## Introduction

Dengue infection, transmitted by *Aedes* mosquitoes, continues to be a significant health concern in tropical and sub-tropical regions^1^. Thailand reports a substantial number of periodic annual cases^2^, imposing a considerable burden on the healthcare system and emerging as a major public health challenge^3,4^. The complex disease ecology of dengue, involving hosts, vectors, and various viral strains, coupled with challenges in diagnosis^5^, emphasizes the importance of understanding this health issue in endemic countries. Despite the adoption of new technologies and interventions by public health authorities worldwide, the limited efficacy of vaccination and the absence of a definitive treatment make outbreak detection crucial for guiding public health interventions , aiming to optimize resource allocation for vector control within budget constraints^6^.

Disease surveillance, an ongoing process generating and disseminating public health information, plays a pivotal role in informing policy and response measures^7^. For infectious disease outbreaks, timely information on case spread in space and time is essential for public health officials to take action. Space–time cluster detection methods are instrumental in monitoring the dynamic spread of infectious diseases, as they concurrently investigate time, place, and person^8–10^. However, despite extensive studies on spatial cluster techniques^11^, their applicability in dengue endemic countries, particularly Thailand, remains less evident. Previous studies on spatial cluster detection have not fully accounted for the inherent temporal and spatial components of health data^12,13^, highlighting the need for surveillance methods that encompass both dimensions^14–16^. Unlike previous studies that focused on spatial clustering, this paper emphasizes spatiotemporal cluster detection which is a critical approach for infectious diseases that display distinct transmission patterns. These patterns can vary widely in both the size and shape of clusters, such as persistent hotspots, short-lived outbreaks, and spreading waves of infection, each with different temporal dynamics. The time-sensitive nature of promptly detecting disease clusters is vital for implementing effective responses. Therefore, understanding these spatiotemporal dynamics is essential for devising effective control measures for dengue.

Effective disease surveillance is essential for informed policy planning, especially in resource-limited settings like Thailand, where understanding the spatial and temporal dynamics of disease transmission can greatly improve resource allocation and targeted interventions. However, there is a gap in knowledge about the spatiotemporal application of disease control tools, particularly in endemic settings. This study seeks to support public health authorities by conducting a comprehensive comparison of spatiotemporal anomaly detection methods, focusing on their applicability for dengue surveillance in regions where the disease is endemic.

To accomplish this, our investigation is divided into two parts: a simulation study and an analysis of real-world data. In the simulation study, we evaluated various widely-used space–time cluster detection techniques across a spectrum of disease scenarios. These scenarios were designed to reflect a range of possible transmission patterns observed in real-world contexts, such as isolated outbreaks in specific areas, widespread clusters affecting multiple regions simultaneously, and gradually expanding clusters. Each scenario varied in transmission duration, spatial extent, and intensity, representing realistic dynamics like localized outbreaks, persistent endemic regions, and temporary surges influenced by environmental or social factors. Following the simulation study, we applied the validated models to actual dengue surveillance data from Thailand, covering the period from 2018 to 2020. This allowed us to assess the practical utility of these methods in detecting and interpreting diverse disease patterns within a real public health context.

Therefore, the overall aim of this study is to evaluate the performance of different spatiotemporal cluster detection methods for dengue surveillance. Specifically, our objectives are to assess the effectiveness of these methods in identifying clusters across various transmission scenarios in the simulation study, and validate these methods with real-world dengue data from Thailand. This dual approach enables a comprehensive assessment of each method’s strengths and limitations in practical applications. We hope that our findings will serve as a resource for policymakers to select suitable spatiotemporal methods that can enhance dengue surveillance, identify health service gaps, and inform targeted interventions, particularly in resource-constrained settings. While focused on dengue surveillance in Thailand, our findings have broader implications and could be valuable for public health policy in other regions and for other diseases with similar transmission patterns.

## Methods

Various methods exist for spatiotemporal cluster detection; however, given that a substantial portion of public health surveillance data is in aggregate form, our study focuses primarily on areal cluster detection methods. These areal-based methods can be broadly classified into two categories: testing-based methods, which rely on fundamental hypotheses, and model-based approaches, exemplified in this study by hierarchical modeling. The selected methods are widely recognized cluster techniques for infectious diseases, as identified through systematic reviews^7,8^. Below, we provide an overview of the selected clustering models, along with the design and settings of our simulation study, including parameter configurations. An assessment of these methods is conducted using various evaluation metrics. For further details on the clustering methods and evaluation procedures, please refer to the supplementary documents S1.1–1.2.

### Spatiotemporal cluster detection methods

#### Anselin’s Local Moran’s I

Anselin’s Local Moran’s I is a localized version of the global Moran’s I statistic, specifically used to detect spatial autocorrelation within smaller areas of a study region^9^. It breaks down spatial association across the study area to identify localized clusters or outliers based on similarity or dissimilarity with neighboring values with a test on the null hypothesis of no local spatial association^9^. In practice, a high positive Local Moran’s I indicates a cluster (where similar high or low values group together), while a negative value suggests an outlier (a location with values unlike its neighbors). Using a quadrant plot, significant spatial units are categorized into four types: high-high clusters (HH), low–high outliers (LH), low-low clusters (LL), and high-low outliers (HL). For clusters and outliers to be considered statistically significant, they must pass a pre-specified significance threshold.

#### Getis-Ord Gi*

Getis-Ord Gi* is another method for detecting localized spatial clusters^10^, focusing on comparing a location’s attribute values to those of its neighbors to identify hotspots and cold spots^11^. High Gi* values indicate hotspots (regions with unusually high values), while low Gi* values mark cold spots (regions with low values). This is based on a comparison to the global average of the region under the null assumption of spatial independence^12^. Z-scores and p-values, generated through permutation testing, help to determine statistical significance.

#### Spatial and space–time scan statistic

The purely spatial scan statistic (SaTScan) is a method that scans a geographic region, such as a map of disease cases, to identify clusters of cases using circular or elliptical scanning windows of varying sizes. Each window evaluates whether the observed number of cases inside it is significantly higher or lower than expected by chance with the null hypothesis of no clusters^13^. This approach relies on a Poisson model, where the likelihood ratio for each window is calculated by comparing the observed case count within the window to the expected count based on the overall population distribution across the study area. By maximizing the likelihood ratio, the method identifies areas with significant spatial patterns, indicating clusters that may require further investigation for potential public health action.

Building upon the purely spatial scan, the space–time scan statistic (ST SaTScan) introduces a temporal dimension by using a cylindrical scanning window. In this approach, the circular base of the cylinder represents the spatial area, while the height of the cylinder corresponds to the time period. This cylindrical scanning window enables the detection of clusters that are not only spatially concentrated but also emerge over specific time intervals. As the cylinder scans across the study area and through different time frames, it identifies regions with unusually high case concentrations within specific temporal windows, highlighting potential outbreaks or time-sensitive clusters that may reflect seasonal patterns or emerging disease hotspots.

#### Flexible scan statistics (FlexScan)

Flexible scan statistics (FlexScan)^14,15^ are similar to SaTScan in identifying spatial clusters with the null hypothesis of no clustering, but differ by using more flexible scanning window shapes. Unlike SaTScan, which typically uses circular or elliptical windows, FlexScan employs irregularly shaped windows constructed by connecting adjacent regions. This flexibility allows the method to capture clusters that do not conform to simple geometric shapes. Note that for purely spatial methods such as SaTScan and FlexScan, which do not include a temporal component, spatiotemporal analyses have to be conducted iteratively at each time point to capture changes over time.

#### Spatiotemporal Bayesian modeling with exceedance probability

Bayesian statistical regression models have shown to be effective in analyzing epidemiological data with spatial and spatiotemporal characteristics^16,17^. A key advantage lies in their ability to consider uncertainty in estimates with the integration of spatial and temporal structures as prior distributions^8^. Moreover, this approach accommodates a broader range of conceptual models compared to non-Bayesian methods^18^. Generally, in this model, the number of cases in each location and time period is analyzed by estimating the relative risk of disease. The model includes spatial and temporal components: spatial effects capture geographic variability, while temporal effects account for changes over time. Additionally, an interaction term can represent the combined influence of space and time.

A key feature of Bayesian modeling is the use of exceedance probabilities to identify “hotspots” or areas with unusually high disease risk^19,20^. The exceedance probability is the likelihood that the risk in a given area exceeds a specific threshold (often set to the expected rate or baseline). Locations where this probability surpasses a predetermined significance level are flagged as hotspots, indicating areas of concern. To make the model computationally feasible, especially with the high-dimensional data of real-time disease surveillance, we used Integrated Nested Laplace Approximation (INLA)^21^. INLA is an efficient alternative to traditional methods like Markov Chain Monte Carlo (MCMC) for Bayesian inference, allowing for faster analysis. This enabled us to implement the model in the R-INLA package, which supports the integration of Bayesian models for timely disease surveillance.

### Study design and data source

In this study, we undertook a comprehensive assessment of spatiotemporal cluster detection methods, using both a simulation study and a real case study using monthly surveillance data at provincial level. Simulation studies are instrumental for evaluating and contrasting spatial detection methods, offering the advantage of understanding the behavior of statistical techniques by manipulating known values of parameters during data generation^22^. Then, in the initial phase, we conducted a simulation study with various scenarios to represent outbreak situations. This approach allows us to explore and gain insights into method properties through controlled, ground-truth scenarios.

Following the simulation, we demonstrated the applicability of the examined methods through a case study utilizing actual monthly dengue surveillance data from the Division of Epidemiology, Department of Disease Control, Thai Ministry of Public Health (https://ddc.moph.go.th/doe/). This approach provides a comprehensive investigation of spatiotemporal anomaly detection and offers valuable insights for national programs seeking effective tools for dengue surveillance. The dengue case data was sourced from the national surveillance reporting with all cases reported by local health authorities and aggregated monthly at the provincial level. This dataset includes total cases across all severity levels of dengue, encompassing probable, suspected, and confirmed cases, as defined by the surveillance case definition of the Division of Epidemiology, Department of Disease Control^23^.

### Simulation study

#### Simulation scenario and data generation

**Fig. 1:**
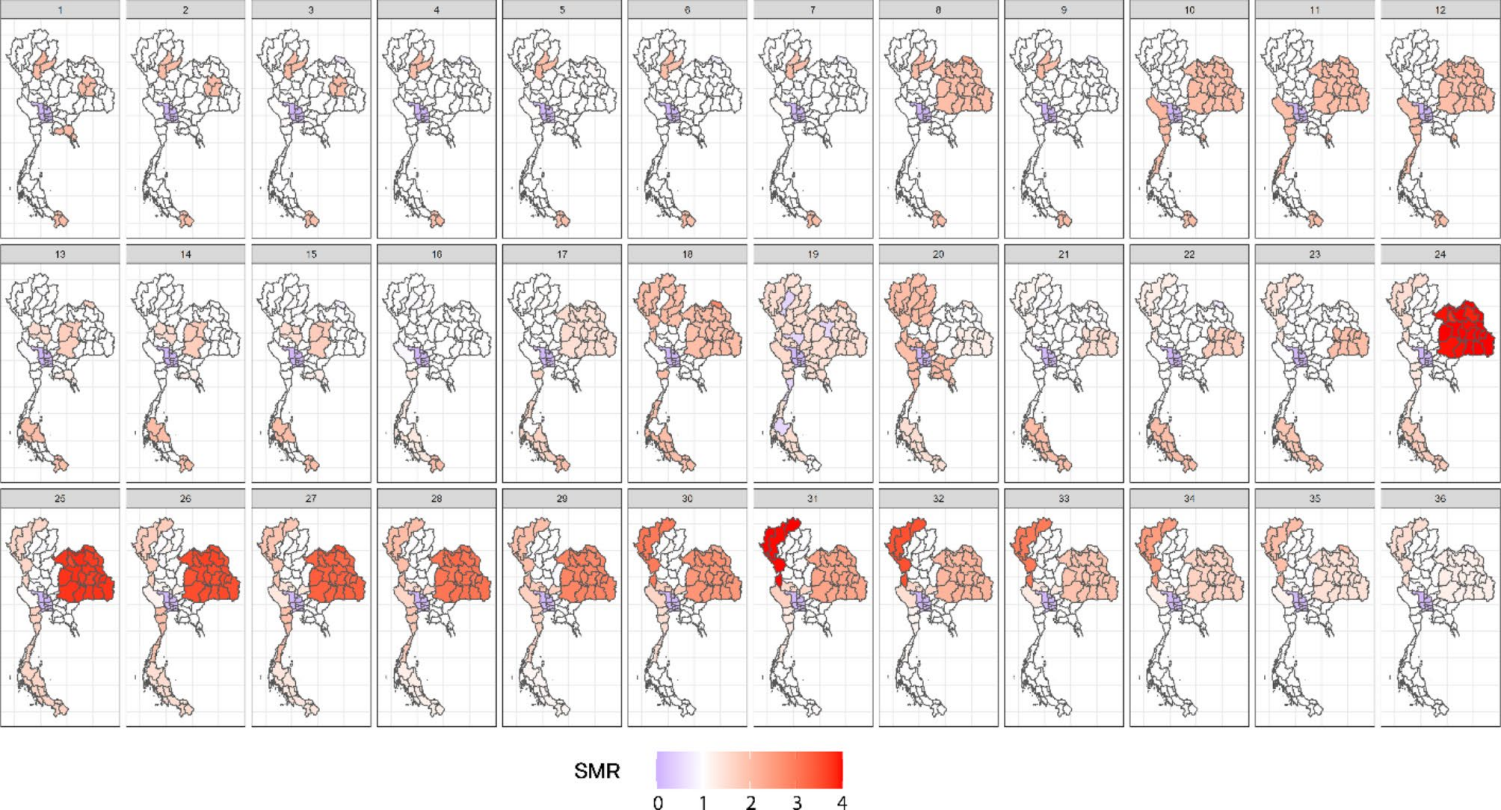
Maps of simulated relative risks in the simulation study, generated using RStudio version 2022.07.0 + 548 (available at https://posit.co/products/open-source/rstudio/).

The simulation study and application were conducted on a map of Thailand with its 77 provinces, as illustrated in Fig. 1. The risk magnitudes in simulated scenarios were represented by mean relative risk values at the provincial level. The goal was to create diverse scenarios representing different disease transmission situations over a 36-month period. These scenarios were designed to reflect the diverse disease transmission situations observed in real-world data, based on patterns seen in previous analyses, as demonstrated in the case study with real dengue incidence data in Thailand. The dengue patterns varied in size and shape, with both large clusters and isolated hotspots. Therefore, the simulations were designed to reflect the real-world patterns of dengue incidence observed in Thailand from historical surveillance data.

In the case of space–time isolated clusters, we simulated three provinces in the lower part of southern Thailand as a stable disease cluster with a relative risk of 2, lasting for 18 months. This value reflects a moderate risk scenario based on the typical peaks in dengue incidence observed in surveillance data. We also introduced a cluster in the northeastern region with a stable relative risk of 2 for the first three months and then for an additional five months. A significant cluster in the northeastern and western regions was sustained with a relative risk of 2 until month 12. Following this, a dynamic disease pattern occurred in the southern region between months 13 and 18, where some provinces exhibited dispersal and anomalies. These outbreaks in the upper-central and northeastern regions were characterized by central separation, reflecting the observed heterogeneity in dengue outbreaks in these regions.

For timesteps 18 through 20, we simulated variations in disease patterns inspired by potential changes in prevention policies, either strict or relaxed, with the situation eventually reverting to the initial stage. At timestep 21, we introduced a gradient of disease relative risk. In the northeastern region, the risk started at 4 (representing a high-risk outbreak) and gradually decayed to nearly 1 by the last timestep, simulating a controlled decline in disease incidence. Meanwhile, a triangular gradient emerged in the northern region, where the relative risk started at 1 and peaked at 4 by timestep 31. This scenario represents an increasing risk pattern in one region while other regions experience containment. By the final period, we assumed that effective government surveillance and intervention policies had been implemented, bringing the relative risk across all regions down to nearly 1.

**Fig. 2:**
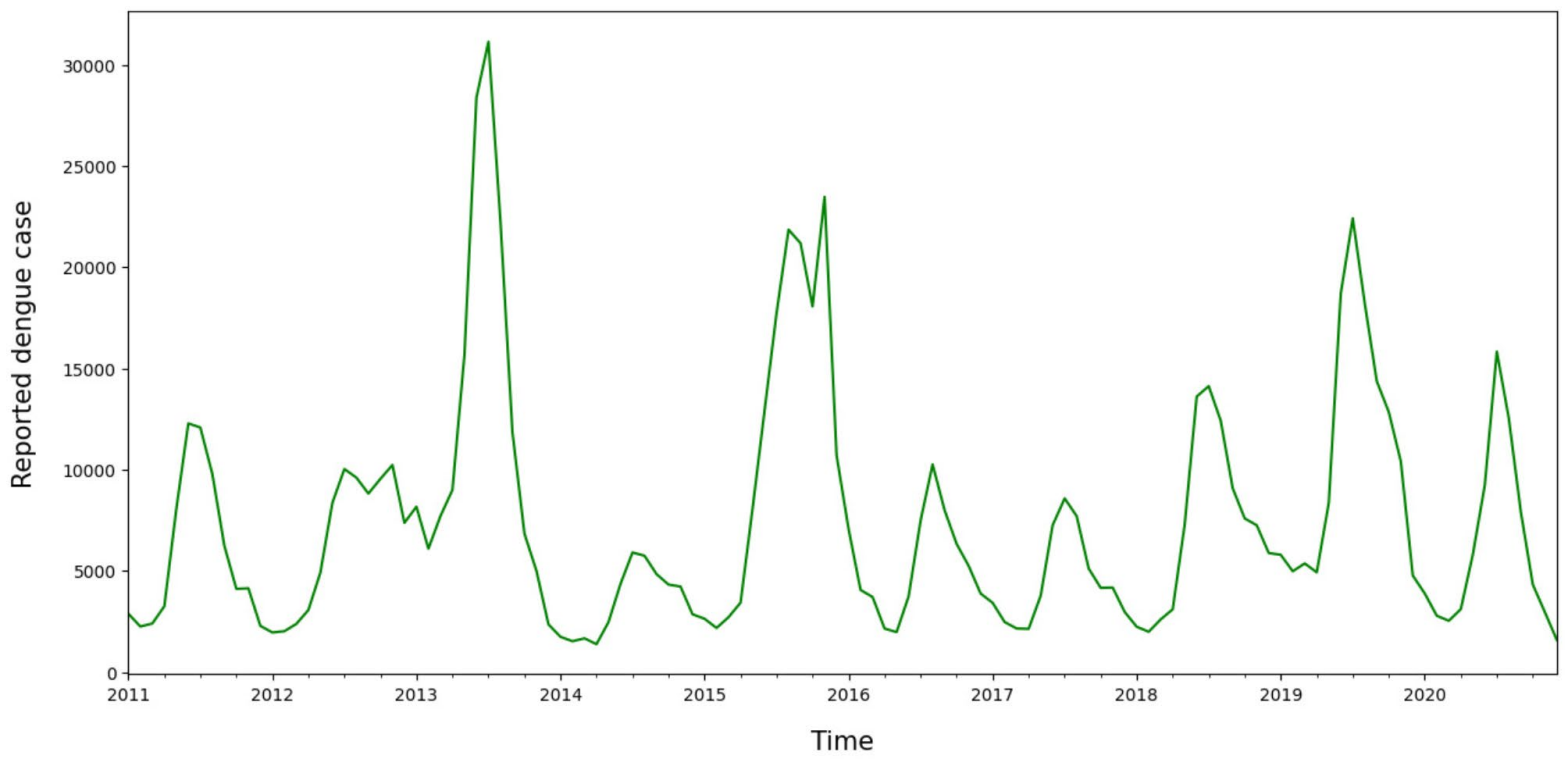
Plot of monthly dengue case reported to the Thai national dengue surveillance program during 2011–2020, created using RStudio version 2022.07.0 + 548 (available at https://posit.co/products/open-source/rstudio/).

Figure 1 shows maps of simulated relative risks in the simulation study. The relative risk values selected, ranging from 1 to 4, reflect different epidemic magnitudes and outbreak sizes of dengue in Thailand. These values were chosen based on historical fluctuations in dengue case counts, as seen in Fig. 2, which illustrates the temporal monthly incidence trends from 2011 to 2020. These trends show variability in peak sizes, with peaks reaching up to 3 times the baseline risk. To evaluate detection methods’ performance under varying conditions, we generated simulated data with different outbreak locations, magnitudes, and extreme values to mimic potential real-world dengue scenarios that have occurred in the past and could happen in the future. These simulations allowed us to test the methods’ ability to detect outbreaks of varying intensity and duration.

Two hundred replications of cases count were generated corresponding to the designed relative risk values. Dengue cases ${y}_{kit}$ were then simulated using a Poisson distribution as ${y}_{kit}\sim Poisson\left({E}_{kit}{\theta }_{kit}\right)$, where ${E}_{kit}$ and ${\theta }_{kit}$ represented expected rate and relative risk value over replication $k$ at location $i$ and time $t$. Expected rates on annually repeated timesteps of the simulation study were calculated from real dengue surveillance case numbers and population in the year 2017, serving as the baseline. This was determined by setting ${E}_{kit}={E}_{it}=\frac{{\Sigma }_{j}{y}_{jt}}{{\Sigma }_{j}po{p}_{jt}}*po{p}_{it}$, where is the *pop*_*it*_ is the population in location *i* at time period *t*, which was obtained from the Thai Bureau of Registration Administration, Department of Provincial Administration, Ministry of Interior.

#### Method specification and evaluation metrics

The provincial boundaries of Thailand used in this study were obtained from the Global Administrative Areas (GADM) database (https://gadm.org/). To compute the spatial matrix, we utilized the queen contiguity criterion, as previously demonstrated^24^. The spatial weight matrix, based on the queen contiguity criterion, was calculated using spdep version 1.2–8. For each simulated iteration *k*, we performed Getis Ord Gi* and local Moran’s I on the standardized morbidity ratio (SMR) for the *i* th province, calculated as *SMR*_i_ = *y*_i_/*e*_i_. A permutation distribution was used to determine statistical significance, implemented using spdep version 1.2–8 within Python version 3.10.4^25^.

FlexScan was implemented by structuring a Poisson likelihood that incorporated a spatial weight matrix and isolated values of both observed and expected quantitation as input parameters. The analysis employed standard circular investigation zones along with flexible window shapes, utilizing the Monte Carlo test through the rflexscan package, version 1.1.0^26^. Conversely, SaTScan analysis was conducted using both circular and elliptical scanning shapes for window configurations. The investigation areas were delineated based on the population at risk, with the size of the scanning zone capped at a maximum population of 50%. The calculations were performed using the rsatscan library version 1.0.5 in conjunction with the base command-line interface version 10.1^27^. All the testing-based methods were conducted at a significance level of 0.05.

In spatiotemporal Bayesian modeling, we formulated combinations of spatial, temporal, and interaction settings to estimate the predicted relative risk based on case observations and expected rates. The total number of model specifications amounted to 36, encompassing three spatial random effects: independent and identically distributed (IID), Besag, and convolution models, as well as three temporal random effects: IID, RW1, and RW2. Anomalies were defined using exceedance probability $\text{Pr}\left({\theta }_{kit}>{\theta }^{*}\right)>1-\alpha$ where ${\theta }^{*}$ was relative risk ratio and $\alpha$= 0.05 was level of significance threshold. All forms of Bayesian models were executed using INLA library version 23.04.24 (https://www.r-inla.org/).

To compare the performance of all anomaly detection methods, we conducted prospective computations to evaluate space–time disease surveillance methods. For spatial methods including Getis Ord, Moran’s I, purely spatial SatScan, FlexScan and Bayesian modeling with either spatial or temporal terms alone, the methods were applied iteratively for each time point. In contrast, for space–time SaTScan, which operates retrospectively, we compared both prospective and retrospective approaches. When conducting prospective analysis, only the last time point for each step was retained. Then we assessed their effectiveness using computing time and various goodness-of-fit metrics, including accuracy, sensitivity, specificity, positive predictive value (PPV), negative predictive value (NPV), and computing time. Additionally, Bayesian modeling was evaluated using the widely applicable or Watanabe-Akaike information criterion (WAIC)^28^. All models and methods were computed at a significance level of α = 0.05. The evaluation metrics can be found in the supplementary document S1.2.

## Results

### Simulation study

**Fig. 3:**
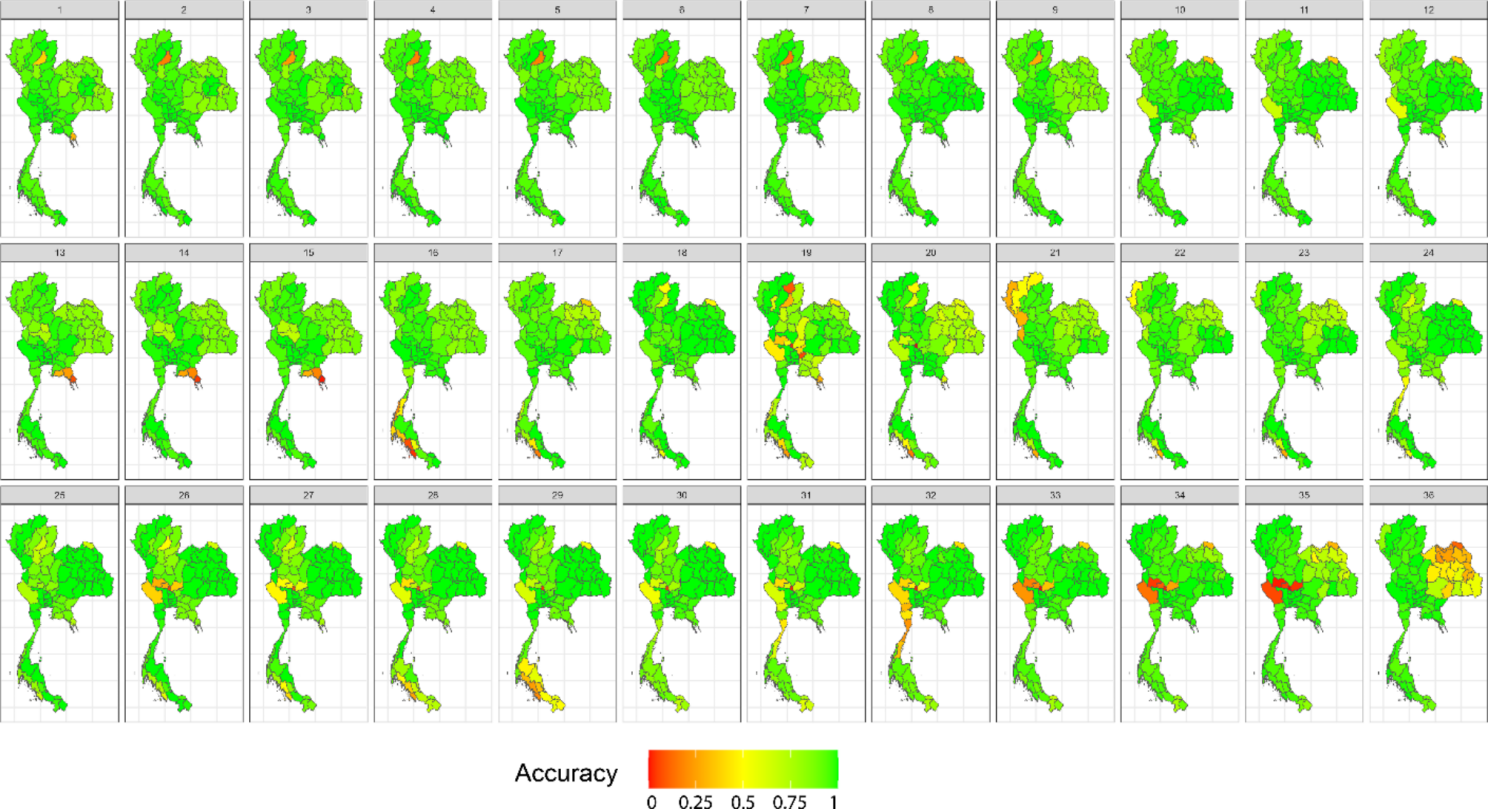
Accuracy maps of the prospective Bayesian model with BESAG and random walk order 2 effect terms, generated using RStudio version 2022.07.0 + 548 (available at https://posit.co/products/open-source/rstudio/).

**Fig. 4:**
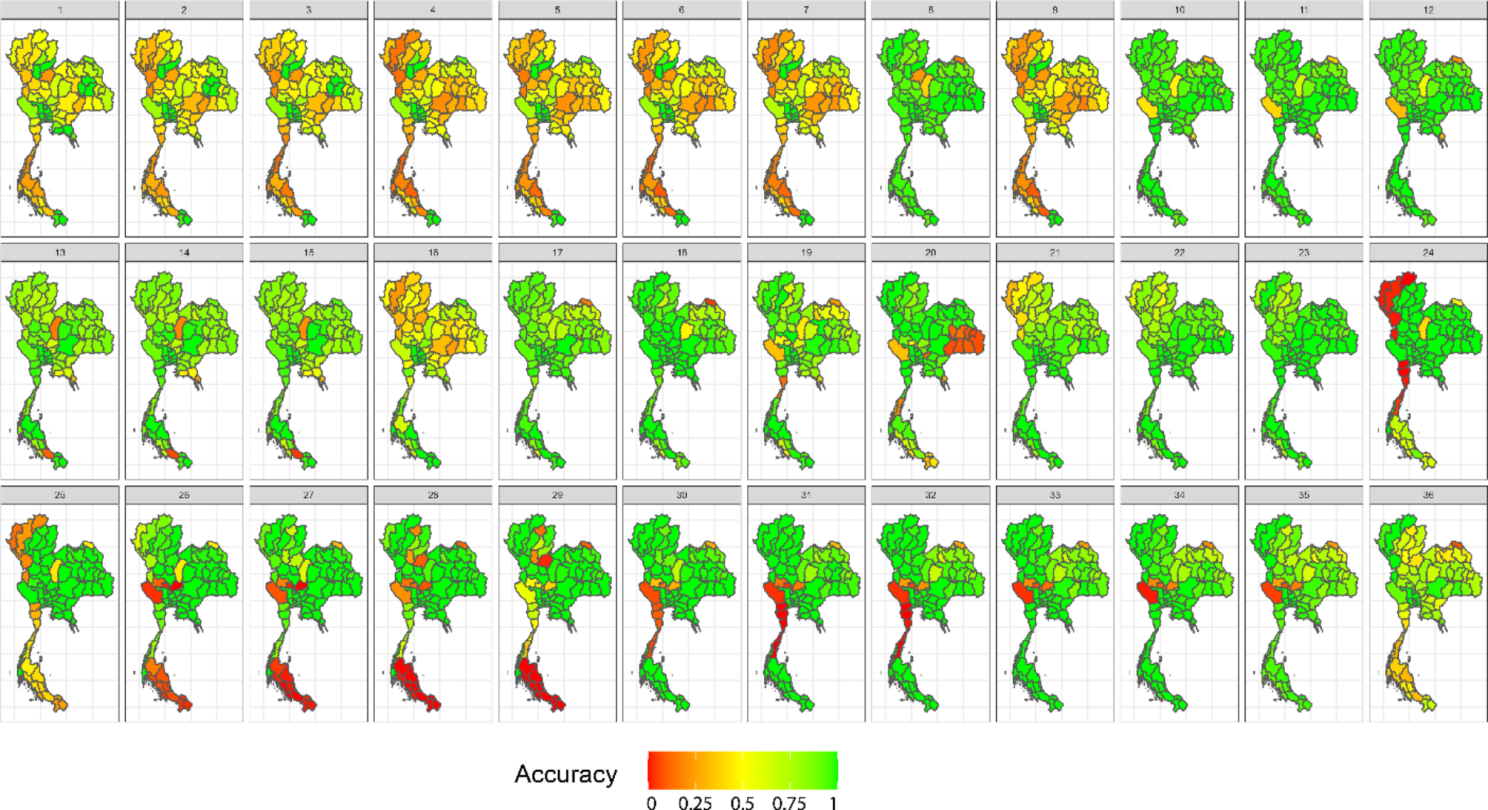
Accuracy maps of the FlexScan with flexible scanning window, generated using RStudio version 2022.07.0 + 548 (available at https://posit.co/products/open-source/rstudio/).

**Fig. 5:**
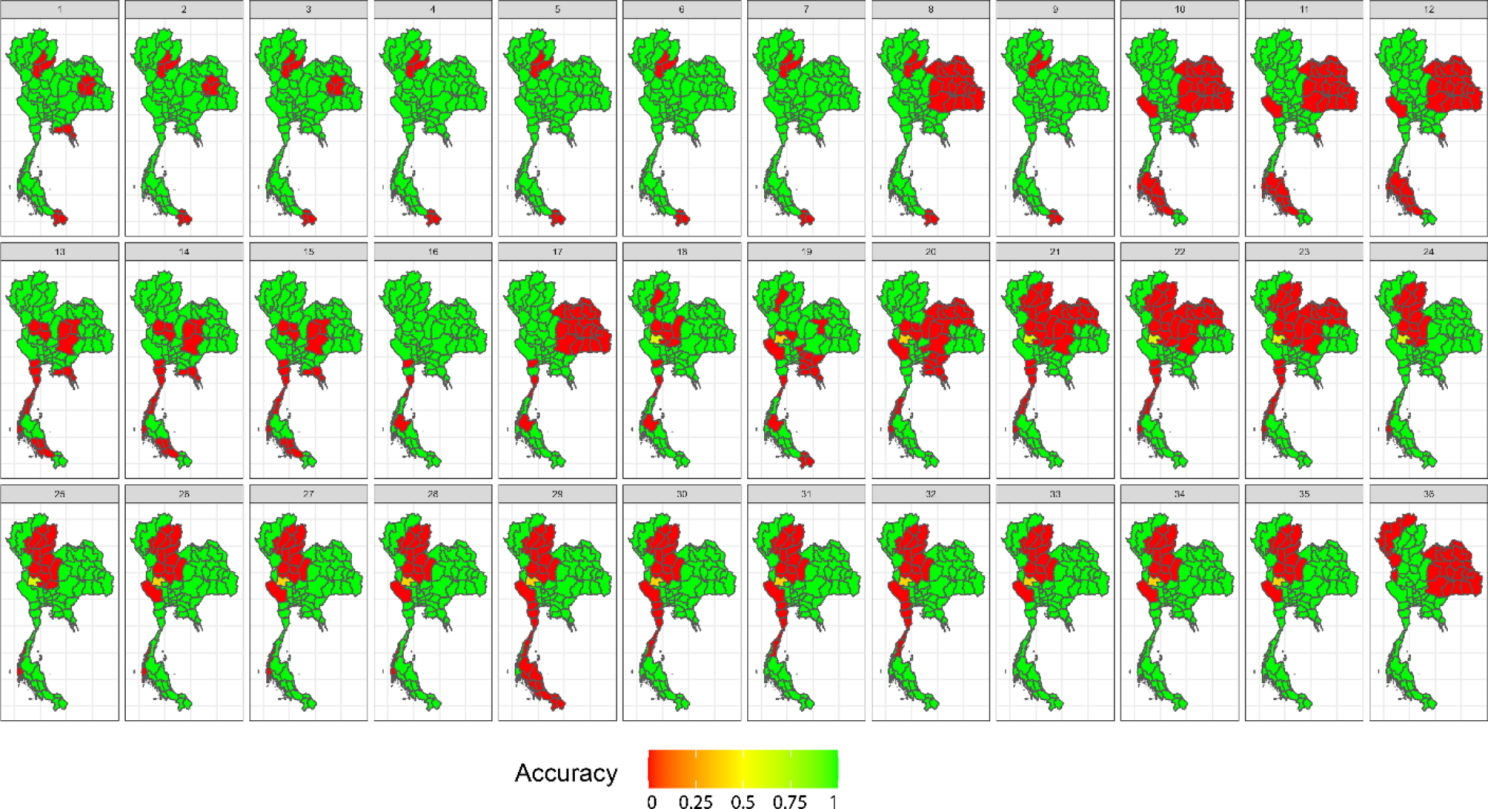
Accuracy maps of the retrospective space–time SaTScan model with elliptical scanning window, generated using RStudio version 2022.07.0 + 548 (available at https://posit.co/products/open-source/rstudio/).

**Fig. 6:**
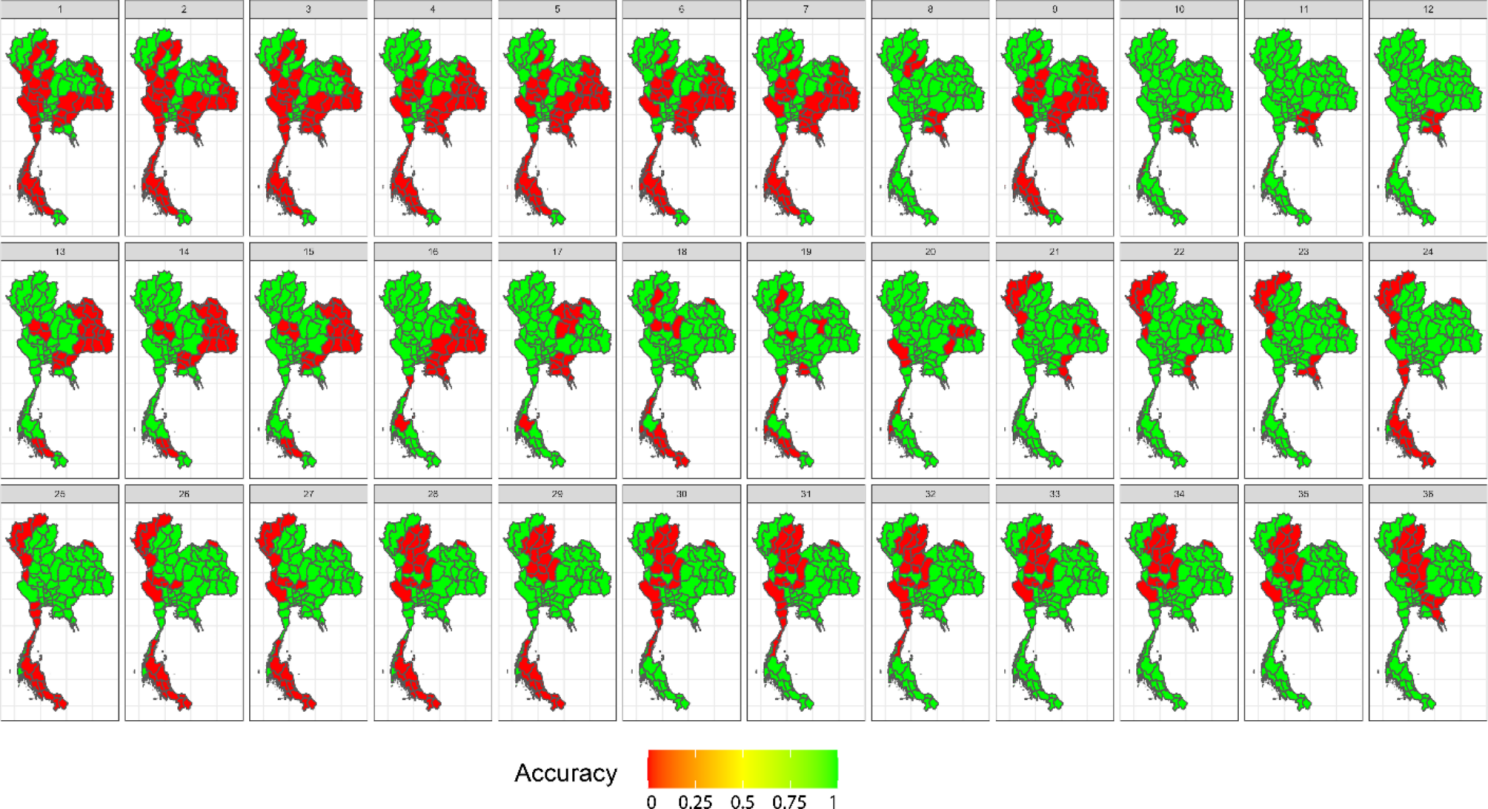
Accuracy maps of spatial SaTScan model with elliptical scanning window, generated using RStudio version 2022.07.0 + 548 (available at https://posit.co/products/open-source/rstudio/).

In our simulation study, we assessed widely used space–time cluster detection methods based on key performance metrics, including average computation time per time step (in seconds), accuracy, positive predictive value, sensitivity, specificity, and negative predictive value. Table 1 provides a comprehensive comparison of the average performance metrics across locations and time periods, offering a summary of how each method performed across different evaluation criteria. In addition, Figs. 3, 4, 5 and 6 display the provincial-level accuracy over time for the best-performing method in each category. However, further detailed simulation results are available in supplementary document S2.

**Table 1:** Performance evaluation of space–time cluster detection methods in the simulation study, presented across key metrics.

Method	Type	Computing (sec.)	Accuracy	PPV	Sensitivity	Specificity	NPV	Spatial	Temporal	Interaction
ST Bayesian	Space–time	1.5333	0.8968	0.9168	0.9246	0.8456	0.8590	BESAG	RW2	Type I
FlexScan circular	Spatial	2.2008	0.7880	0.6616	0.7712	0.7967	0.8710	–	–	–
FlexScan flexible	Spatial	2.1616	0.7879	0.6614	0.7711	0.7965	0.8709	–	–	–
ST SaTScan elliptic (Retrospective)	Space–time	3.0917	0.7620	0.6373	0.6973	0.7954	0.8360	–	–	–
Spatial Bayesian	Spatial	1.7649	0.7329	0.9101	0.6521	0.8816	0.5794	BYM	–	–
Spatial Bayesian	Spatial	0.9721	0.7313	0.9108	0.6488	0.8832	0.5775	IID	–	–
ST SaTScan circular (Retrospective)	Space–time	0.0987	0.7257	0.5801	0.7015	0.7382	0.8275	–	–	–
SaTScan elliptic	Spatial	0.7788	0.7042	0.5468	0.7614	0.6747	0.8458	–	–	–
ST Bayesian (no interaction)	Space–time	1.1964	0.6750	0.9527	0.5243	0.9522	0.5211	BYM	IID	–
SaTScan circular	Spatial	0.6127	0.6732	0.5120	0.8367	0.5888	0.8749	–	–	–
ST SaTScan elliptic (Prospective)	Space–time	8.2628	0.6688	0.9630	0.0276	0.9995	0.6659	–	–	–
ST SaTScan circular (Prospective)	Space–time	0.9897	0.6598	-	-	0.9998	0.6598	–	–	**–**
Local Getis	Spatial	0.0634	0.6273	0.4645	0.6243	0.6289	0.7645	–	–	–
Local Moran	Spatial	0.0687	0.6258	0.4629	0.6235	0.6270	0.7636	–	–	–
Temporal Bayesian	Temporal	0.9329	0.6068	0.8070	0.5166	0.7727	0.4649	–	RW1	–

The average computation time is reported for all time steps. Abbreviations: ST = spatiotemporal, PPV = positive predictive value, NPV = negative predictive value.

Among all the methods evaluated, the spatiotemporal Bayesian models stood out, particularly the model combining a spatial Besag model with a temporal random effect using an random walk of order 2 (RW2) prior and a type I interaction term. This model exhibited a computation time of 3.1654 s and achieved the highest accuracy of 0.8953, along with notable PPV, sensitivity, and specificity. We then further examined spatial Bayesian models with only the spatial term to gain insights into the impact of spatial structure and the lack of a temporal term on accuracy. With only the BYM term, the model achieved a reduced accuracy of 73.29%, with a reasonably short computation time of 1.8688 s. Conversely, the IID term model had a slightly lower accuracy at 73.13%. Additionally, the Bayesian model with the temporal term, which performed best with an RW1 term, achieved an accuracy of 60.68%. This highlights the importance of including interaction terms, which contributed to the robust performance of the spatiotemporal Bayesian model in space–time cluster detection. Conversely, the absence of an interaction term in the space–time Bayesian model resulted in reduced accuracy and sensitivity, highlighting the trade-offs associated with model complexity.

Following the Bayesian model, FlexScan with circular and flexible scanning shapes showed competitive performance, with computation times of 2.3303 and 2.2888 s, respectively, and notable accuracy rates of 78.80% and 78.79%. The circular FlexScan model demonstrated a sensitivity of 66.16% and a specificity of 77.12%, along with a positive predictive value of 79.67% and a negative predictive value of 87.1%. Similarly, the flexible scanning shape achieved a sensitivity of 66.14% and a specificity of 77.11%, with corresponding positive and negative predictive values of 79.65% and 87.09%, respectively. For the space–time SaTScan models, the circular retrospective method achieved an accuracy of 72.57%, with a sensitivity of 58.01% and a specificity of 73.82%, while the retrospective elliptic method achieved an accuracy of 76.20% with a sensitivity of 69.73%. The circular model had a short computation time of 0.1045 s, while the elliptic model required 3.2735 s. Prospective analysis revealed that the elliptic shape had a moderate accuracy of 66.88% with a computation time of 8.7488 s, whereas the circular shape exhibited lower accuracy at 65.98% but faster computation, taking only 0.989 s. Note that the prospective space–time SaTScan had a true positive rate equal to zero, resulting its sensitivity and PPV unable to be calculated.

For purely spatial SaTScan, the elliptic model achieved an accuracy of 70.42%, a sensitivity of 54.68%, and a specificity of 76.14%, with positive and negative predictive values of 67.47% and 84.58%, respectively. The circular model had a slightly higher accuracy of 67.32%, a sensitivity of 51.20%, and a specificity of 83.67%, with corresponding positive and negative predictive values of 58.88% and 87.49%, respectively. Regarding local Getis, it attained an accuracy of 62.73%, a sensitivity of 46.45%, and a specificity of 62.43%, with positive and negative predictive values of 62.89% and 76.45%, respectively. On the other hand, the local Moran method achieved an accuracy of 62.58%, a sensitivity of 46.29%, and a specificity of 62.35%, with positive and negative predictive values of 62.70% and 76.36%, respectively.

In summary, spatiotemporal Bayesian models emerged as the top performers, achieving nearly 90% accuracy. Spatial Bayesian models lacking temporal terms yielded accuracies around 73%, while those with temporal terms achieved 60.68% accuracy, emphasizing the significance of space–time interaction terms. FlexScan demonstrated competitive accuracy rates of approximately 78%. Retrospective circular and elliptic space–time SaTScan models achieved accuracies of 72.57% and 76.20%, respectively, with prospective space–time SaTScan showing reduced accuracy. In purely spatial SaTScan, the elliptic model outperformed the circular model in accuracy. The local Getis and Moran methods exhibited the lowest accuracy but similar predictive values in the simulation study.

Given the notable performance of Bayesian models, we further examined their characteristics. Table 2 details the top ten Bayesian models ranked by accuracy, providing insights into their characteristics and performance metrics. This investigation highlights the widespread use of type I interaction terms in high-performing models, along with spatial and temporal components, present in the majority of models featuring type I interaction. Notably, models with interaction exhibited consistent and commendable accuracy, reaching approximately 89%. Conversely, the model without interaction displayed the lowest accuracy among the top ten, achieving 73%. Sensitivity effectively differentiated Bayesian models based on their interaction term, with values around 0.92 with interaction and 0.65 without interaction. Interestingly, the specificity for the model without interaction surpassed other candidates at 88%, compared to an average of approximately 84% for models with interaction. NPV values were 0.85 for models with interaction and 0.57 for models without interaction, while PPV remained consistent across all top ten models at 91%.

**Table 2:** Top 10 Bayesian models ranked by accuracy, along with their corresponding performance metrics from the simulation study.

Spatial term	Temporal term	WAIC	Computing (sec.)	Accuracy	PPV	Sensitivity	Specificity	NPV
BESAG	RW2	316226.2325	1.5333	0.8968	0.9168	0.9246	0.8456	0.8591
BYM	RW2	316323.8645	2.5002	0.8962	0.9165	0.924	0.8452	0.8582
BESAG	IID	316133.7689	1.7582	0.896	0.9155	0.9248	0.8431	0.8591
BESAG	RW1	316190.0263	1.8268	0.8958	0.9172	0.9225	0.8468	0.8559
BYM	IID	316240.2233	2.9335	0.8955	0.9154	0.9241	0.8429	0.8579
BYM	RW1	316294.4386	2.9895	0.8953	0.9170	0.9218	0.8466	0.8547
IID	RW2	316464.0634	1.6395	0.8952	0.9176	0.9209	0.8478	0.8535
IID	IID	316366.6433	1.7659	0.8943	0.9162	0.9211	0.8451	0.8534
IID	RW1	316430.1811	1.6919	0.8941	0.918	0.9186	0.8490	0.8501
BESAG	-	739465.0954	0.9525	0.7330	0.9100	0.6525	0.8813	0.5796

The computation time is averaged over all time steps. Abbreviations: PPV = positive predictive value, NPV = negative predictive value, IID = independent and identically distributed, BYM = Besag, York, and Mollié model, WAIC = Watanabe-Akaike information criterion, RW1 and RW2 = random walk effects of order 1 and 2.

The observed heightened performance metrics among models with interaction terms underscore their effectiveness in capturing complex spatiotemporal patterns. The inclusion of type I interaction consistently contributes to enhanced accuracy and other evaluation metrics, making it a crucial factor for the overall performance and reliability of Bayesian models in disease surveillance and cluster detection. Models with interaction terms demonstrated very similar accuracy, around 90%, as shown in Table 2. Although adding the interaction term increases the computing time, the average computing time remained relatively low across all methods. Therefore, incorporating the space–time interaction term in applications might be worthwhile, as it significantly increases accuracy while maintaining reasonable computational efficiency.

### Application with dengue incidence data in Thailand

**Fig. 7:**
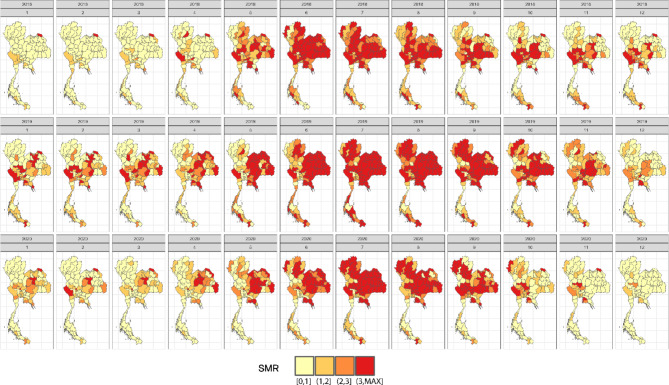
Monthly provincial maps of standardized morbidity ratios for dengue from the national surveillance data from 2018 to 2020. Created using RStudio version 2022.07.0 + 548 (available at https://posit.co/products/open-source/rstudio/).

Our study initially explored in a simulation study with diverse scenarios reflecting the dynamics of dengue transmission in Thailand. In this section, we extended our investigation to real dengue surveillance data. Utilizing actual national dengue surveillance data from the Thai Ministry of Public Health, we analyzed monthly incidence between 2018 and 2020, including data from 2017 for baseline rate calculation. Figure 7 illustrates the monthly SMRs of dengue incidence from the national surveillance data during 2018 to 2020, highlighting the seasonal transmission patterns, which typically peak during the rainy season from May to October^29^.

This seasonal pattern is also captured in our simulations, which were designed to capture these peaks and the spatial distribution of dengue cases as observed in the real data. Moreover, we introduced additional scenarios in the simulations to represent potential future outbreaks, characterized by higher relative risks and wider spatial spread compared to the historical data. These scenarios allowed us to assess the robustness of the cluster detection methods, particularly their ability to identify emerging hotspots swiftly, while still maintaining a pattern similar to national surveillance data.

**Fig. 8:**
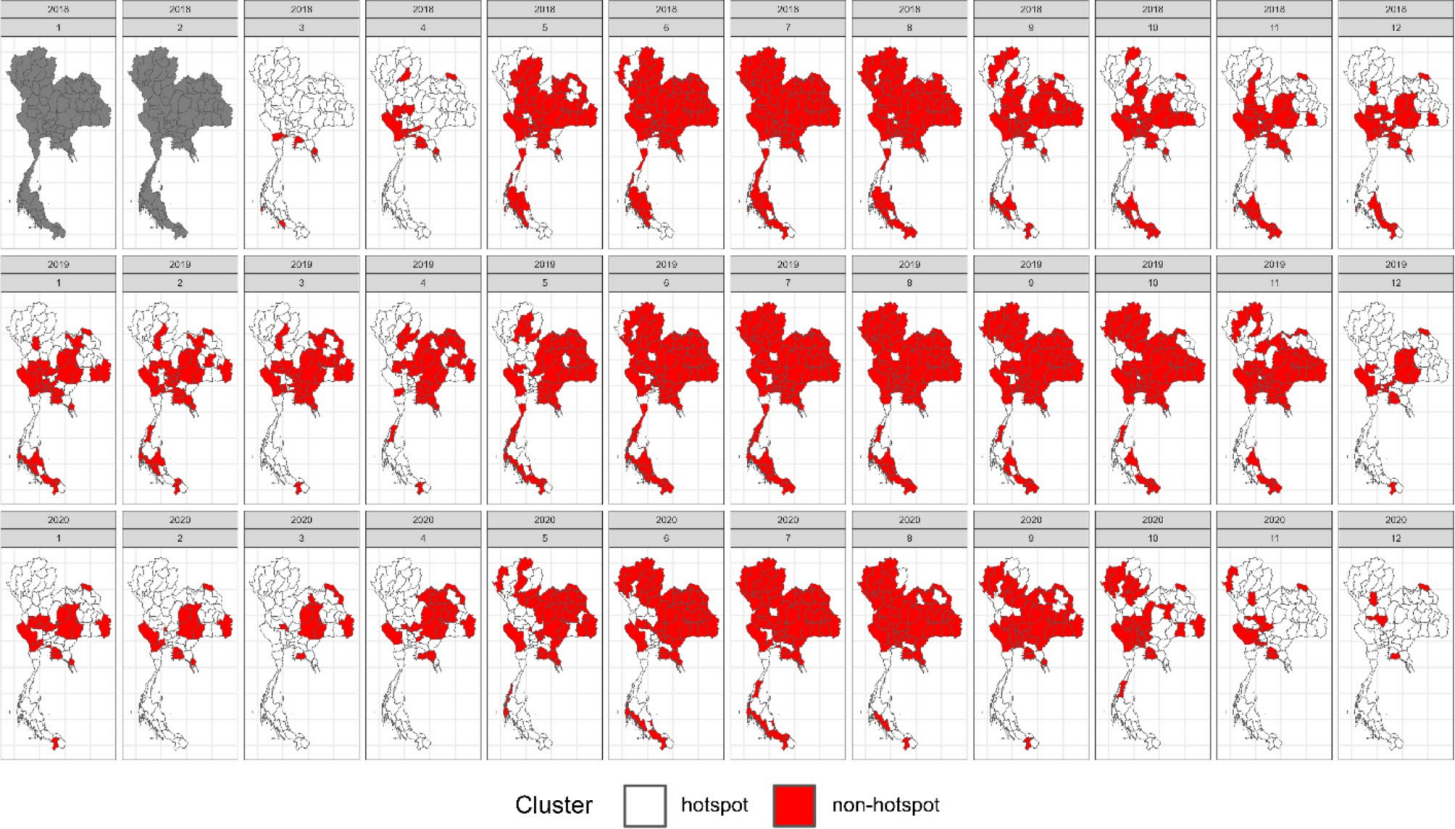
Provincial hotspot maps of spatiotemporal Bayesian model using monthly national reported dengue data in Thailand during 2018–2020, generated using RStudio version 2022.07.0 + 548 (available at https://posit.co/products/open-source/rstudio/).

**Fig. 9:**
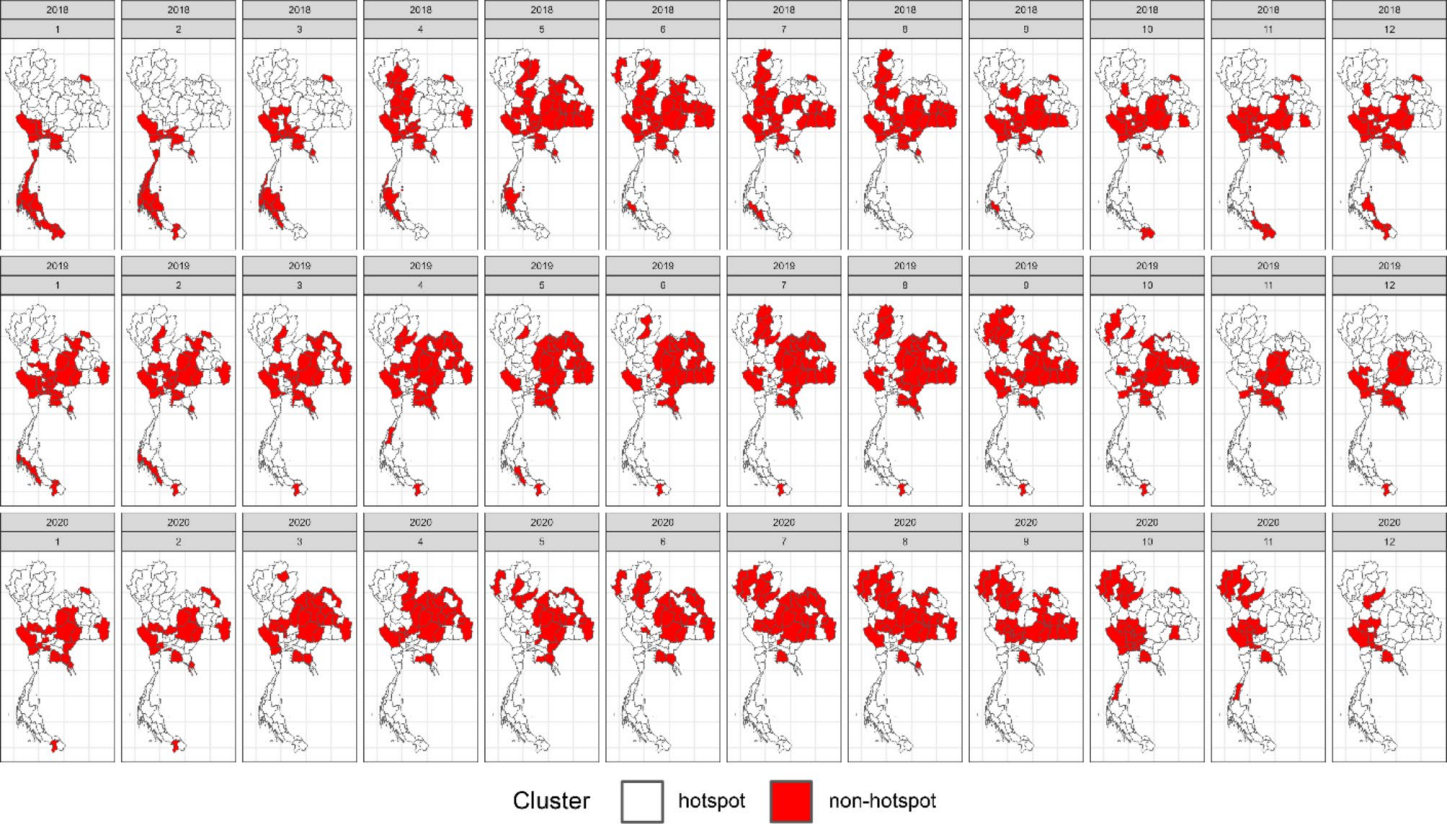
Provincial hot spot maps of FlexScan with flexible shape using monthly national reported dengue data in Thailand during 2018–2020, generated using RStudio version 2022.07.0 + 548 (available at https://posit.co/products/open-source/rstudio/).

**Fig. 10:**
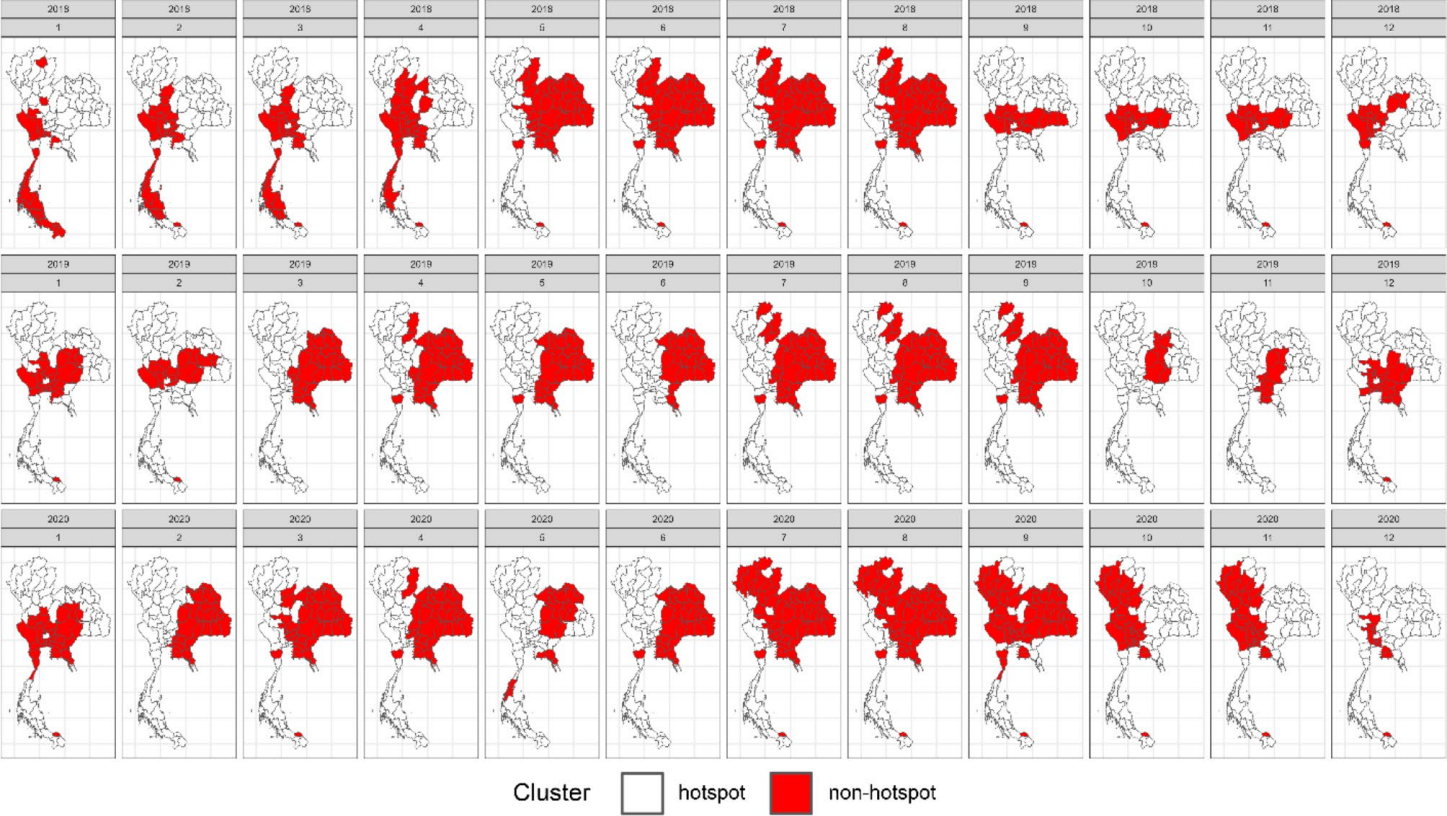
Provincial hot spot maps of spatial SaTScan with elliptical shape using monthly national reported dengue data in Thailand during 2018–2020, generated using RStudio version 2022.07.0 + 548 (available at https://posit.co/products/open-source/rstudio/).

**Fig. 11:**
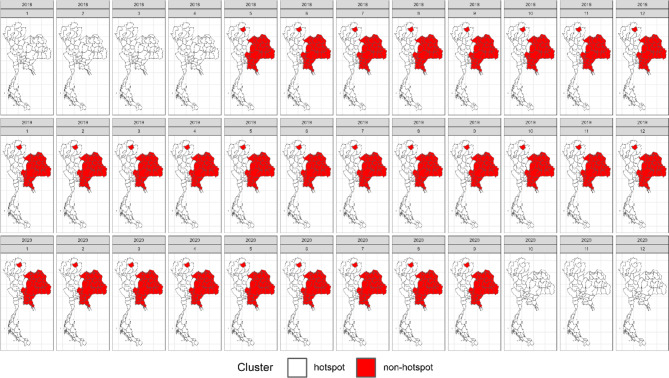
Provincial hot spot maps of prospective space–time SaTScan with elliptical shape using monthly national reported dengue data in Thailand during 2018–2020, generated using RStudio version 2022.07.0 + 548 (available at https://posit.co/products/open-source/rstudio/).

In line with our simulation study, we implemented the same model and method setup with a significance level of *α* = 0.05 to our case study, aiming to assess each method’s performance in real-world scenarios. Figures 8, 9, 10 and 11 illustrate provincial hotspot maps generated by the best-performing method, identified through its superior accuracy in the simulation study, utilizing monthly national reported dengue cases in Thailand from 2018 to 2020. Note that the first two grey maps are the burn-in period due to the use of RW2 as the temporal random effects. Supplementary document S3 provides additional results of the remaining models and methods applied to this real data example. In this case study of real dengue surveillance data, Bayesian models demonstrated remarkable performance, consistent with the findings of the simulation study. However, we chose to apply only the overall best-performing model from the simulation study in this case. Figure 8 showcases the Bayesian model characterized by Besag as the spatial term, RW2 as the temporal residual, and spatiotemporal interaction Type I, which achieved the highest accuracy in the simulation study while maintaining a balance between accuracy and computational efficiency.

When examining the spatiotemporal cluster detection methods, the Bayesian model (Fig. 8) and FlexScan (Fig. 9) produced results consistent with the observed SMR patterns shown in Fig. 7. Both models detected the highest concentration of dengue clusters during the rainy season, with fewer and more scattered clusters during the summer and winter months. This aligns with the real data, where dengue cases also spike during the rainy season. In contrast, the results from the purely spatial and prospective space–time SaTScan methods (Figs. 10 and 11) were less aligned with the observed dengue transmission patterns. The spatial SaTScan method identified large clusters that somewhat resembled the actual incidence data, as seen in Fig. 10. However, the space–time SaTScan (Fig. 11) detected only a few clusters across the study period, overlooking the majority of the small-to-moderate sized clusters that were captured by both the Bayesian model and FlexScan. This underestimation of clusters resulted in inconsistencies with the SMRs derived from the real data.

Additionally, the results of the Getis Ord Gi* and Local Moran’s I methods (provided in the supplementary document S3) identified a limited number of small clusters throughout the study period. These clusters were inconsistent with the actual SMR patterns observed in the real dengue data, further indicating that these methods may not be as effective in detecting the dynamic, space–time progression of dengue outbreaks. Overall, our results indicate that the Bayesian model and FlexScan outperformed other methods in capturing the spatial-temporal dynamics of dengue incidence. These methods were particularly adept at identifying clusters that closely matched the observed patterns in the national dengue surveillance data. Furthermore, the findings from this real-case application are consistent with the performance of the detection methods evaluated in our earlier simulation study.

## Discussion

Dengue presents substantial economic and public health challenges, particularly in tropical countries like Thailand. Diverse cluster detection methods have been employed in numerous studies and by dengue control programs. However, determining the most effective space–time cluster detection method remains an ongoing challenge. In our study, we conducted extensive dengue anomaly detection using various method and model specifications. A simulation study was implemented to test these methods under known ground truth situations, with scenarios designed to cover diverse disease space–time dynamics. Additionally, we conducted a case study using real monthly dengue national surveillance data in Thailand from 2018 to 2020.

Table 3 summarizes the key methods evaluated in this study for detecting space–time anomalies. The spatiotemporal Bayesian models, due to their flexibility and ability to account for space–time interactions, demonstrated superior performance in identifying complex clusters. FlexScan and SaTScan methods offered more computationally efficient options for detecting space–time clusters, although their performace varied depending on the scanning shape and cluster size. Local Getis and Moran methods, while effective for detecting smaller, localized clusters, were less suited for capturing broader space–time anomalies and exhibited lower overall accuracy in comparison to the other methods.

**Table 3:** Comparative summary of cluster detection methods.

Method	Strengths	Limitations
Spatiotemporal Bayesian	Accounts for complex dependencies across space and time; shows the highest performance	Requires greater computational resources and expertise
FlexScan	Computationally efficient; effective in quickly detecting irregular spatial patterns	Slightly less sensitive in identifying small or emerging clusters
Space–time SaTScan	Well-suited for detecting large-scale clusters over extended areas	Tends to identify larger clusters; reduced sensitivity for smaller or emerging clusters
Purely spatial SaTScan	Efficient for spatial cluster detection across larger regions	Primarily detects large clusters and does not account for temporal variation
Local Getis	Effective for detecting small, localized clusters	Lower sensitivity and specificity compared to other methods
Local Moran	Capable of detecting small-scale spatial clusters	Relatively low accuracy; limited to spatial data

Upon assessing various evaluation metrics, traditional methods such as Getis Ord and Moran’s I exhibited subpar performance, suggesting they might not be suitable for epidemiological disease cluster detection. Conversely, scanning-based approaches like spatial SaTScan had limitations, particularly in terms of positive predictive value, often identifying large clusters and leading to an overestimation of disease clustering, aligning with previous research^30^. On the other hand, FlexScan emerged as the top-performing method among testing-based approaches, while Bayesian-based models demonstrated superior performance. Interestingly, spatial Bayesian modeling with only a spatial component outperformed widely used testing-based cluster detection methods. It is important to note that the inclusion of a space–time interaction term in Bayesian models proved crucial, effectively absorbing spatiotemporal residuals from the data. Flexible forms of interaction, such as interaction type I, exhibited superiority in capturing space–time errors.

A detailed performance examination of SaTScan revealed intriguing details. Purely spatial SaTScan, employing circular and elliptical window shapes, faced challenges with specificity, especially at cluster boundaries, due to over-detection. This limitation may stem from the exclusive use of circular and elliptical cluster shapes, despite the inherent variability in disease transmission dynamics. The SaTScan method with space–time windowing functions encountered difficulties in detecting most spatiotemporal disease clusters. The space–time version demonstrated lower accuracy than purely spatial analyses, often identifying large clusters instead of pinpointing infectious transmission hotspots, which tend to be scattered and isolated. Similarly, Getis Ord and local Moran’s I underperformed, relying on overall averages or covariance instead of calculated expected values. The discrepancy in defining disease clusters emphasizes the need for caution when using these testing-based methods. According to the US CDC, disease clusters are typically described as an unusual grouping of health events in time and space reported to a health agency^31^. Differences in how disease clusters are defined may lead to less meaningful interpretations in the epidemiological field. Despite the widespread use of these testing-based methods in various epidemiological studies, our results suggest a need for caution. Conversely, FlexScan performed well in our study, showcasing flexibility in adapting to the changing dynamics of infectious diseases. Among testing-based methods, FlexScan emerges as a promising option.

While accuracy is a valuable summary measure for evaluating detection methods, it is important to also consider other key metrics that provide a more comprehensive view of model performance. In this study, the spatiotemporal Bayesian model stood out with strong performance across all evaluation metrics, making it the top performer. FlexScan also demonstrated a balanced trade-off between detection capability and computational efficiency, offering a practical option for real-time surveillance. However, SaTScan and local cluster detection methods, despite their faster computation times, exhibited notable limitations in terms of sensitivity and predictive values. Ultimately, the choice of model should be driven by the specific requirements of the application, whether prioritizing accuracy, sensitivity, computational speed, or a balance of these factors.

In our exploration of Bayesian methodology, experimentation with different model specifications revealed the critical importance of incorporating both spatial and temporal terms in Bayesian regression models to improve detection performance. Our findings underscore that models without these elements tend to fall short in capturing the complexity of spatiotemporal patterns essential for accurate disease detection. Moreover, the inclusion of space–time interaction terms emerged as a key factor in enhancing model performance, a conclusion supported by previous studies that emphasize the importance of these interactions in identifying and categorizing diseases based on specific space–time dynamics^32–34^.

Epidemiologically, dengue transmission, in particular, is driven by a combination of factors that often interact synergistically rather than acting independently. For example, environmental variables such as rainfall and temperature interact with socio-economic determinants like population density or healthcare access to create conditions conducive to dengue outbreaks. Main effects models that include only spatial and temporal random effects may not adequately account for such complex, interdependent relationships. In contrast, models with interaction terms can more effectively capture these complexities by reflecting how these variables jointly influence disease transmission rather than assuming their impacts are purely additive or independent.

We explored several forms of space–time interaction terms and found that the interaction type I, which involved only non-structured space–time components, exhibited the best overall performance in terms of both accuracy and computational efficiency. While models with more elaborate interaction structures might offer additional accuracy improvements, this must be weighed against the potential trade-offs. More complex models tend to demand substantially more computational resources, leading to longer processing times, which may only yield marginal performance improvements that do not justify the added complexity.

In the context of our study, the chosen model specification achieved a balance between reasonable accuracy and relatively short computational times, making it more feasible for real-time public health applications. However, increasing model complexity could result in excessive processing times, which would reduce the practical utility of these models for timely decision-making, particularly in outbreak settings where rapid detection and response are crucial. Thus, the trade-off between model complexity and computational efficiency should be carefully considered in future work, with the aim of achieving models that are both robust and operationally viable.

### Limitations and public health implications

While our study provides a comprehensive exploration of epidemiological behavior models, certain limitations should be acknowledged. The clustering threshold for Bayesian via was set as one, equivalent to the baseline level, with an exceedance probability of 0.95. Although these specifications have good public health and statistical meaning, varying these values could help determine the optimal setup. Other studies have explored baseline levels at 1.25 or 1.5, and exceedance probabilities ranging from 0.8 to 0.98, showing good performance in various settings^35,36^, including dengue cluster detection in Thailand^5,20,30^. Further investigation into varying values can contribute not only to space–time cluster detection but also to disease mapping in general. The real case study focused on dengue data in Thailand from 2018 through 2020. While our simulation study scenarios captured a range of complex epidemiological situations, additional studies with more diverse data applications and longer timeframes could improve our understanding and confirm the cluster detection behavior examined in this study. Such studies would contribute to validating and refining interpretations, aiding researchers in selecting suitable tools for spatiotemporal disease epidemiological clustering.

Our study provides valuable insights into the performance of cluster detection methods, offering the potential to enhance dengue surveillance. This information can empower public health agencies, particularly in endemic regions such as Thailand, to respond more effectively to outbreaks, optimize resource allocation, and improve public health outcomes. The adaptable nature of these methods allows for broader application in the surveillance of other infectious diseases with similar transmission patterns, providing a comprehensive framework for strengthening public health systems in response to emerging health threats. While our focus has been on dengue surveillance in Thailand, the results have broader implications, potentially facilitating data-driven public health policymaking at both local and broader scales.

## Conclusions

Dengue presents significant economic and public health challenges in many tropical countries, including Thailand. The effectiveness of disease surveillance is pivotal for healthcare policymakers to allocate resources strategically in disease control programs. However, determining the most effective space–time cluster detection method remains an ongoing challenge. In our study, we conducted extensive dengue anomaly detection using diverse methods and model specifications. Through a simulation study and a case study on national dengue surveillance in Thailand, we cautiously observed the limitations of widely used testing-based methods. Simultaneously, we found model-based spatiotemporal approaches to be more promising candidates. The versatility of these methods has the potential to extend their application, encompassing various infectious diseases with similar transmission patterns. Although our primary focus was on dengue surveillance in Thailand, the implications of our findings transcend, potentially benefiting public health policymaking at both local and broader scales. Consequently, authorities can make more informed decisions, strategically allocating limited resources and budgets to areas most in need.

## Electronic supplementary material

Below is the link to the electronic supplementary material.

Supplementary Material 1

## Data Availability

The datasets analyzed during the current study are available on the surveillance reporting system website, Bureau of Epidemiology, Department of Disease Control, Ministry of Public Health.
