# Supplementary material for: Comparative evaluation of spatiotemporal methods for effective dengue cluster detection with a case study of national surveillance data in Thailand
Source: Sci Rep. 2024 Dec 28;14:31064. doi: 10.1038/s41598-024-82212-1 (PMC11680836; doi:10.1038/s41598-024-82212-1)
Supplement: Supplementary file 1 — Supplementary Material 1 [file 41598_2024_82212_MOESM1_ESM.docx]

**Comparative evaluation of spatiotemporal methods for effective dengue cluster detection with a case study of national surveillance data in Thailand**

**Supplementary document S1: Spatiotemporal cluster detection methods and evaluation of spatiotemporal clustering procedures**

**S1.1 Spatiotemporal cluster detection methods**

***Anselin local Moran’s*** $\boldsymbol{I}$

The local Moran's I test or represented in this study as local Moran is the local level version of the global Moran's I test, estimated by dissecting the Moran's I statistic geographically, returning local components of the spatial association [1] which is one of the most popular spatial autocorrelation methods for areal data. To calculate this, on the study region $D$ contains the same meaning $y_{i}$ as before, Anselin local Moran’s $I$ can be calculated on the local area $i$ as $I_{i}$ as

$I_{i}=\frac{\Sigma_{j=1}^{N}w_{ij}\left( y_{i}-\bar{Y} \right)\left( y_{j}-\bar{Y} \right)}{\frac{1}{N}\Sigma_{j=1}^{N}\left( y_{j}-\bar{Y} \right)};\forall j\neq i$ (1)

where $\bar{Y}$ represents the global average over study space $D$ which consists of $N$ locations, and $w_{ij}$ denotes contiguity weight matrix. The local Moran is evaluated under the null hypothesis, assuming randomness in outcome values concerning the inspected one. A positive $I_{i}$ value implies that a feature shares similarity with its neighboring values, forming either a high or low cluster. Conversely, a negative $I_{i}$ value suggests that a feature has dissimilar values in its vicinity, identifying it as an outlier. This method categorizes each significant spatial unit into four types based on quadrant plots: 1) higher unit values with higher spatial lags (referred to as cluster HH), 2) lower node values with higher lags (referred to as outlier LH), 3) lower unit values with lower lags (referred to as cluster LL), and 4) higher node values with lower spatial lag values (referred to as outlier HL). Nevertheless, for any cluster or outlier to be considered statistically significant, the p-value associated with the feature must be smaller than a pre-specified level of significance.

***Getis Ord G_i_****

Getis-Ord G_i_* is another local clustering detection method designed for aggregated data, specifically employed to compare local estimates of spatial autocorrelation with global averages, facilitating the identification of clusters in spatial data [2]. Given a study region *D* with disease outcomes represented as $y_{i}$ at each location *i*, the calculation of Getis-Ord G_i_*, which emphasizes the comparison of the focal area with its neighbors [3], can be expressed as follows

$G_{i}^{*}=\frac{\Sigma_{j=1}^{N}\left( w_{ij}y_{j} \right)-\left( \frac{1}{N}\sum_{j=1}^{N} y_{j} \right)\left( \sum_{j=1}^{N} w_{ij} \right)}{S\sqrt{\frac{\left( N\sum_{j=1}^{N} w_{ij}^{2} \right)-\left( \sum_{j=1}^{N} w_{ij} \right)^{2}}{N-1}}};\forall j$ (2)

where $N$ is the number of areal nodes within space $D$, $w_{ij}$ denotes values in adjacency matrix, and $S$ is standard deviation of all values $y_{i}$. Spatial units exhibiting significantly high *G_i_** values are recognized as hotspot clusters, whereas locations with low *G_i_** values are conversely characterized as cold spot clusters. This calculation is based on hypothesis testing under the null hypothesis of spatial independence, with z-scores and p-values obtained from permutation testing.

***Spatial and Space-Time Scan Statistics (SaTScan)***

Let $D$ represent the spatial space containing a set of continental centroids within this region, denoted as $A\subset D$. Under the Poisson model, there is only one available scanning zone $Z\subset D$ such that $N_{A}\sim Poisson\left( p\mu_{A\cap Z}+q\mu_{A\cap Z^{C}} \right) \forall A$ where $N_{A}$ is the number of points in the set $A$, $\mu$ is population average in space $D$, $p$ and $q$ are, respectively, probability of any points in the set $A$ that intersect inside and outside interest zone $Z$ [4]. The procedure relies on the null hypothesis $H_{0}:p=q$ or $N_{A}\sim Poisson\left( p\mu_{A} \right)$ indicating the insignificance of the interest window $Z$. The alternative hypothesis of the procedure is parallelly defined as $H_{a}:p>q$.

The scanning zone algorithm for *Z* begins with a representative point localization process. Initially, inspection windows are randomly placed, and the area of circular windows Z (or elliptical appearance, based on user settings) is expanded until reaching the limits. The likelihood form of the Poisson model, adapted for selecting significant windows, is proportionally expressed as $\left( \frac{N_{Z}}{\mu_{A}} \right)^{N_{Z}}\left( \frac{N_{A}-N_{Z}}{N_{A}-\mu_{A}} \right)^{N_{A}-N_{Z}}I(N_{Z}>\mu_{A})$ where $N_{Z}$ represents the number of cases appearing inside the interest window $Z$ and the function $I$ serves as an indicator, equaling 1 if $N_{Z}>\mu_{A}$, otherwise defined as zero (the indicator function is constructed in reverse for the purpose of identifying low-value anomalies). For nodes likely to be included in multiple inspected windows, the interested nodes are primarily induced into a window *Z* containing nearest neighbor relations [5]. Furthermore, the maximization of the likelihood ratio is necessary to declare the most likely clusters in terms of window extent.

Because of inexistence of closed-form distribution for the spatial scan statistic, nonparametric-based p-value approximation such as Monte Carlo hypothesis testing is traditionally instead applied [6]. Randomized data were initially simulated with real input data but with different spatial localizations. Subsequently, the spatial scan statistic was repeatedly tested on these randomized datasets, and the proportion was calculated between the number of randomized datasets for which the likelihood ratios were higher than the previous statistic from the true real data, divided by the total number of replications, *n*, denoted as $\alpha=\frac{\Pr\left( L_{rand}>L_{data} \right)}{n}$.

While spatial scan statistics enable the identification of clusters of cases, defined as sets of points or regions with either high or low incidence rates at a user-defined granularity. The space-time scan statistic employs a cylindrical scanning window, where the circular base captures the spatial dimension and the height represents a temporal interval [7, 8]. To detect space-time clusters at the county level, the center of the circular base aligns with the centroid of each spatial unit. As the scan proceeds, the radius of the circular base and the height of the cylinder vary from lower bounds to spatial and temporal upper limits. As each cylinder traverses the study area, it encompasses different sets of cases over different time intervals, potentially identifying emerging space-time cluster candidates. The likelihood ratio test statistic for space-time scan statistics is calculated similarly to the purely spatial scan statistic [8], with the significance level derived from both purely spatial and space-time scan statistics, allowing for comparison with a predefined significance level.

***Flexible Scan Statistics (FlexScan)***

Flexible scan statistics share some computational background with SaTScan but differ in the shape of scanning windows. FlexScan, introduced briefly here and with more details available in [5, 9], is designed for detecting and evaluating purely spatial disease clusters, similar to SaTScan. However, unlike SaTScan, FlexScan employs a non-parametric definition of clusters rather than circular or elliptical shapes, although it also includes circular windows as a special case. Irregularly shaped windows Z are constructed on any nodes in spatial space D by connecting adjacent regions [9]. Sets of flexibility windows are composed with a length k that consists of k connected regions, including the self-node. The amplitude of connected regions is adjusted between 1 and a maximum predefined number K to maximize the likelihood ratio. Nearest neighbors' relations are used to determine which nodes belong to the most appropriate window Z. Results from flexible scan statistics comprise circular window regions and sets of connected regions [5] indicating that the results are more diverse than those from purely circular scan statistics. Note that for the spatial testing-based methods that do not have a temporal component, we applied these methods iteratively at each time point.

***Spatiotemporal Bayesian modeling with exceedance probability***

Bayesian statistical regression models have shown to be effective in analyzing epidemiological data with spatial and spatiotemporal characteristics [10, 11]. A key advantage lies in their ability to consider uncertainty in estimates with the integration of spatial and temporal structures as prior distributions [12]. Moreover, this approach accommodates a broader range of conceptual models compared to non-Bayesian methods [13]. However, there are various configurations on prior distributions and likelihood functions for different aspects, along with statistical inferencing procedures which were described in the following.

Let $y_{it}$ represent the case count at time $t$ for any location $i$ . This can be formally expressed as Poisson distributed as [14]

$y_{it}\sim Poisson\left( E_{it}\theta_{it} \right)$

(3)

where $b_{0}$ is the overall intercept term, $E_{it}$ is expected values and $\theta_{it}$ denotes relative risk of disease in the area $i$ and time period *t*. $s_{i}$ represent the spatial random effect, while $\gamma_{t}$ and $\omega_{it}$ are temporal and space-time interaction terms respectively. The random effects can be linked to the $\theta_{it}$ by a logarithm link function. In general, it is important to include both structured and unstructured random effects in spatial and spatiotemporal analyses because confounding can take various forms with both spatial and non-spatial structures. The unstructured random effect, denoted by $v_{i}$, is often described by a Gaussian prior distribution with zero mean and variance $\sigma_{v}^{2}$. One traditional model of structural random effect, normally defined as $u_{i}$, is formed as intrinsic conditional autoregressive model (BESAG) distribution [15]. This model has a conditional form of $u_{i}|u_{-i}\sim N\left(\bar{u}_{\Omega_{i}},\sigma_{u}^{2}/n_{\delta_{i}}\right)$ where $u_{-i}$ is the vector containing the correlated effect of all except the *i* th area. $\Omega_{i}$, $n_{\delta_{i}}$ and $\bar{u}_{\delta_{i}}$ are a set of the first-order spatial neighbors, cardinality and the average of the neighborhood of the *i* th area respectively, and$\sigma_{u}^{2}$ is the variance of the spatial term.

To capture the temporal patterns in dengue case patterns, various forms of temporal random effects can be adopted. However, in this study our focus was on non-focus clustering, which could have flexible forms. A reasonable structure of temporal term is a nonparametric random walk prior model. In general, a random walk is assumed to have a prior Gaussian distribution with a mean as the previous time point, which can be either positive or negative. In this study, we applied two models of random walks: a random walk of order 1 (RW1) and a random walk of order 2 (RW2). The RW1 prior can be expressed as $\gamma_{t}|\gamma_{t-1}\sim N(\gamma_{t-1},\sigma_{\gamma}^{2} )$

and RW of order 2 can be formulated as $\gamma_{t}|\gamma_{t-1},\gamma_{it-2}\sim N\left( 2\gamma_{t-1}+\gamma_{t-2},\sigma_{\gamma}^{2} \right)$. Note that we also assessed the performance of purely spatial and temporal models in this space-time application. However, to utilize these Bayesian models, we applied these methods iteratively at each spatial and temporal unit.

The specification of interaction term,, depends on the spatial and temporal random effects assumed to interact in the model. Various types of interactions have been proposed in the Bayesian disease mapping literature [16]. However, for the purposes of this study, we employed the commonly used type I interaction [17]. Under this interaction specification (), the random effect was assumed to be interaction between the non-spatial, $v_{i}$, and exchangeable Gaussian temporal, $\delta_{t}$, terms. According to Knorr-Held notation [16], the structure matrix$R_{\theta}$for the prior ofcan be expressed as the Kronecker product of the interacting random effects. For the first type of interaction, the structure matrix can be written assince both $v_{i}$ and$\delta_{t}$ do not have a specific spatiotemporal structure. Note that ***I*** here is the identity matrix.

To identify anomalies, we utilized exceedance probabilities as $\Pr\left( \theta_{i}>q \right)$ where *q* represents the threshold. When *q* = 1, the threshold was set as the expected rate or baseline. In this study, a hotspot was characterized as a location where $\Pr\left( \theta_{i}>q \right)$ exceeded a specified cut-off point, i.e., $\Pr\left( \theta_{i}>q \right)>1-\alpha$ where $\alpha$ was predetermined level of significance. Estimates derived from the posterior predictive distribution can be computed from converged posterior samples using sampling-based algorithms such as Markov Chain Monte Carlo (MCMC). However, in real-time infectious disease surveillance, timeliness is crucial. With a multidimensional model setup and accumulating surveillance data over time, the parameter space can rapidly expand, demanding exponential computational resources. An efficient alternative for parameter inference in this context is the Integrated Nested Laplace Approximation (INLA) [18]. Utilizing optimized numerical routines for these computations and the compatibility of our proposed model with the INLA format, we implemented the model using the numerical Laplace approximation within the R-INLA package, accessible at www.r-inla.org.

**S1.2 Evaluation of spatiotemporal clustering procedures**

Infectious disease surveillance systems must strike a balance between accurately detecting outbreaks and allocating disease control resources effectively. Therefore, the concepts of optimal criteria, including accuracy, sensitivity, specificity, positive predictive value (PPV), and negative predictive value (NPV), are crucial for comparing and assessing the validity of cluster detection methods. These five evaluation metrics were utilized in this simulation study to compare and evaluate the performance of different methods. In the calculations, TP represents true positives, FP represents false positives, FN represents false negatives, and TN represents true negatives. Accuracy, defined as the sum of true positives and true negatives divided by the total count, was computed as (TP+TN)/(TP+FP+FN+TN). Sensitivity, indicating the probability of a positive test given the presence of hotspots, was calculated as TP/(TP+FN), while specificity, representing the proportion of negative tests among non-hotspots, was determined as TN/(FP+TN). Sensitivity and specificity are commonly used to evaluate a test's ability to correctly identify or rule out the ground truth condition [19]. Conversely, PPV and NPV provide the probability of hotspot appearance or absence based on test results [20]. PPV was computed as TP/(TP+FP), and NPV was calculated as TN/(FN+TN). These indicators were spatially visualized at the provincial level to facilitate comparison and discussion of the models' performance.

**Supplementary document S2: Results from simulation study**

Note that the spatiotemporal Bayesian model presented here utilizes the best-performing configuration, incorporating random effects of Besag spatial effect, RW2 temporal effect, and Type I interaction terms.

*Spatiotemporal Bayesian model*
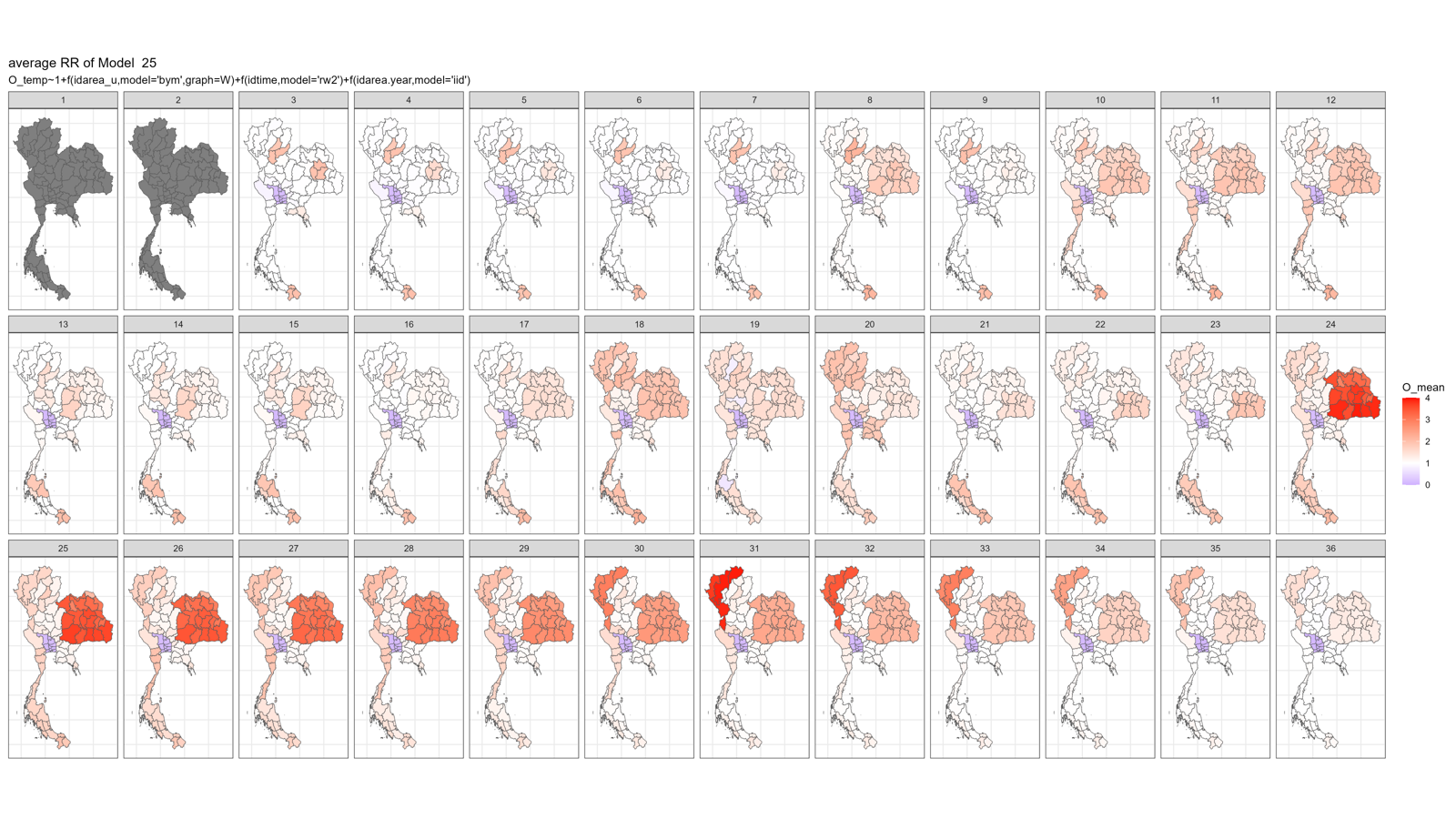


Relative Risk maps of the spatiotemporal Bayesian model, generated using RStudio version 2022.07.0+548 (available at https://posit.co/products/open-source/rstudio/).


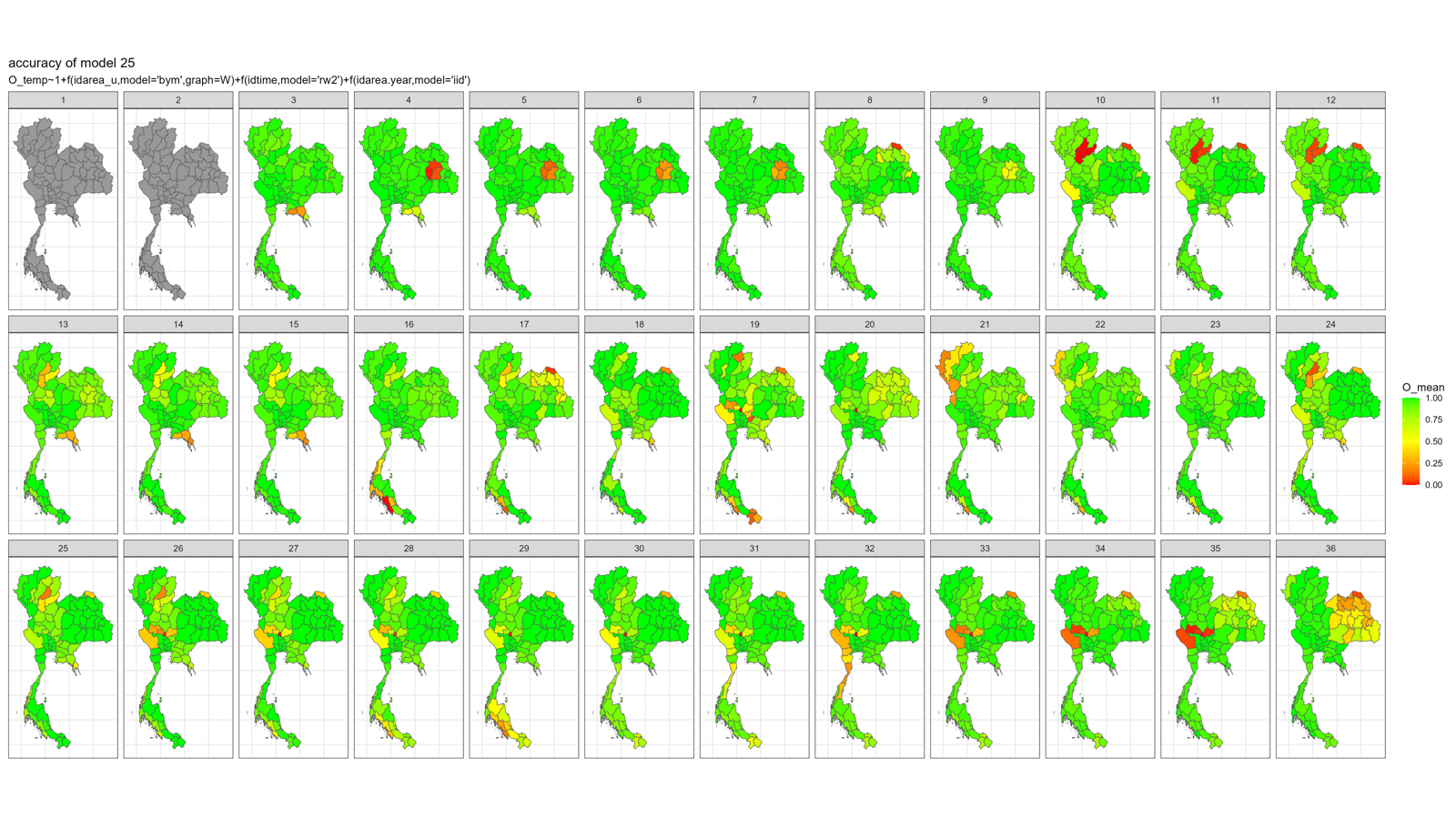


Accuracy maps of the spatiotemporal Bayesian model, generated using RStudio version 2022.07.0+548 (available at https://posit.co/products/open-source/rstudio/).


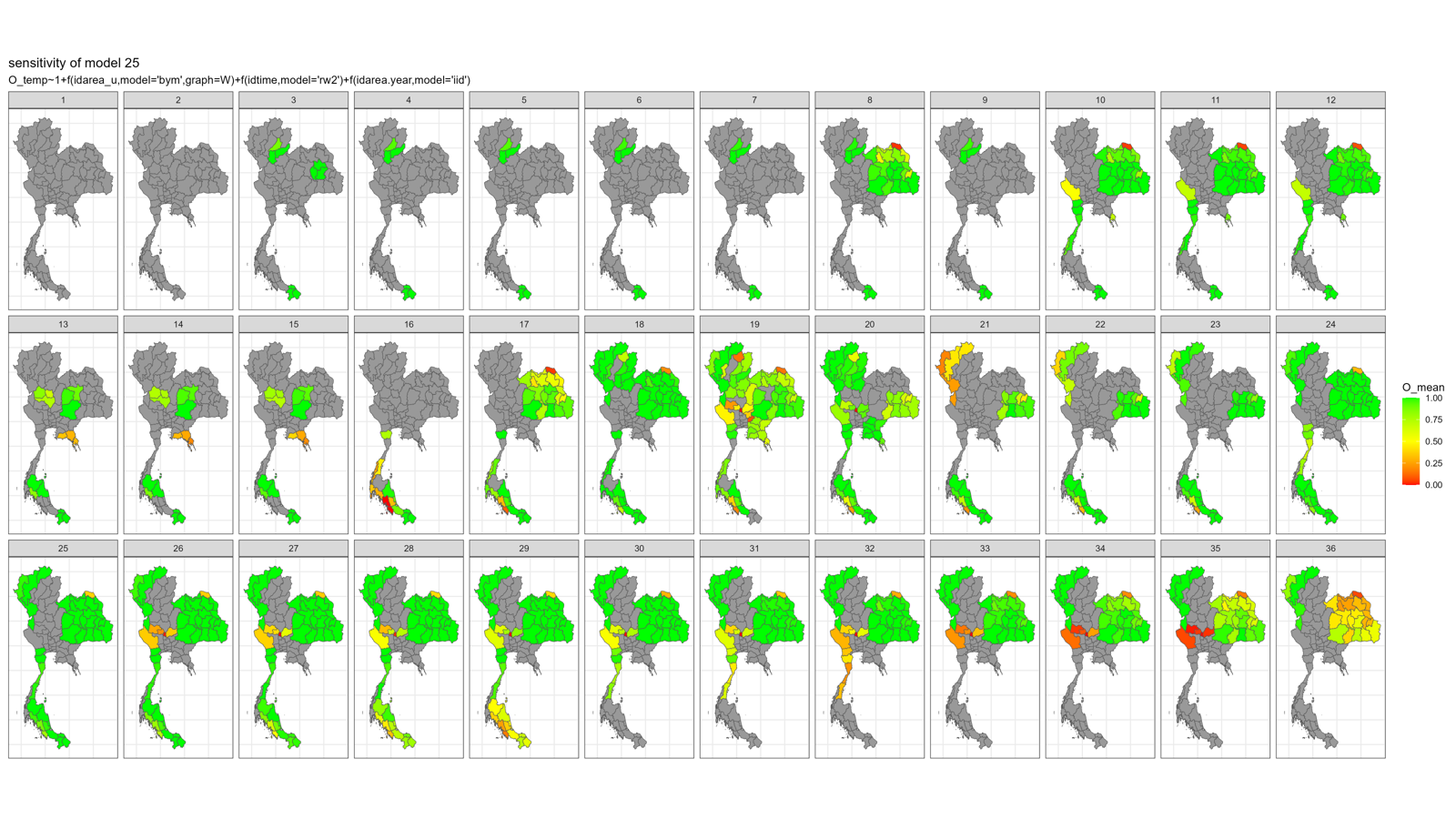


Sensitivity maps of the spatiotemporal Bayesian model, generated using RStudio version 2022.07.0+548 (available at https://posit.co/products/open-source/rstudio/).


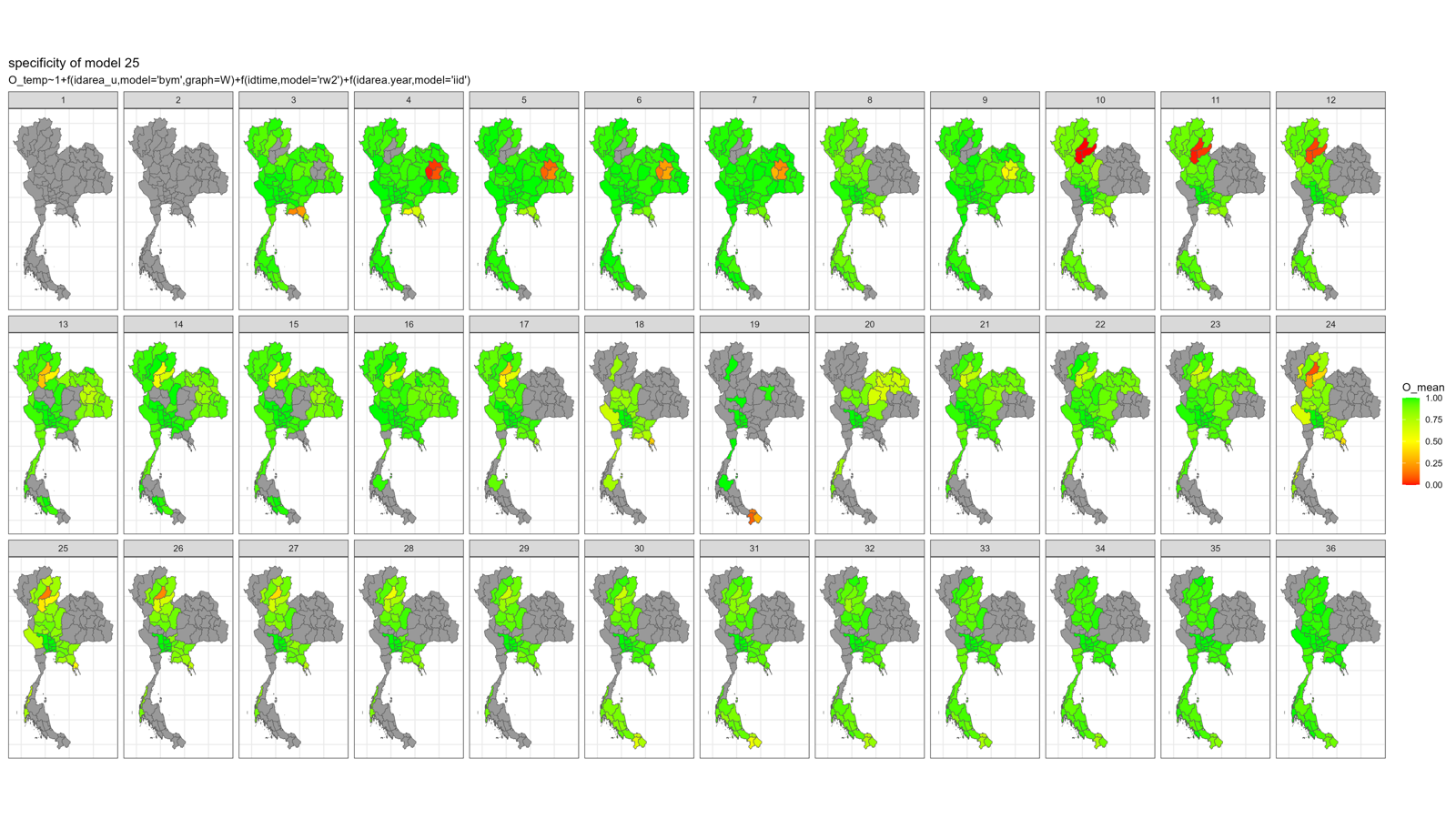


Specificity maps of the spatiotemporal Bayesian model, generated using RStudio version 2022.07.0+548 (available at https://posit.co/products/open-source/rstudio/).


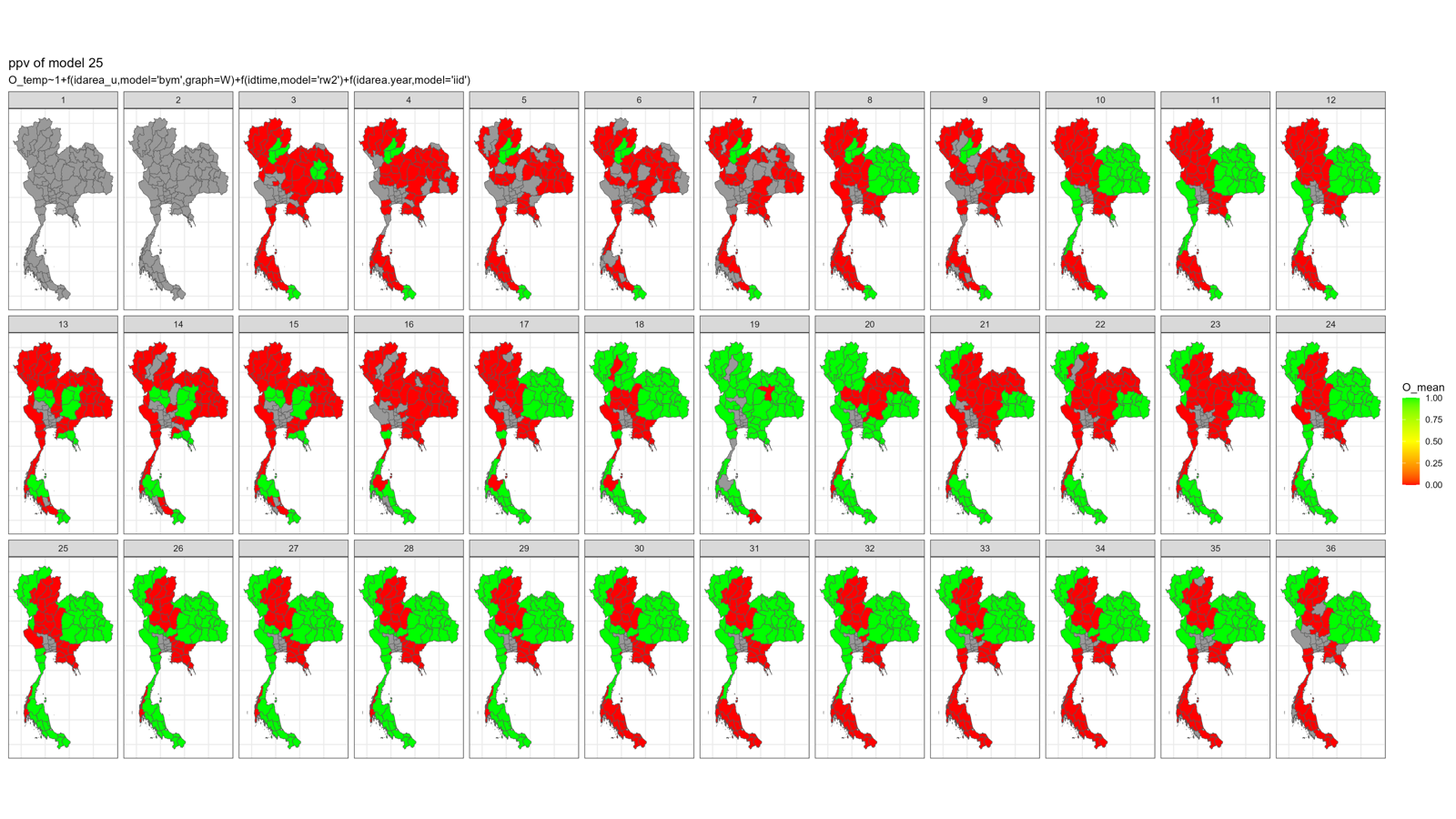


PPV maps of the spatiotemporal Bayesian model, generated using RStudio version 2022.07.0+548 (available at https://posit.co/products/open-source/rstudio/).


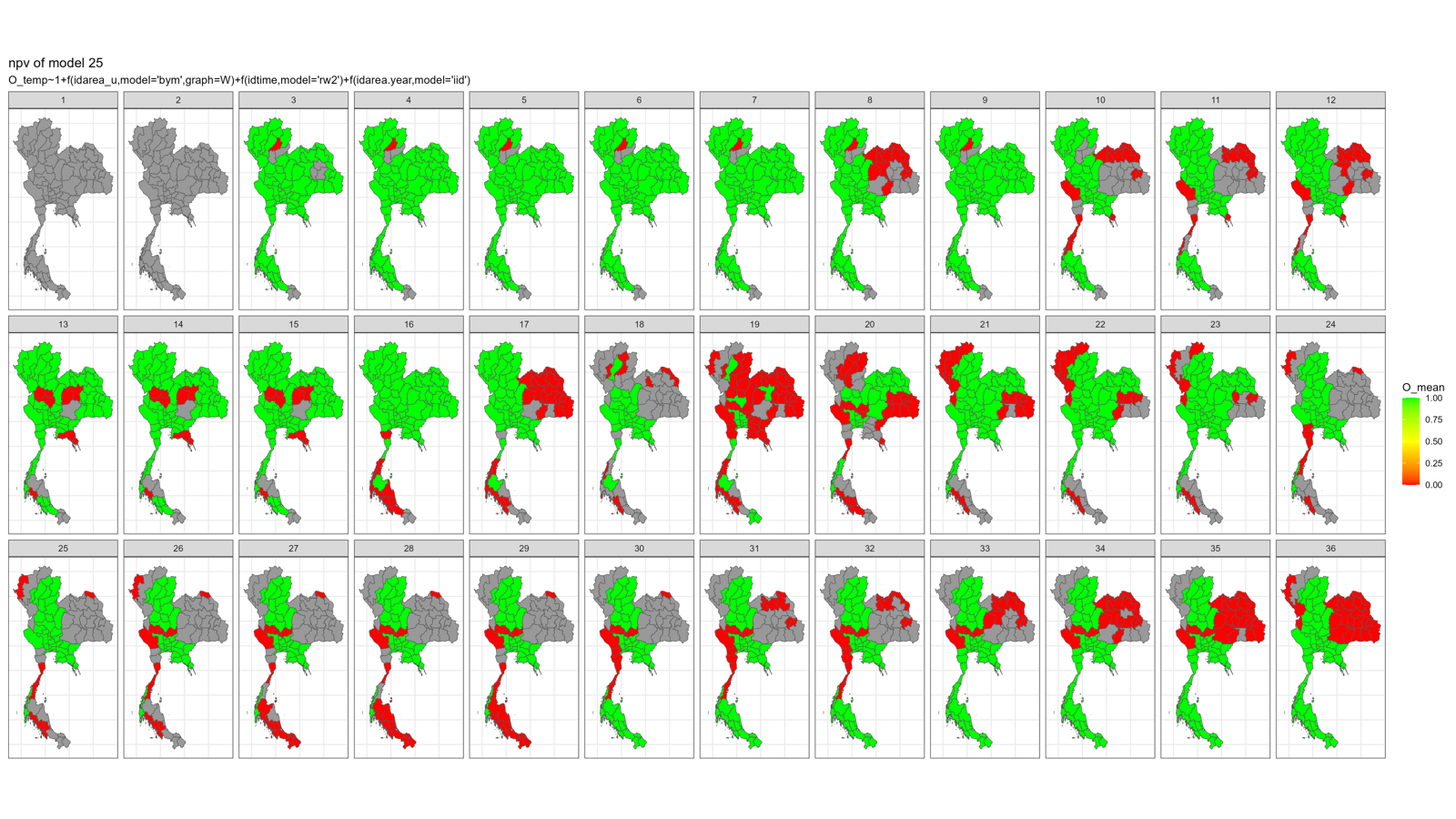


NPV maps of the spatiotemporal Bayesian model, generated using RStudio version 2022.07.0+548 (available at https://posit.co/products/open-source/rstudio/).

*Flexscan with circular scanning window*


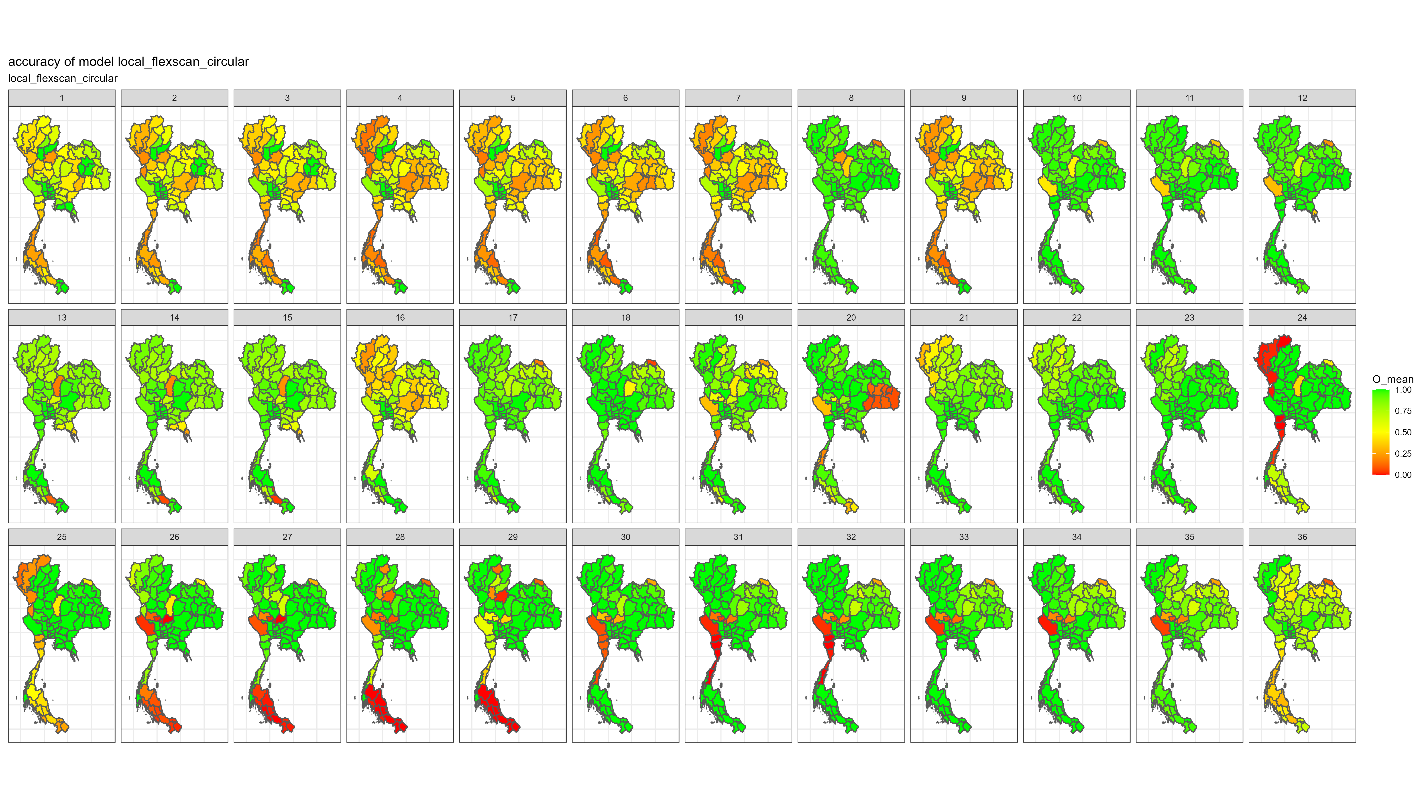


Accuracy maps of Flexscan model, circular windowing function, generated using RStudio version 2022.07.0+548 (available at https://posit.co/products/open-source/rstudio/).


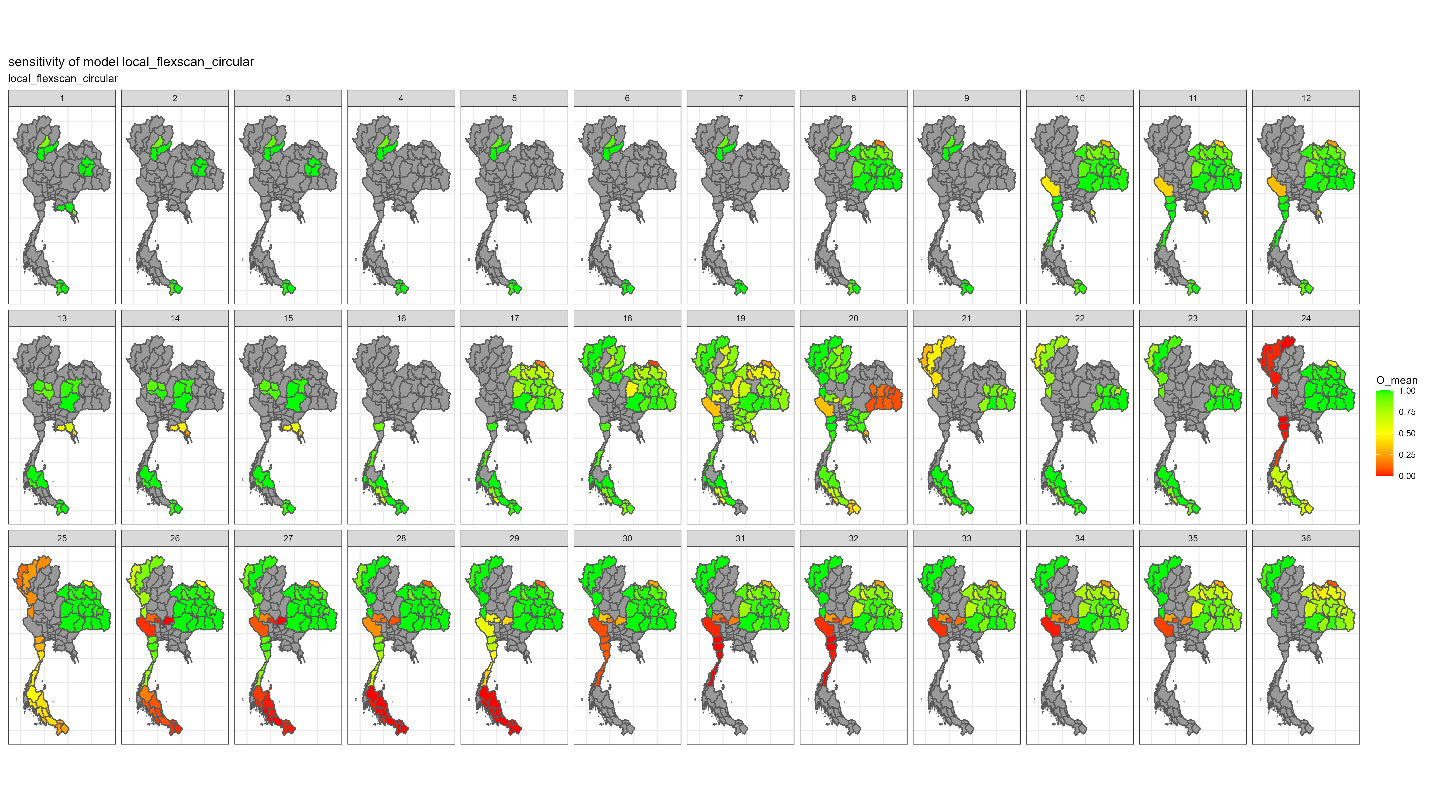


Sensitivity maps of Flexscan model, circular windowing function, generated using RStudio version 2022.07.0+548 (available at https://posit.co/products/open-source/rstudio/).


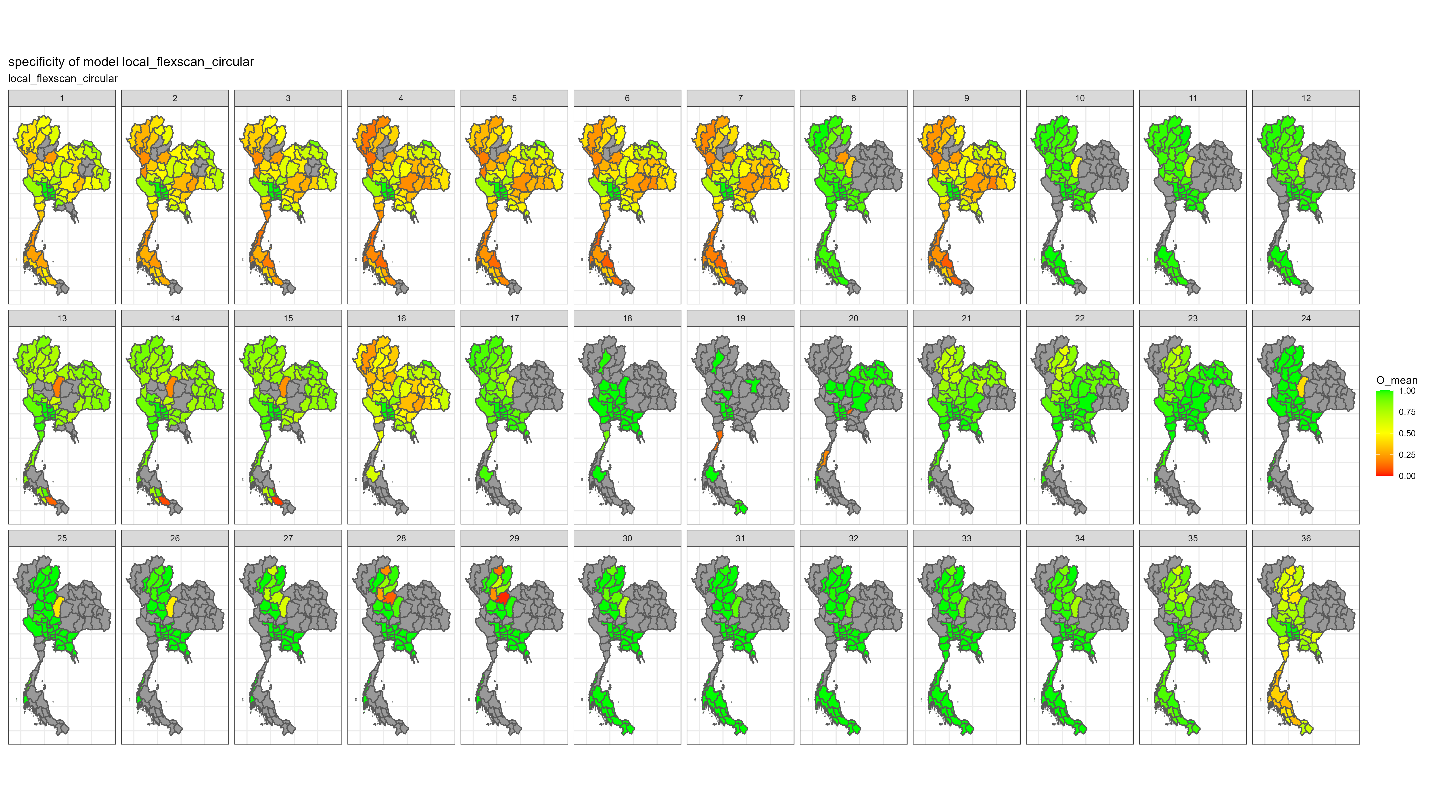


Specificity maps of Flexscan model, circular windowing function, generated using RStudio version 2022.07.0+548 (available at https://posit.co/products/open-source/rstudio/).


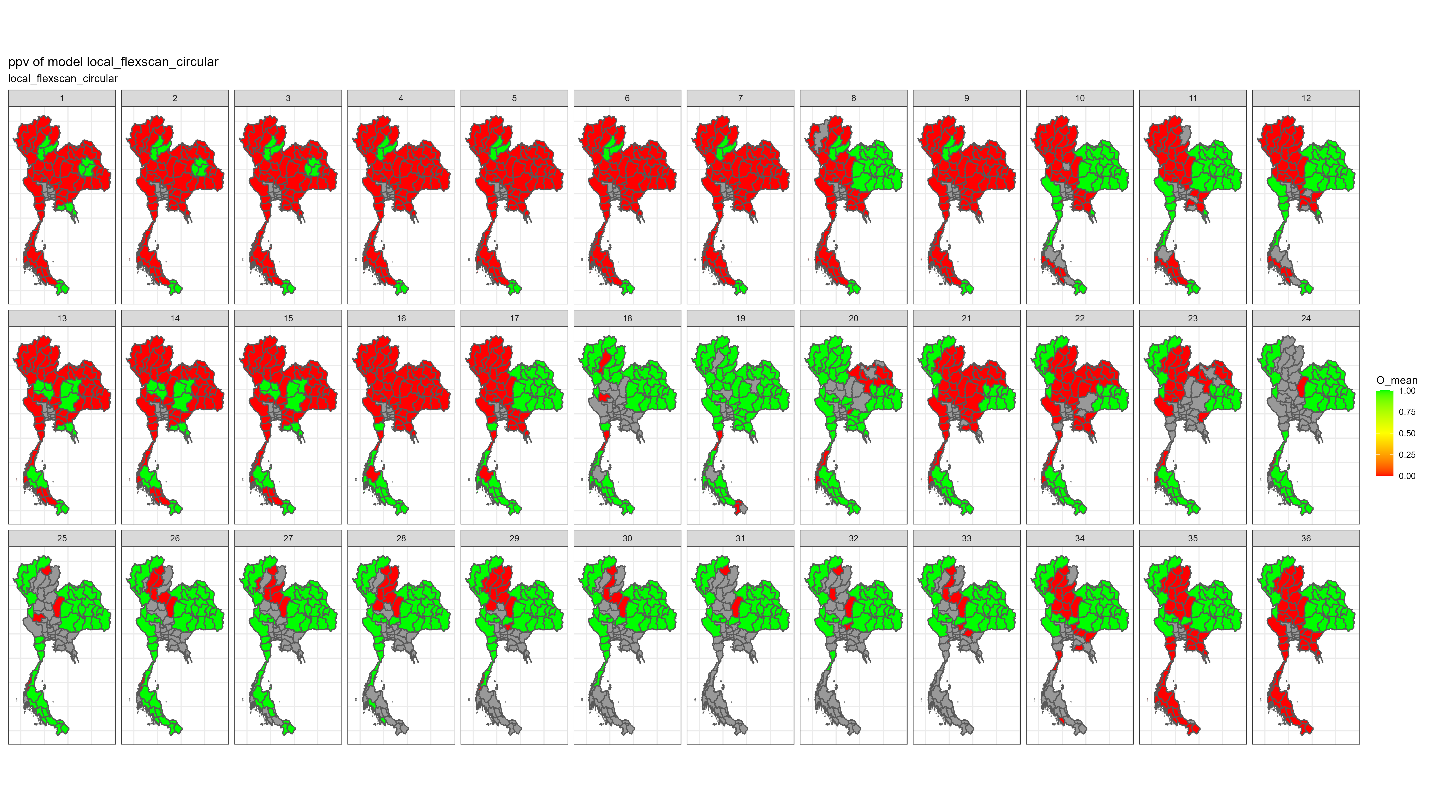


PPV maps of Flexscan model, circular windowing function, generated using RStudio version 2022.07.0+548 (available at https://posit.co/products/open-source/rstudio/).


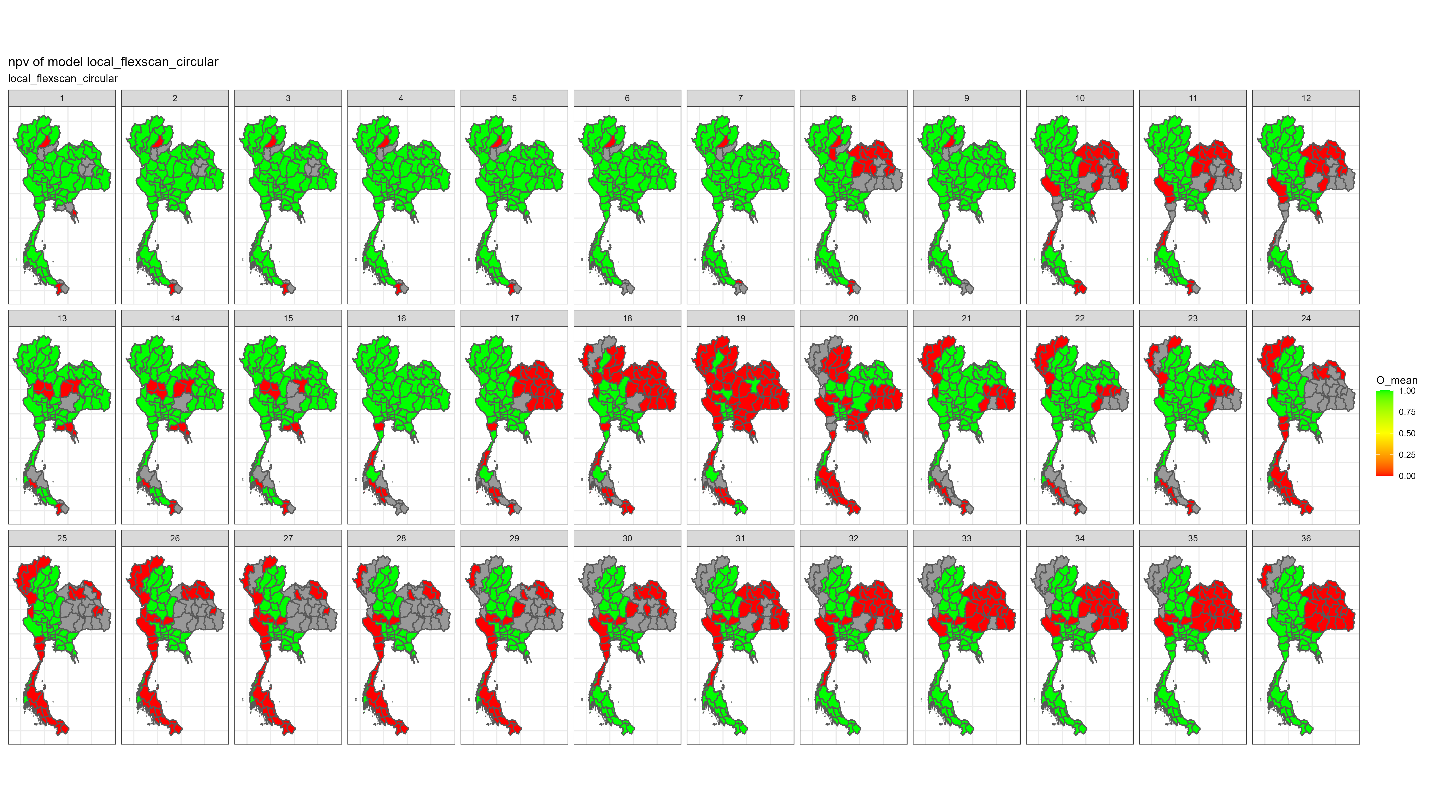


NPV maps of Flexscan model, circular windowing function, generated using RStudio version 2022.07.0+548 (available at https://posit.co/products/open-source/rstudio/).

*Flexscan with flexible scanning window*


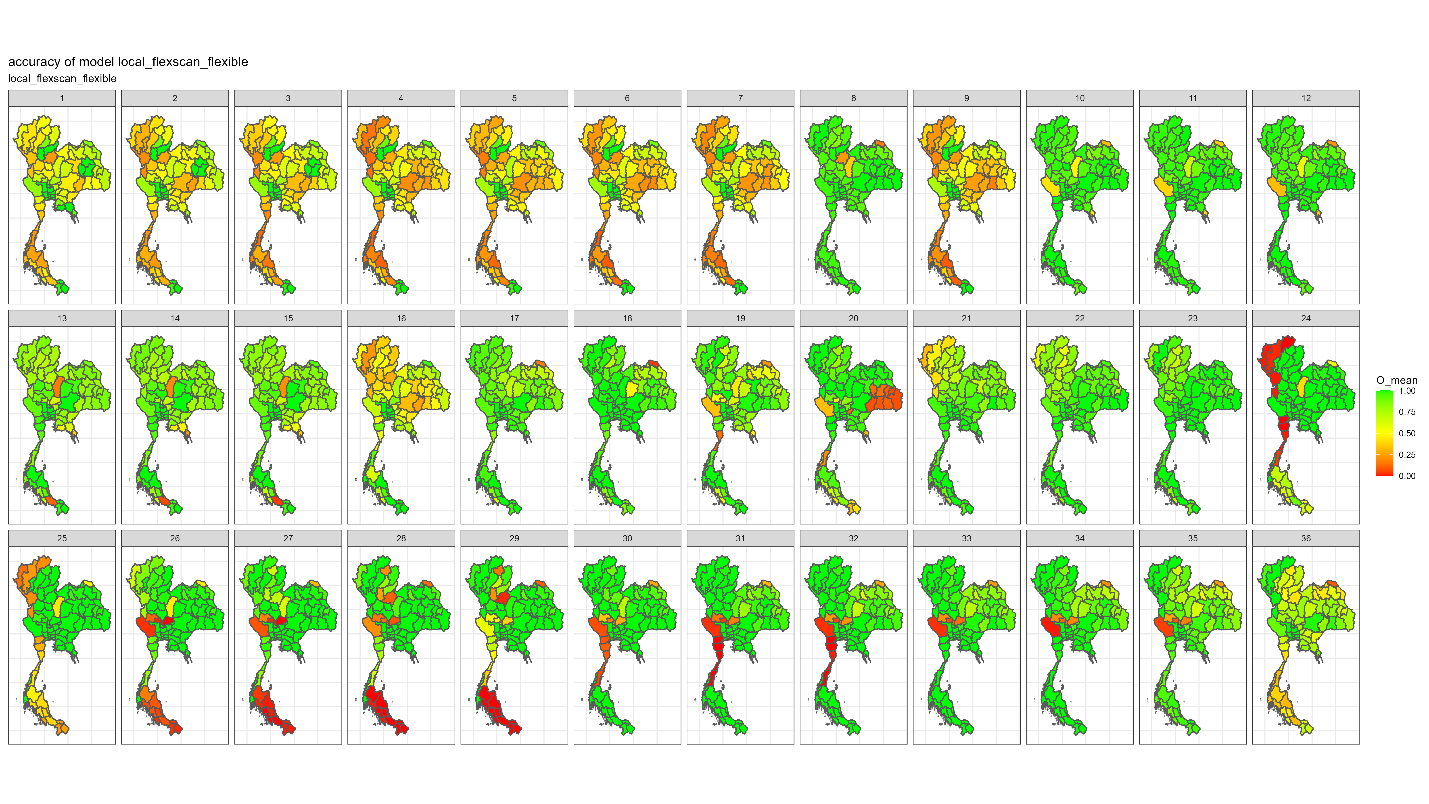


Accuracy maps of Flexscan model, flexible windowing function, generated using RStudio version 2022.07.0+548 (available at https://posit.co/products/open-source/rstudio/).


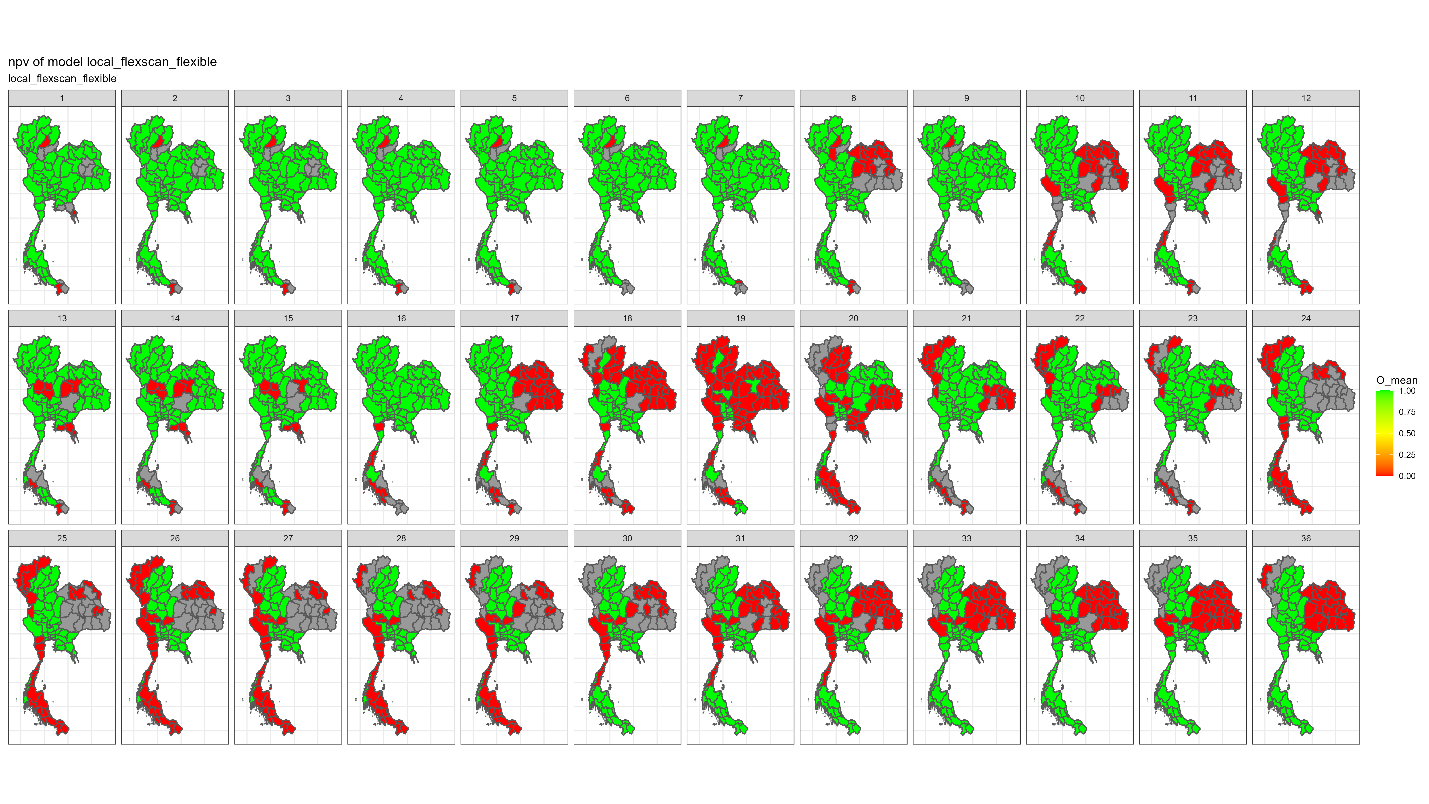


NPV maps of Flexscan model, flexible windowing function, generated using RStudio version 2022.07.0+548 (available at https://posit.co/products/open-source/rstudio/).


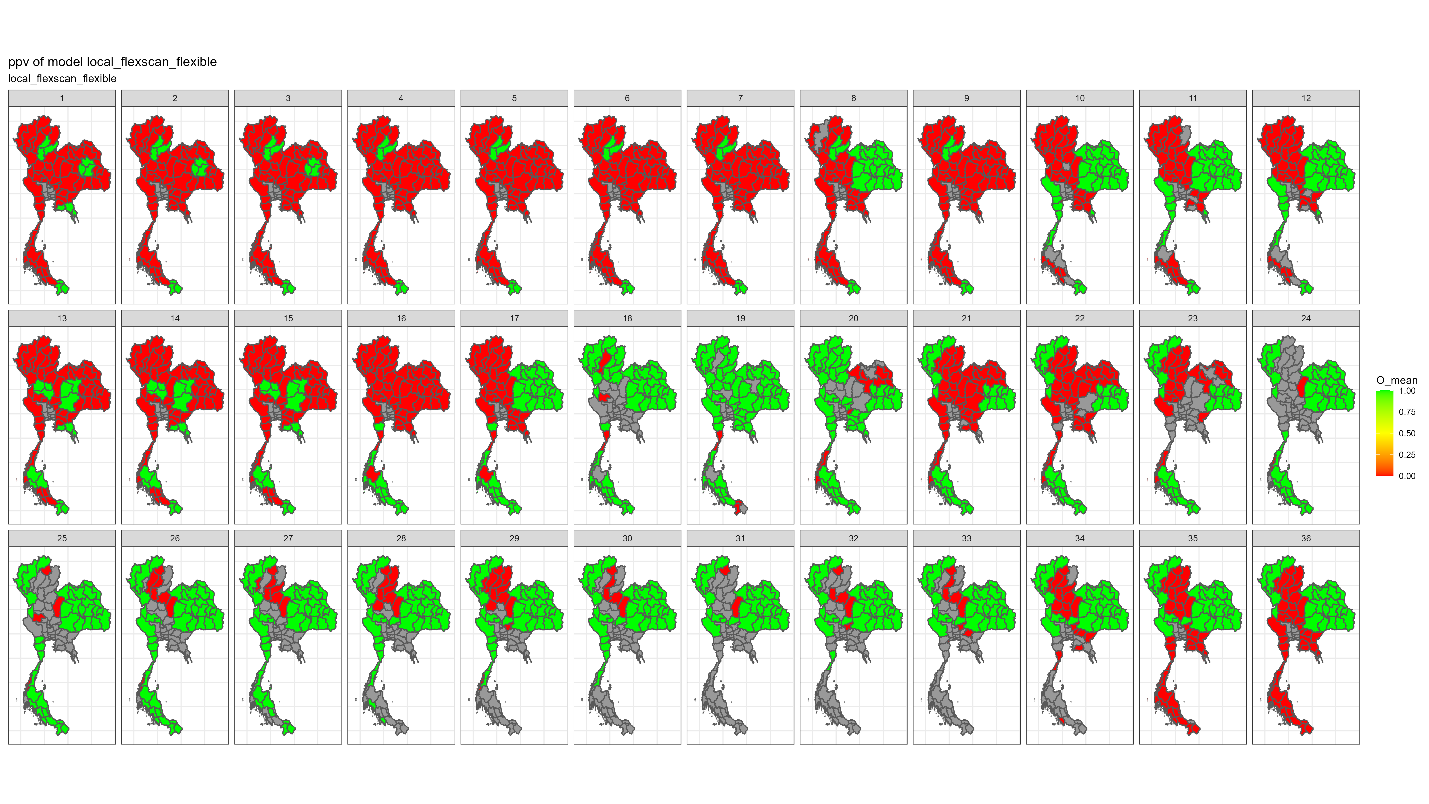


PPV maps of Flexscan model, flexible windowing function, generated using RStudio version 2022.07.0+548 (available at https://posit.co/products/open-source/rstudio/).


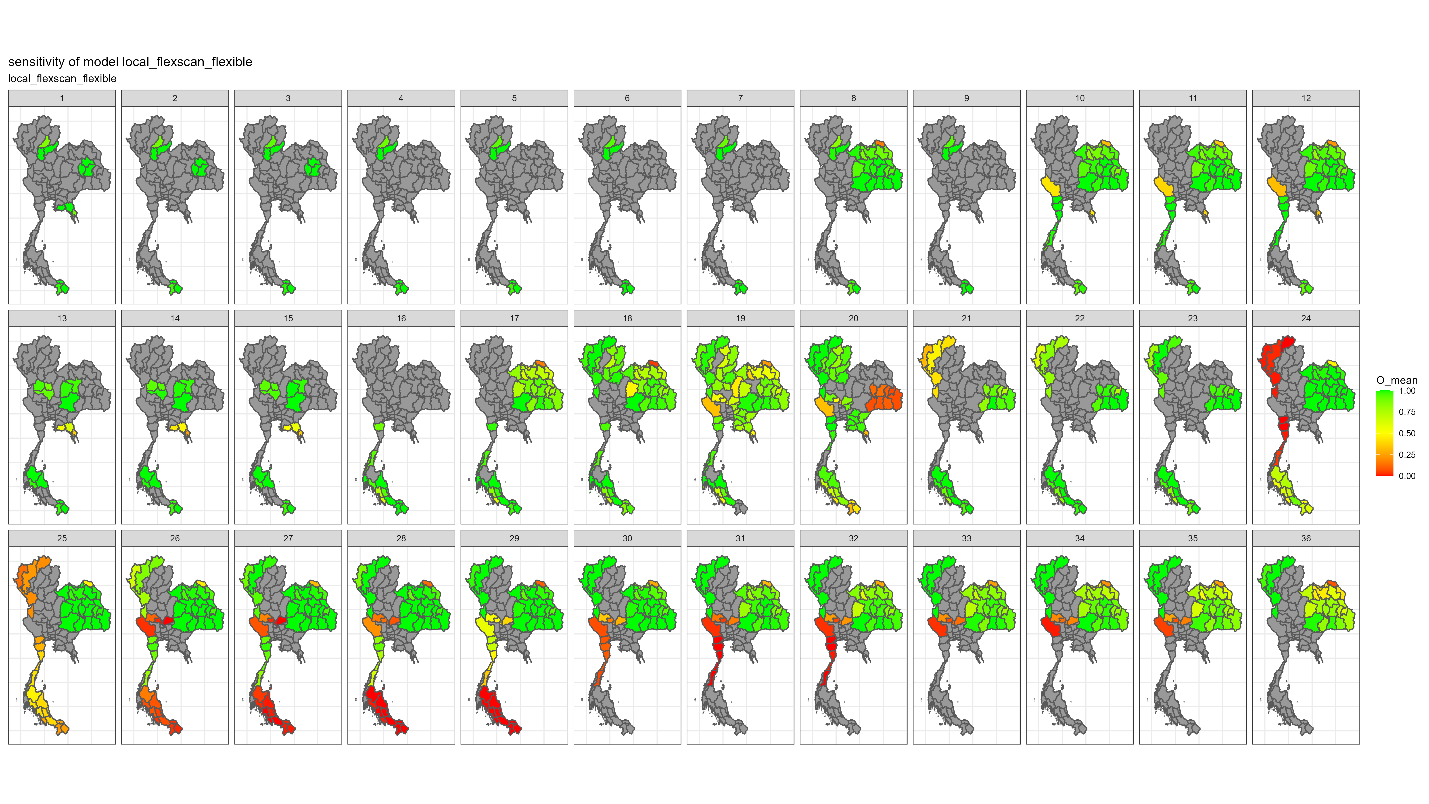


Sensitivity maps of Flexscan model, flexible windowing function, generated using RStudio version 2022.07.0+548 (available at https://posit.co/products/open-source/rstudio/).


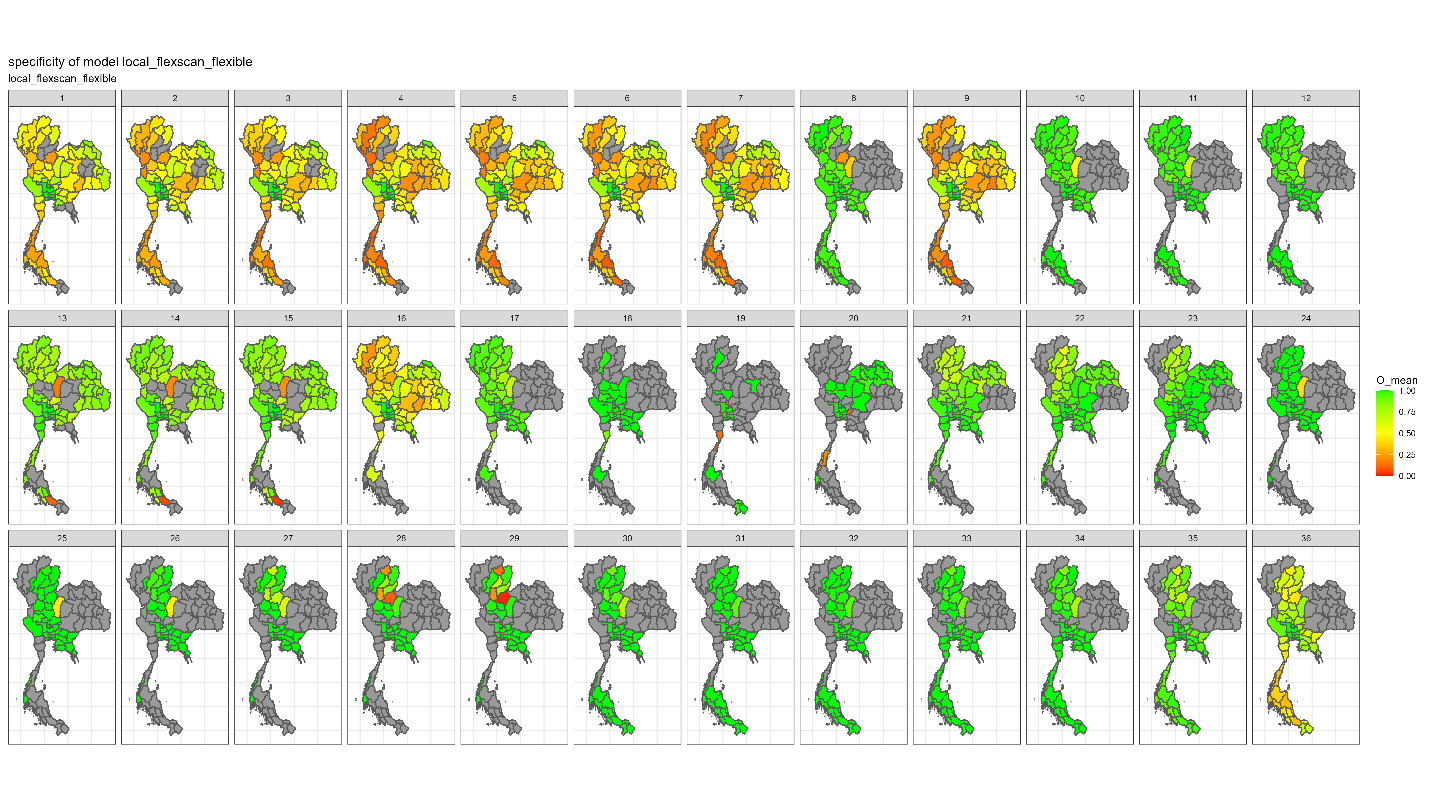


Specificity maps of Flexscan model, flexible windowing function, , generated using RStudio version 2022.07.0+548 (available at https://posit.co/products/open-source/rstudio/).

*Space-time Satscan with elliptic scanning window (retrospective)*


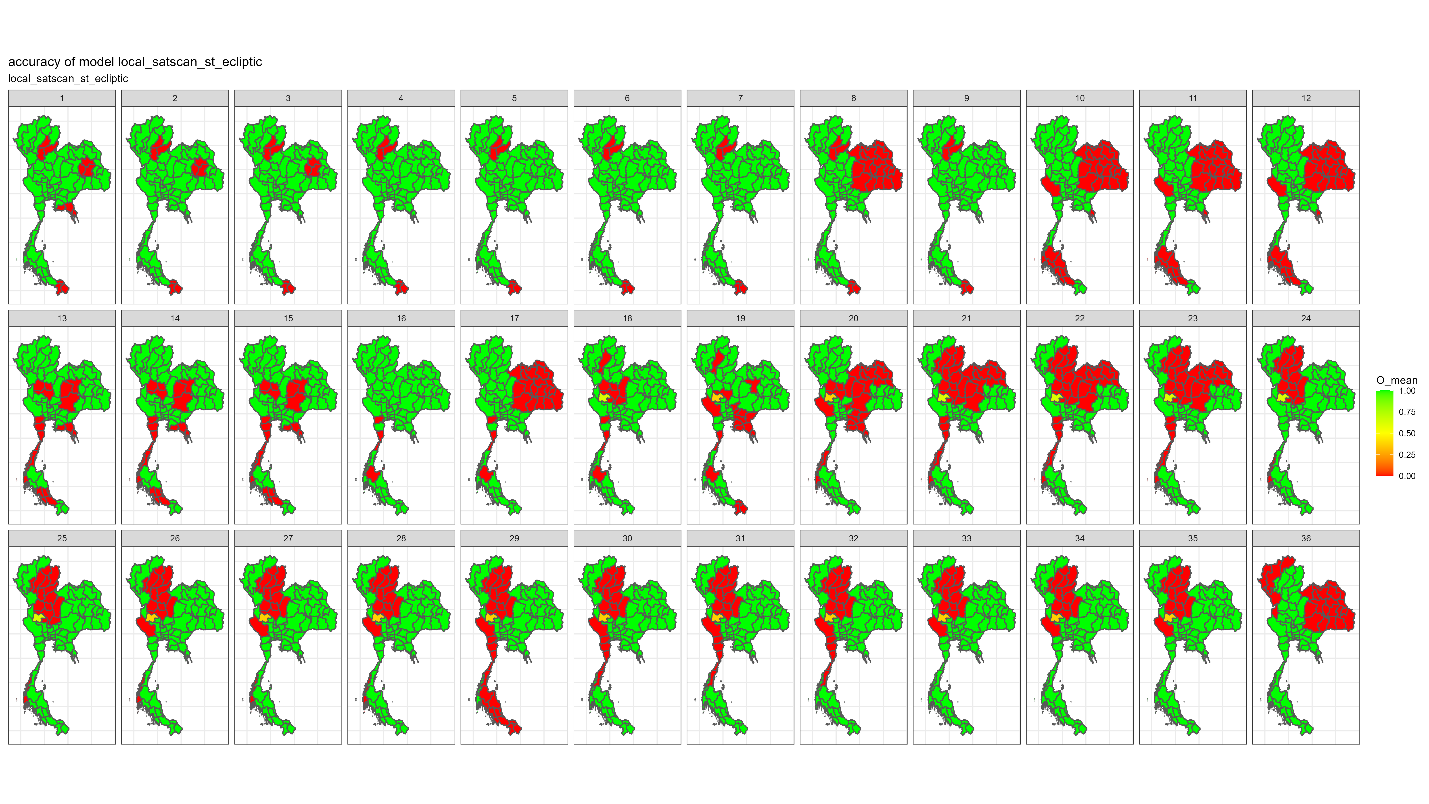


Accuracy maps of retrospective ST SaTScan, elliptical windowing function, generated using RStudio version 2022.07.0+548 (available at https://posit.co/products/open-source/rstudio/).


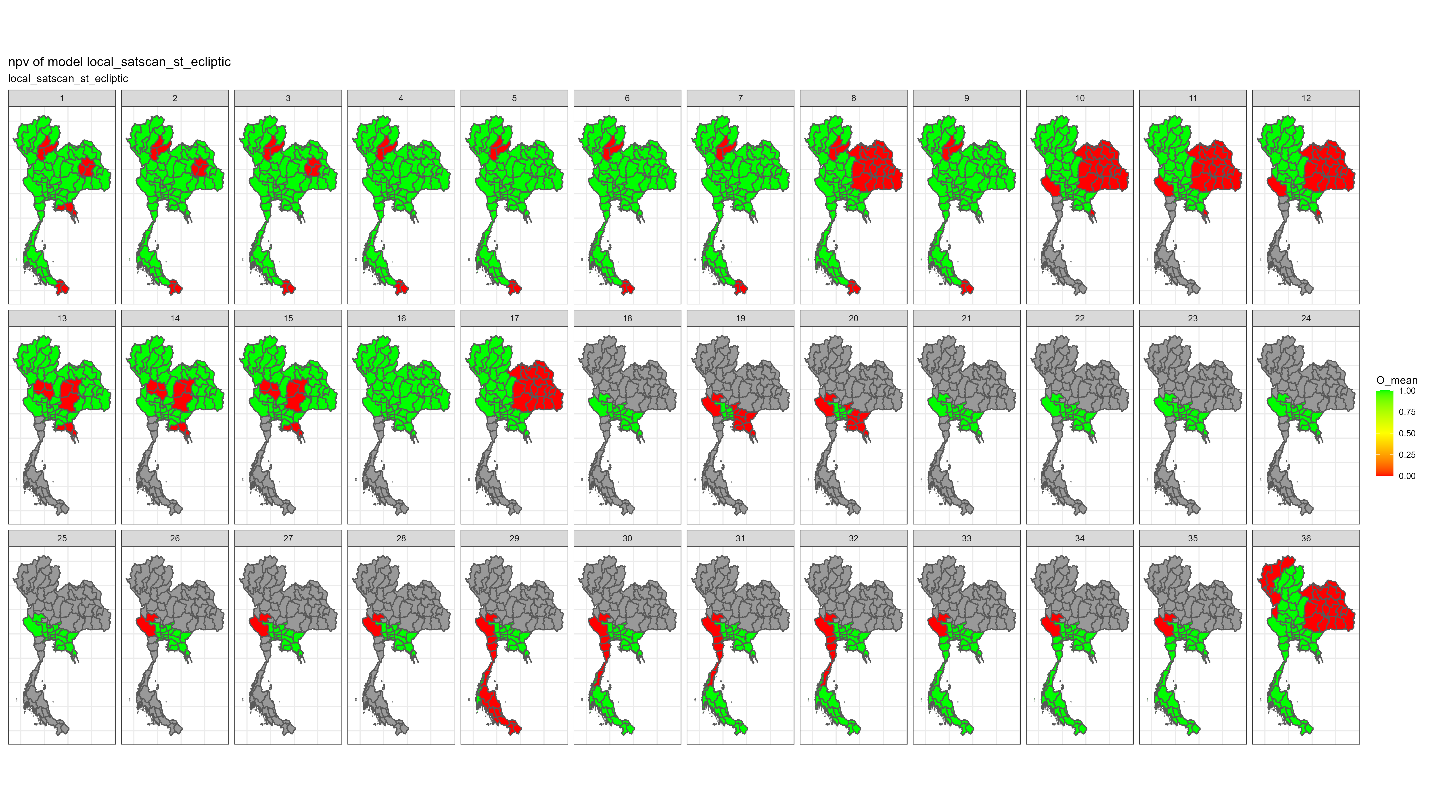


NPV maps of retrospective ST SaTScan, elliptical windowing function, generated using RStudio version 2022.07.0+548 (available at https://posit.co/products/open-source/rstudio/).


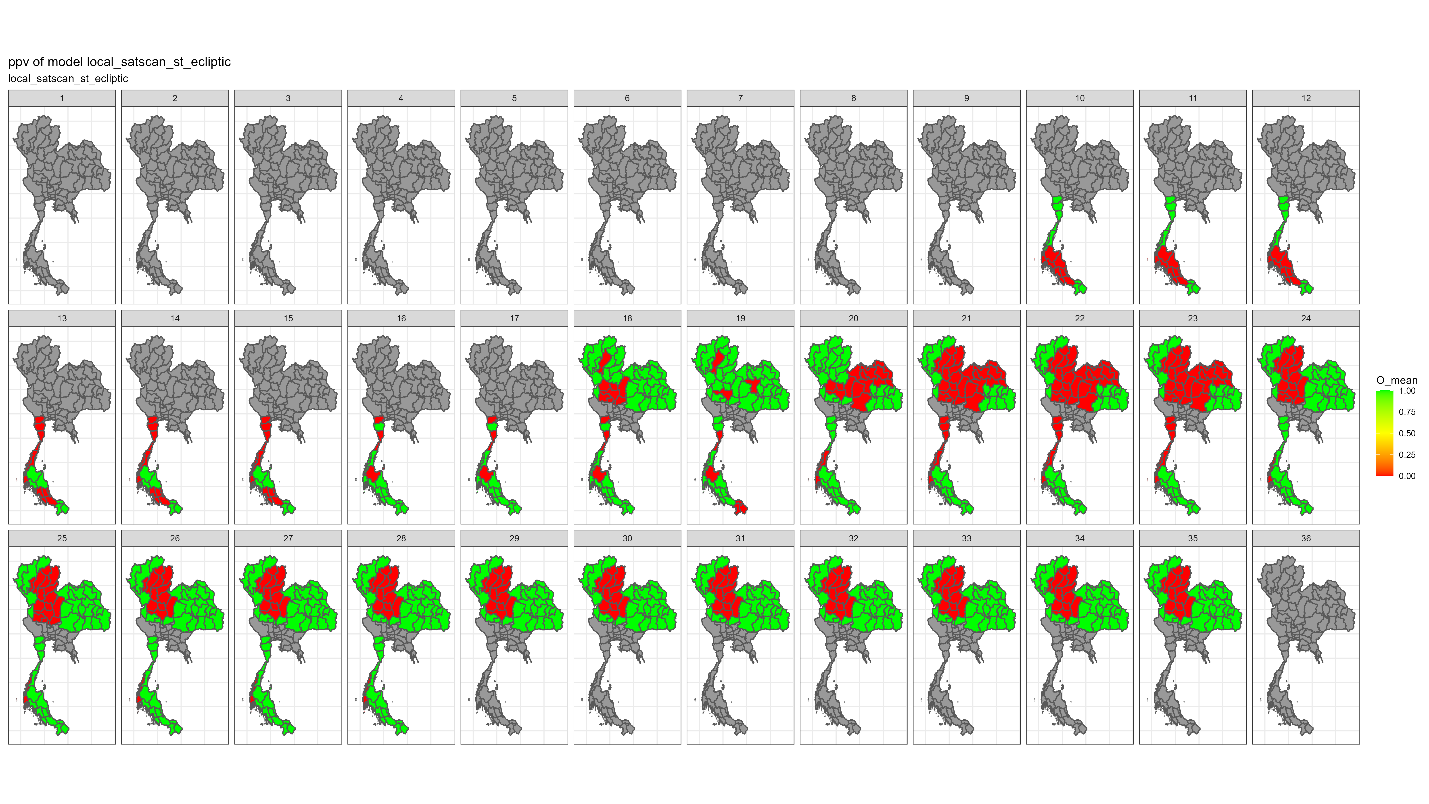


PPV maps of retrospective ST SaTScan, elliptical windowing function, generated using RStudio version 2022.07.0+548 (available at https://posit.co/products/open-source/rstudio/).


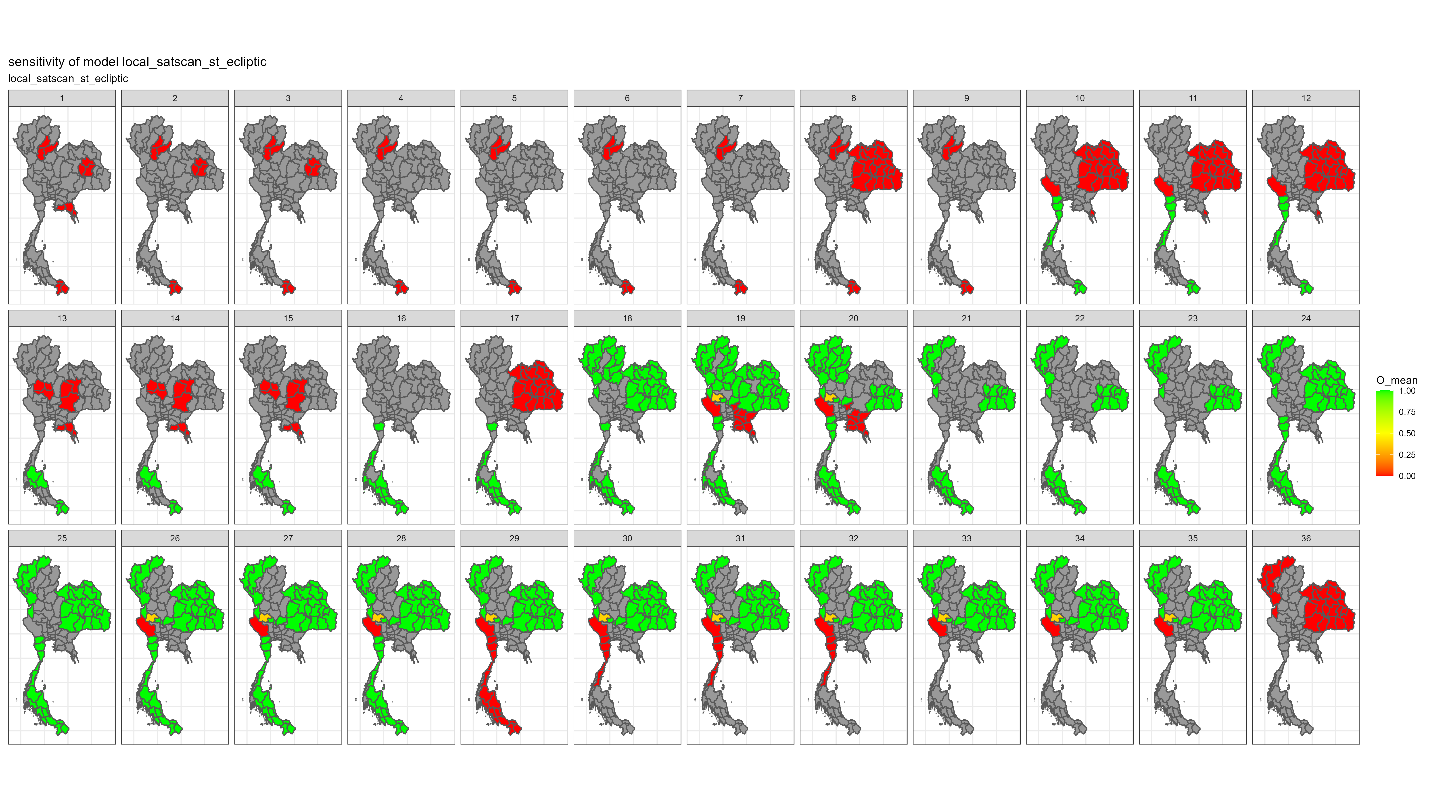


Sensitivity maps of retrospective ST SaTScan, elliptical windowing function, generated using RStudio version 2022.07.0+548 (available at https://posit.co/products/open-source/rstudio/).


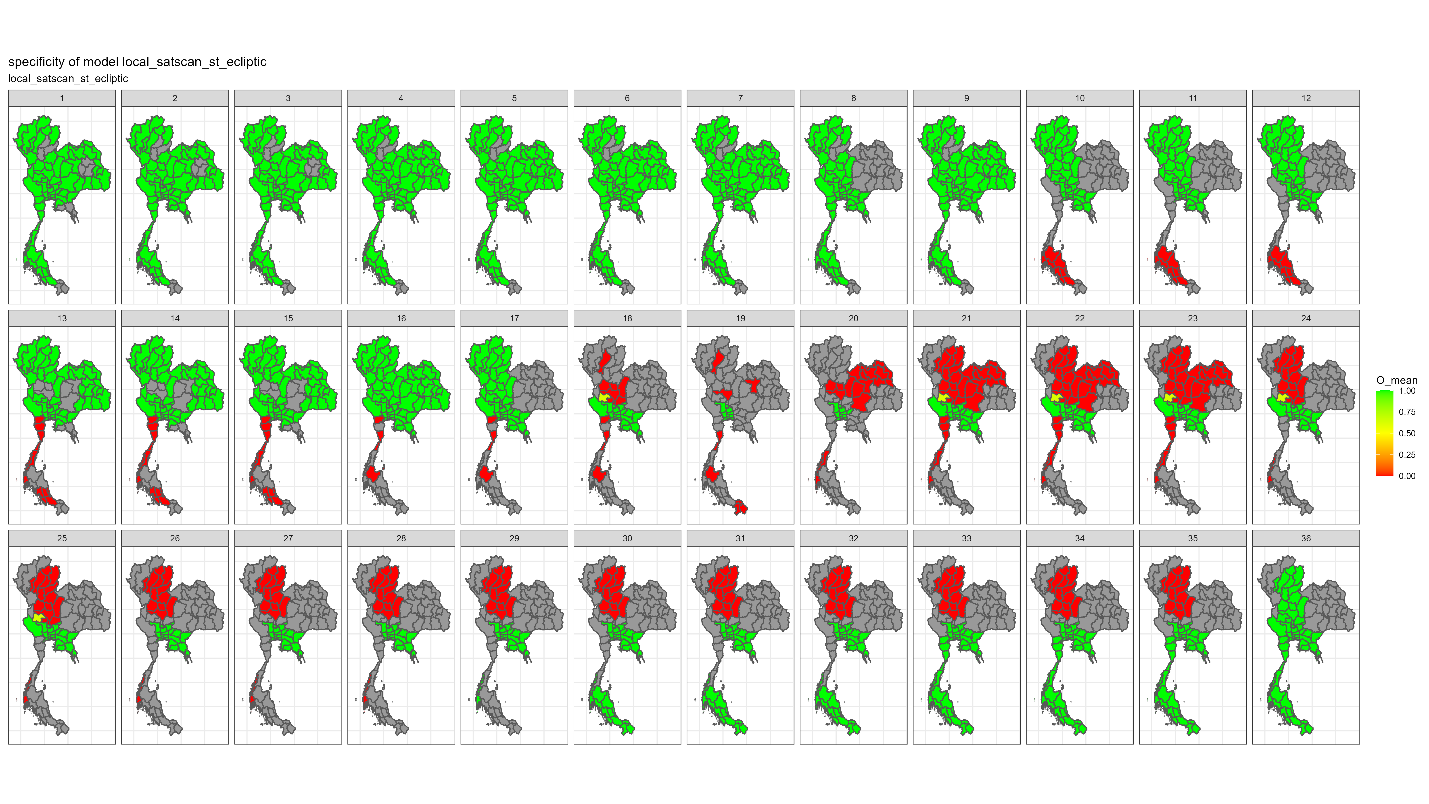


Specificity maps of retrospective ST SaTScan, elliptical windowing function, generated using RStudio version 2022.07.0+548 (available at https://posit.co/products/open-source/rstudio/).

*Space-time Satscan with circular scanning window (retrospective)*


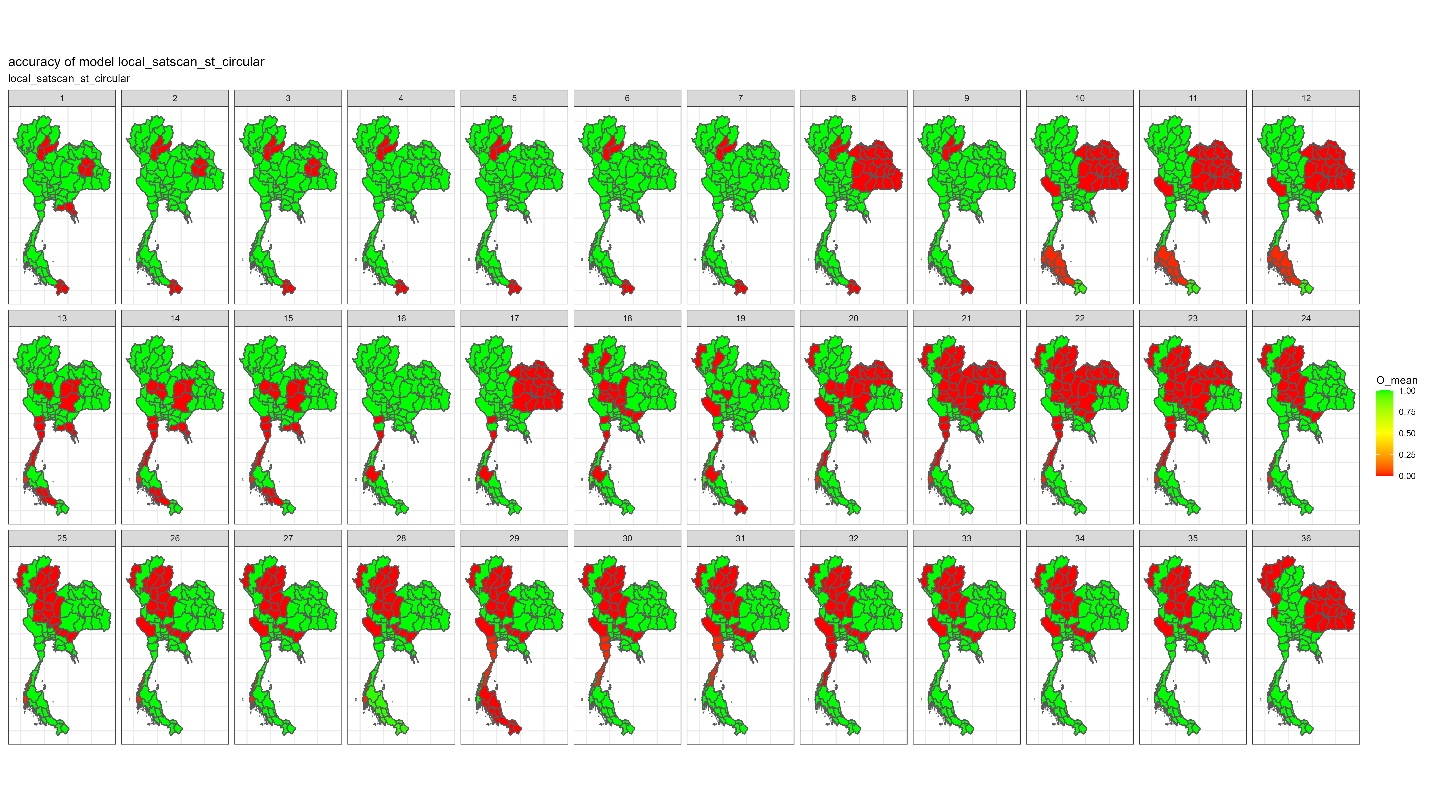


Accuracy maps of retrospective ST SaTScan, circular windowing function, generated using RStudio version 2022.07.0+548 (available at https://posit.co/products/open-source/rstudio/).


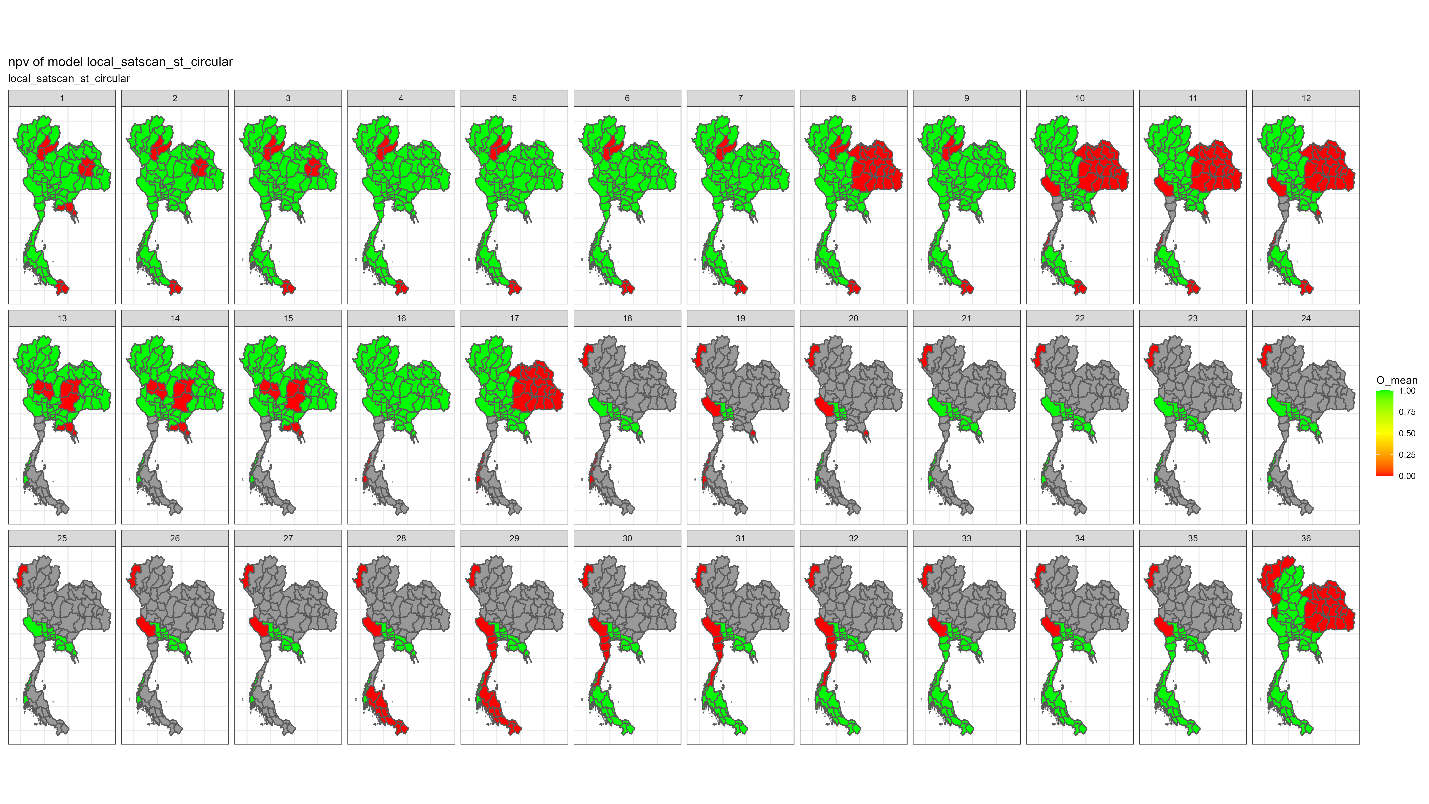


NPV maps of retrospective ST SaTScan, circular windowing function, generated using RStudio version 2022.07.0+548 (available at https://posit.co/products/open-source/rstudio/).


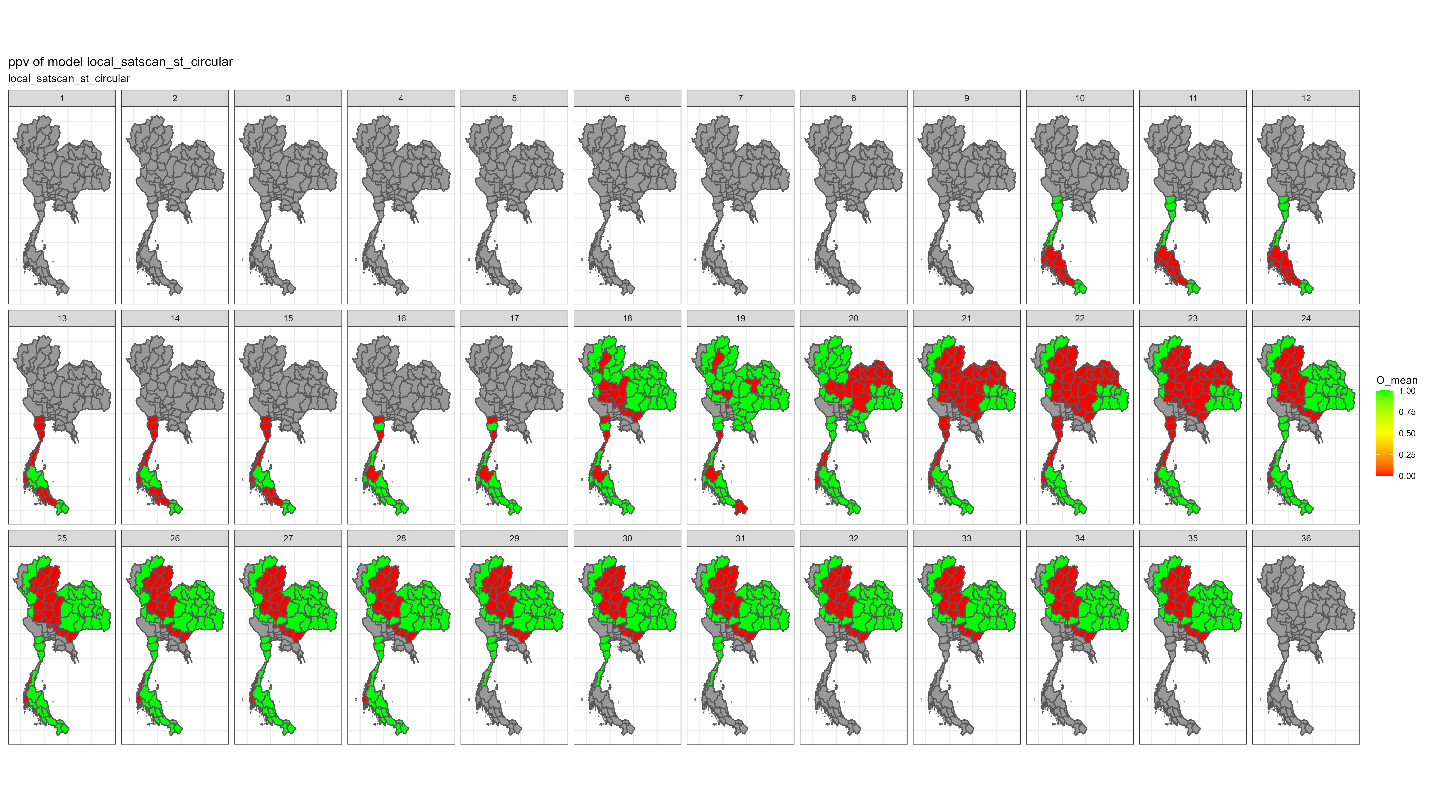


PPV maps of retrospective ST SaTScan, circular windowing function, generated using RStudio version 2022.07.0+548 (available at https://posit.co/products/open-source/rstudio/).


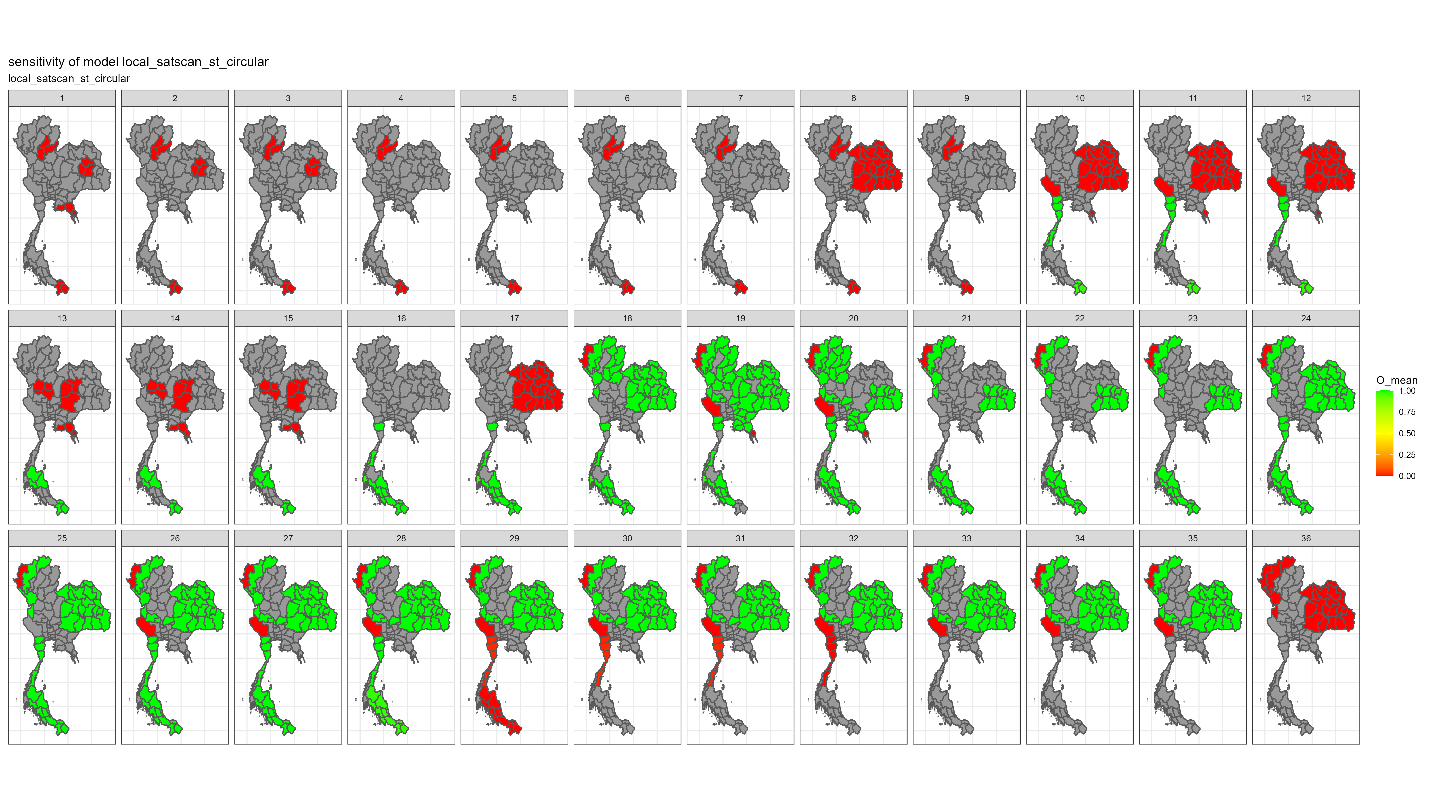


Sensitivity maps of retrospective ST SaTScan, circular windowing function, generated using RStudio version 2022.07.0+548 (available at https://posit.co/products/open-source/rstudio/).


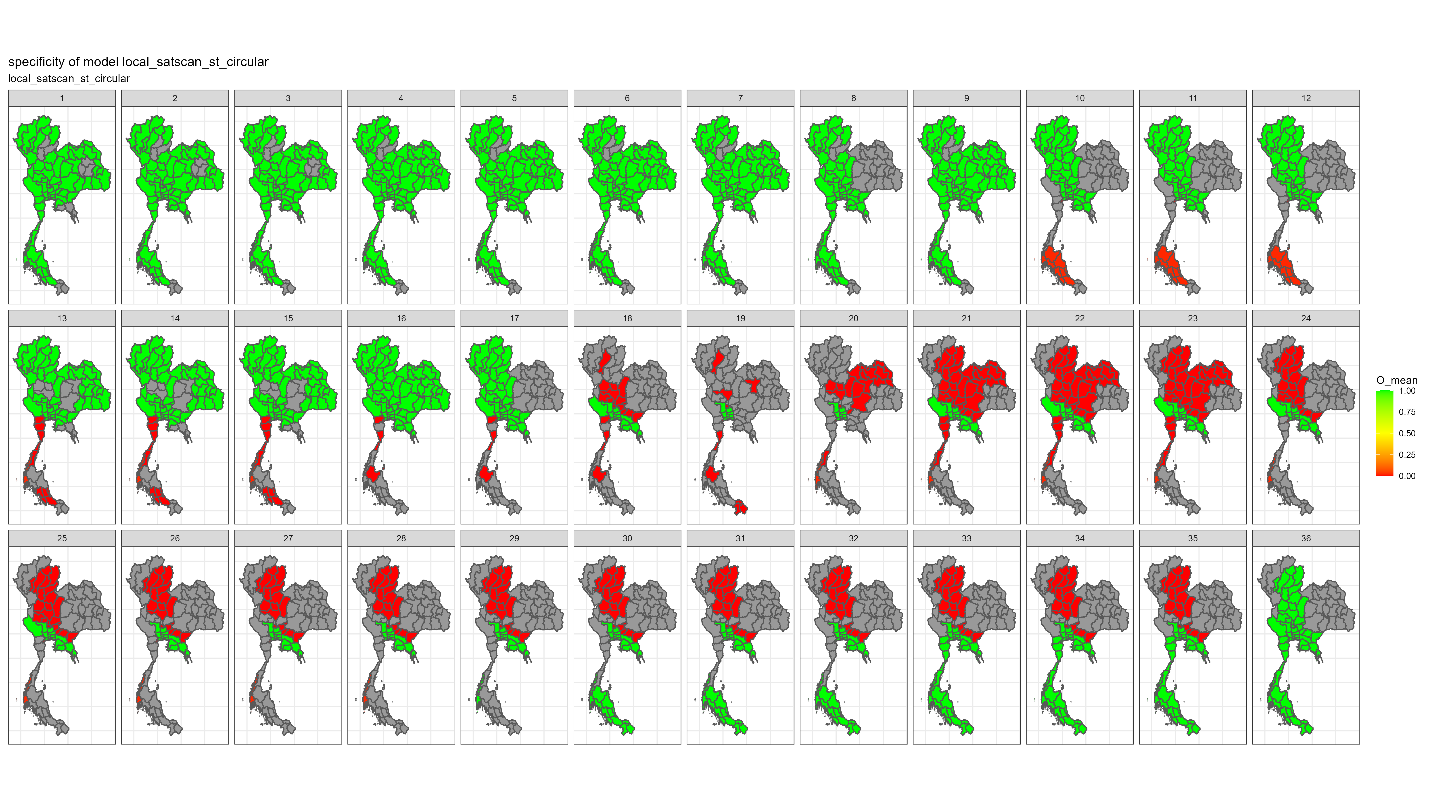


Specificity maps of retrospective ST SaTScan, circular windowing function, generated using RStudio version 2022.07.0+548 (available at https://posit.co/products/open-source/rstudio/).

*Purely spatial SatScan with elliptic scanning window*


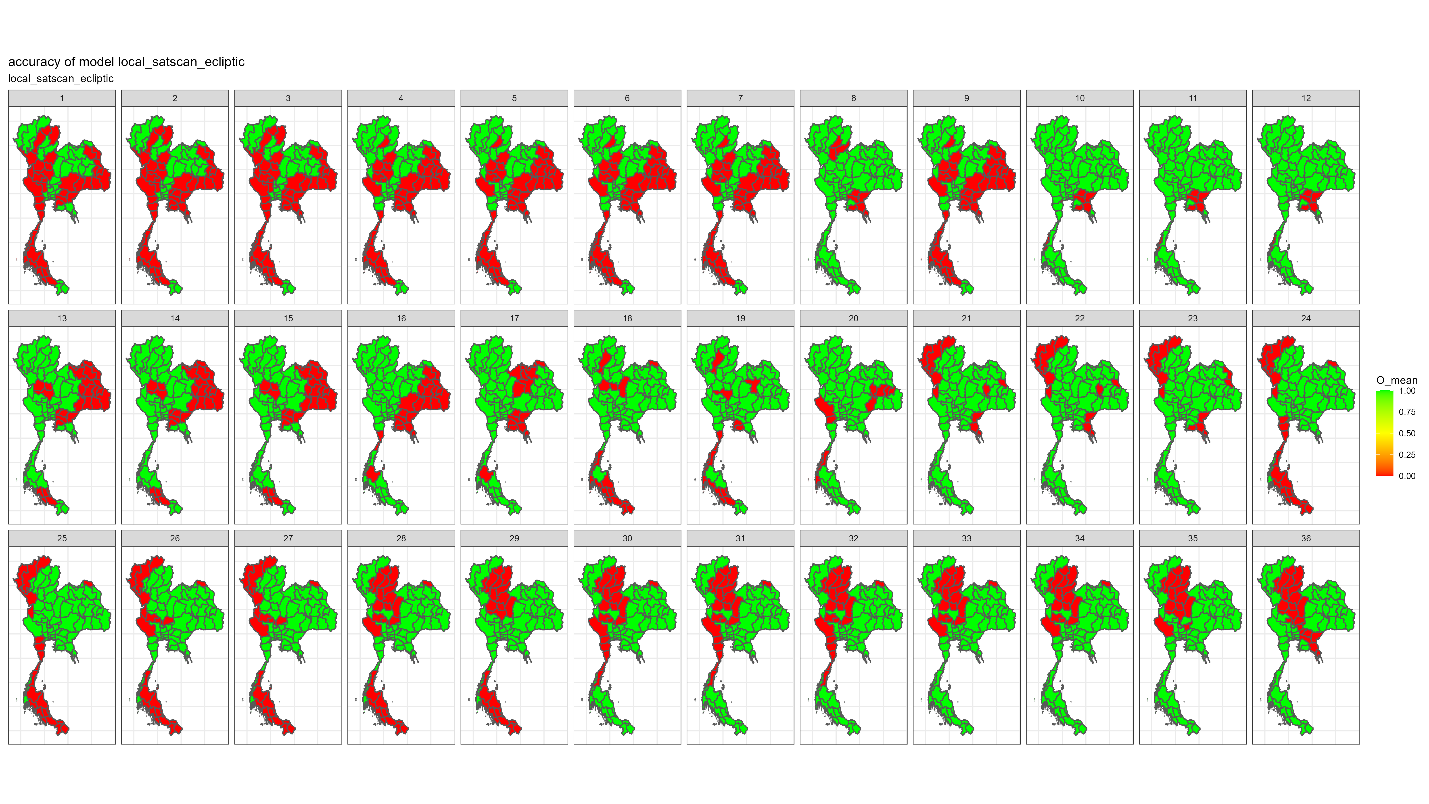


Accuracy maps of spatial SaTScan, elliptical windowing function, generated using RStudio version 2022.07.0+548 (available at https://posit.co/products/open-source/rstudio/).


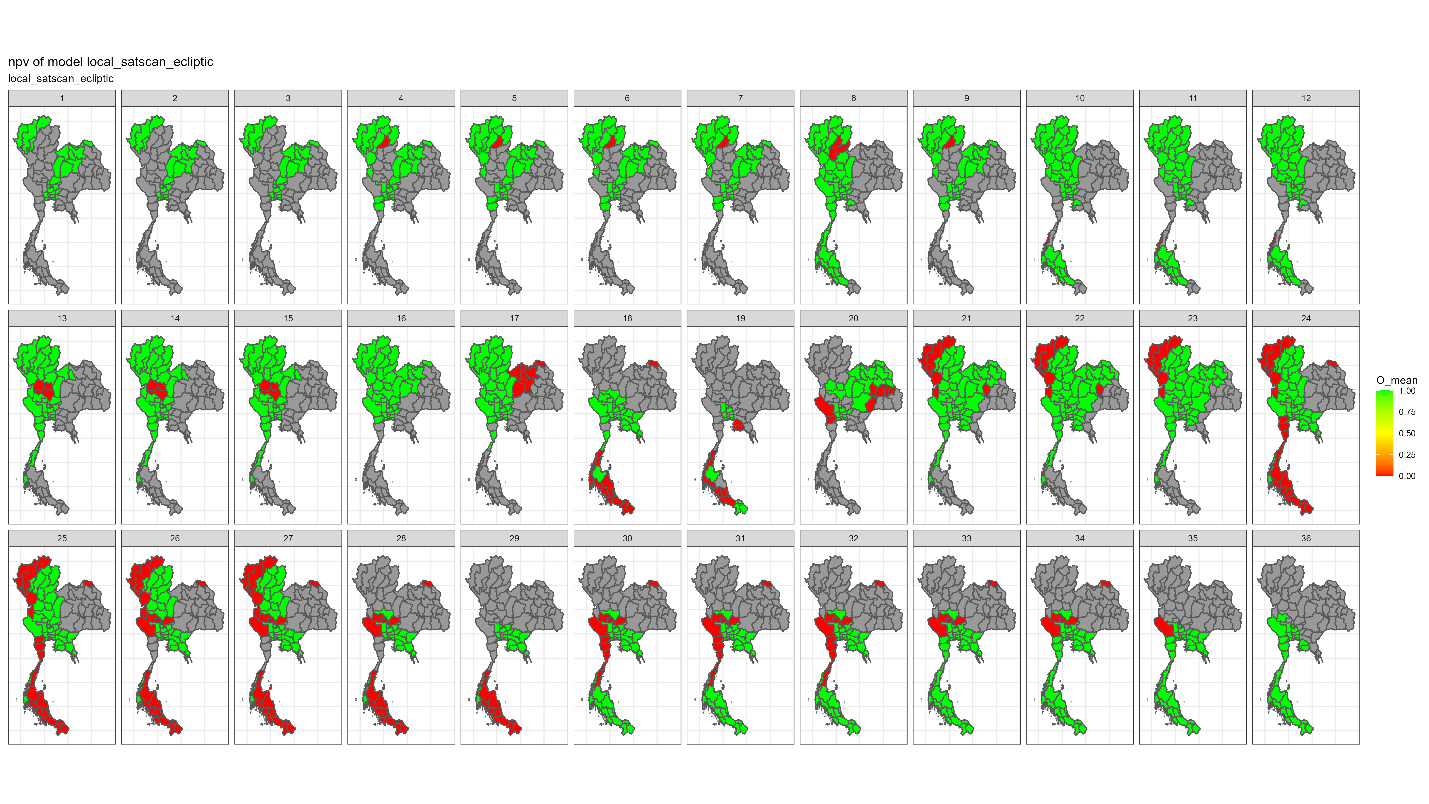


NPV maps of spatial SaTScan, elliptical windowing function, generated using RStudio version 2022.07.0+548 (available at https://posit.co/products/open-source/rstudio/).


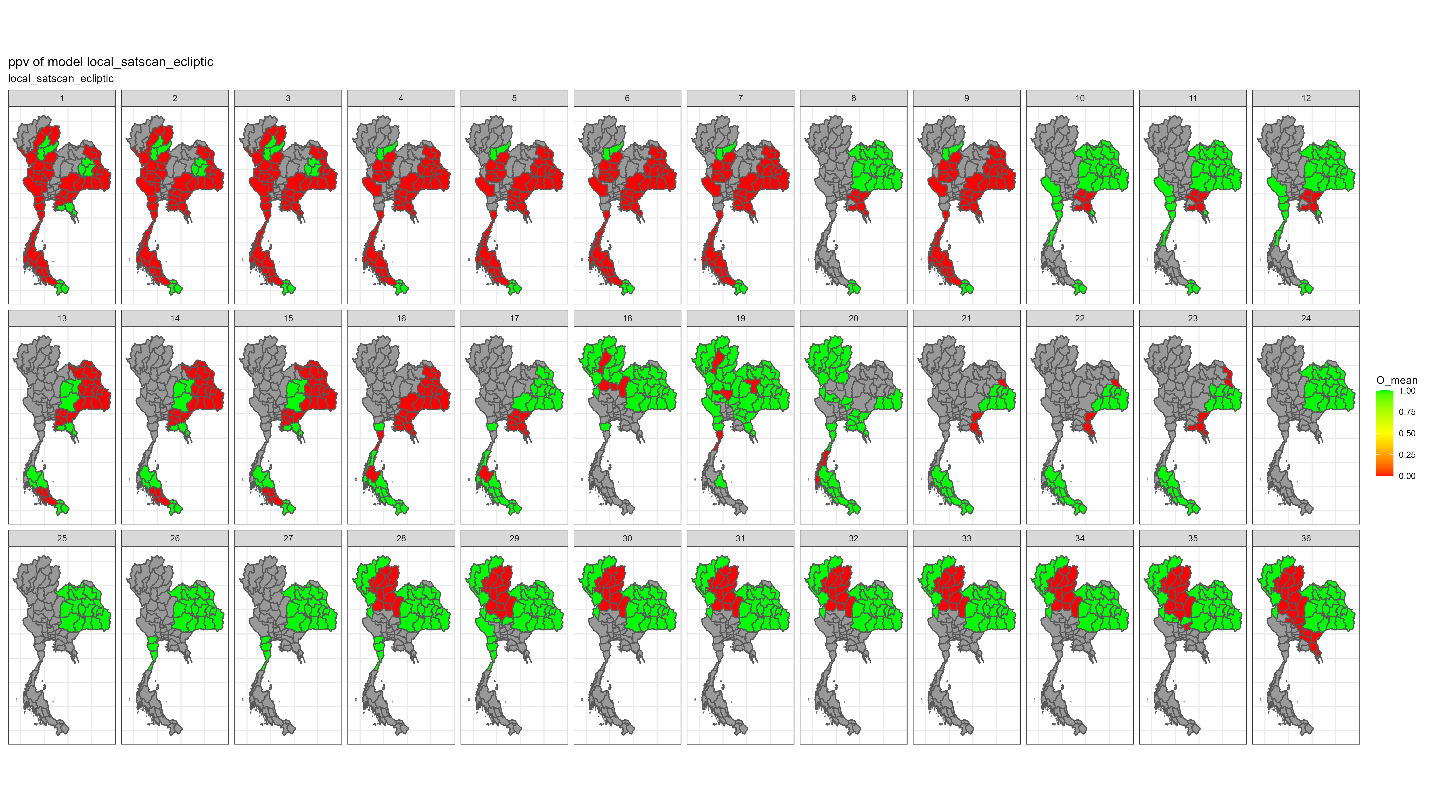


PPV maps of spatial SaTScan, elliptical windowing function, generated using RStudio version 2022.07.0+548 (available at https://posit.co/products/open-source/rstudio/).


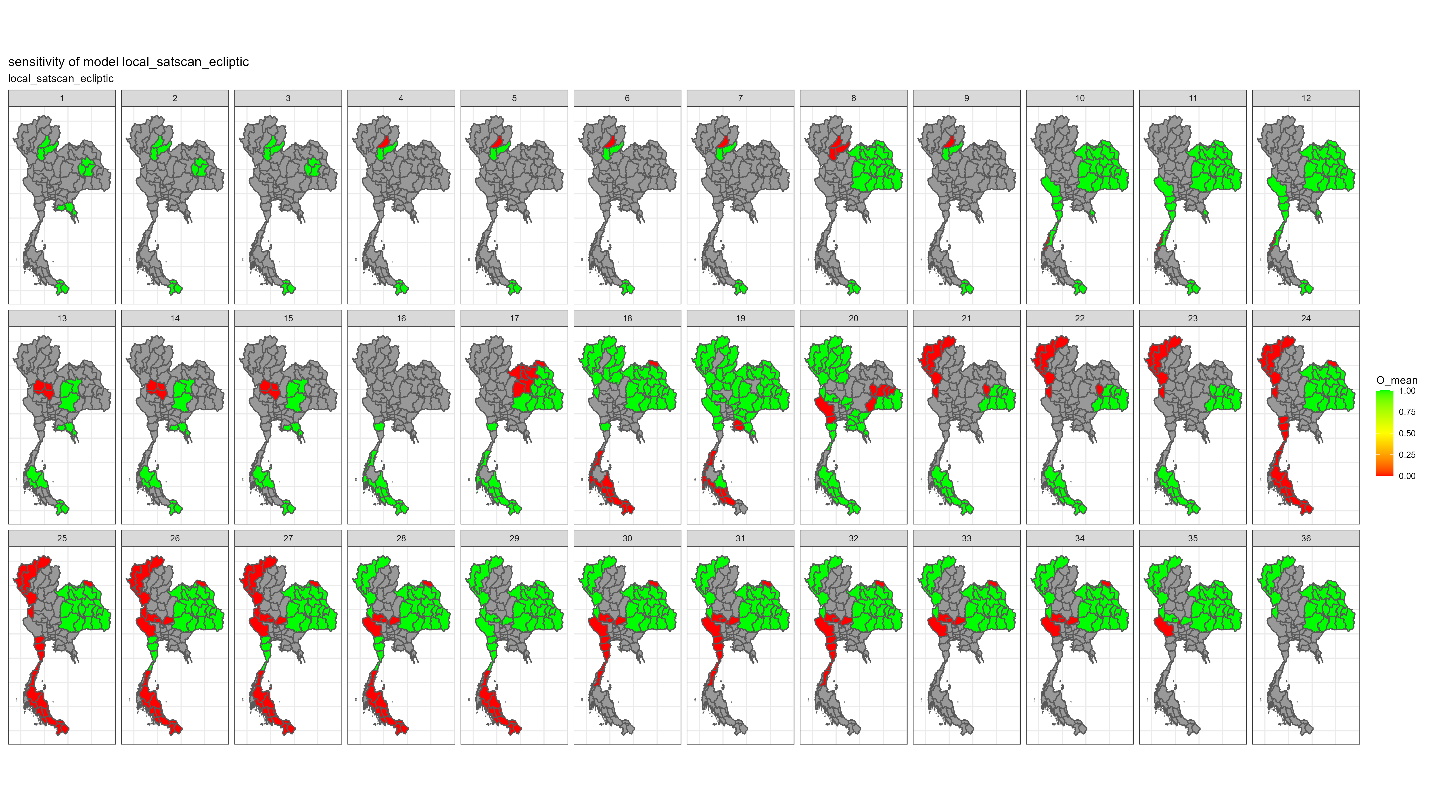


Sensitivity maps of spatial SaTScan, elliptical windowing function, generated using RStudio version 2022.07.0+548 (available at https://posit.co/products/open-source/rstudio/).


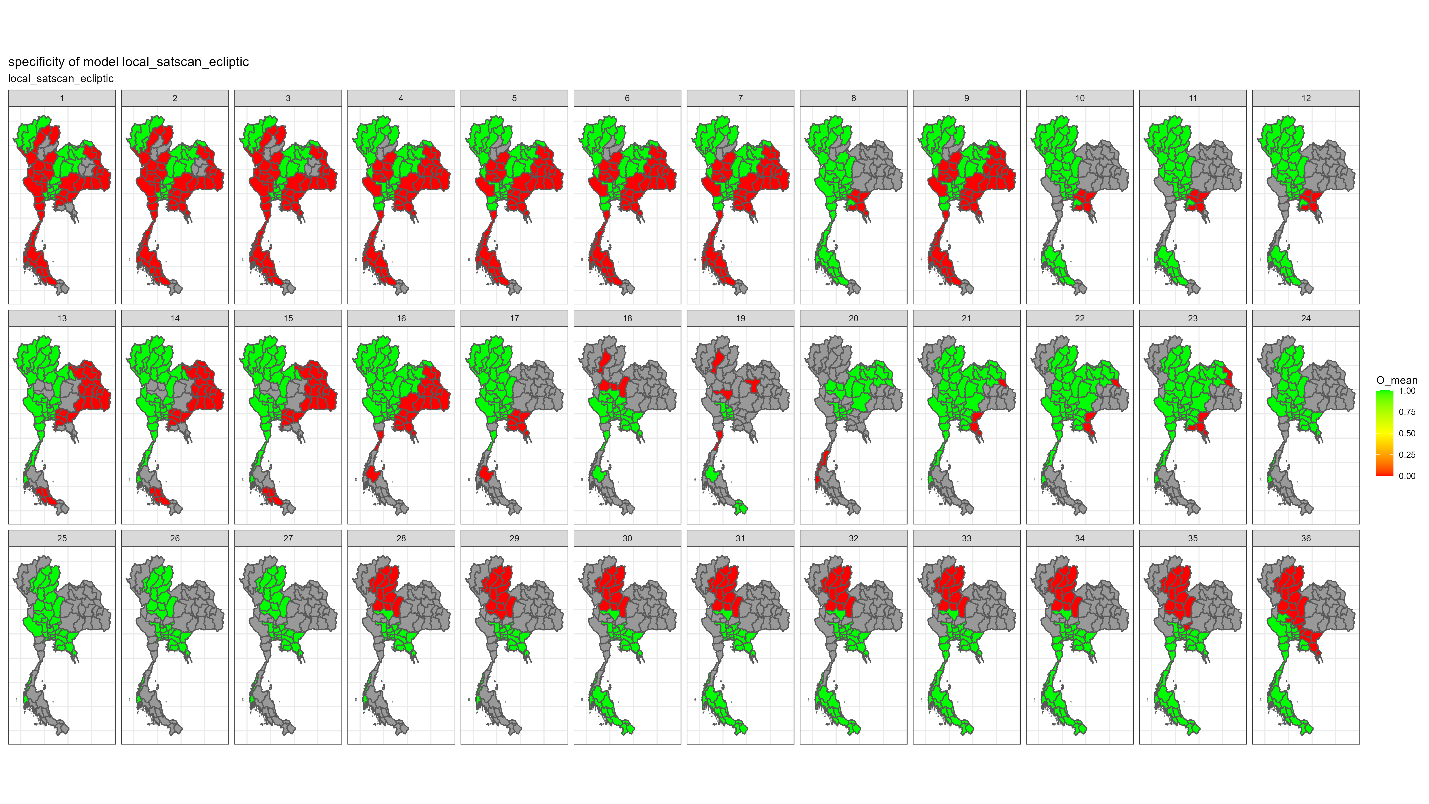


Specificity maps of spatial SaTScan, elliptical windowing function, generated using RStudio version 2022.07.0+548 (available at https://posit.co/products/open-source/rstudio/).

*Purely spatial SatScan with cicular scanning window*


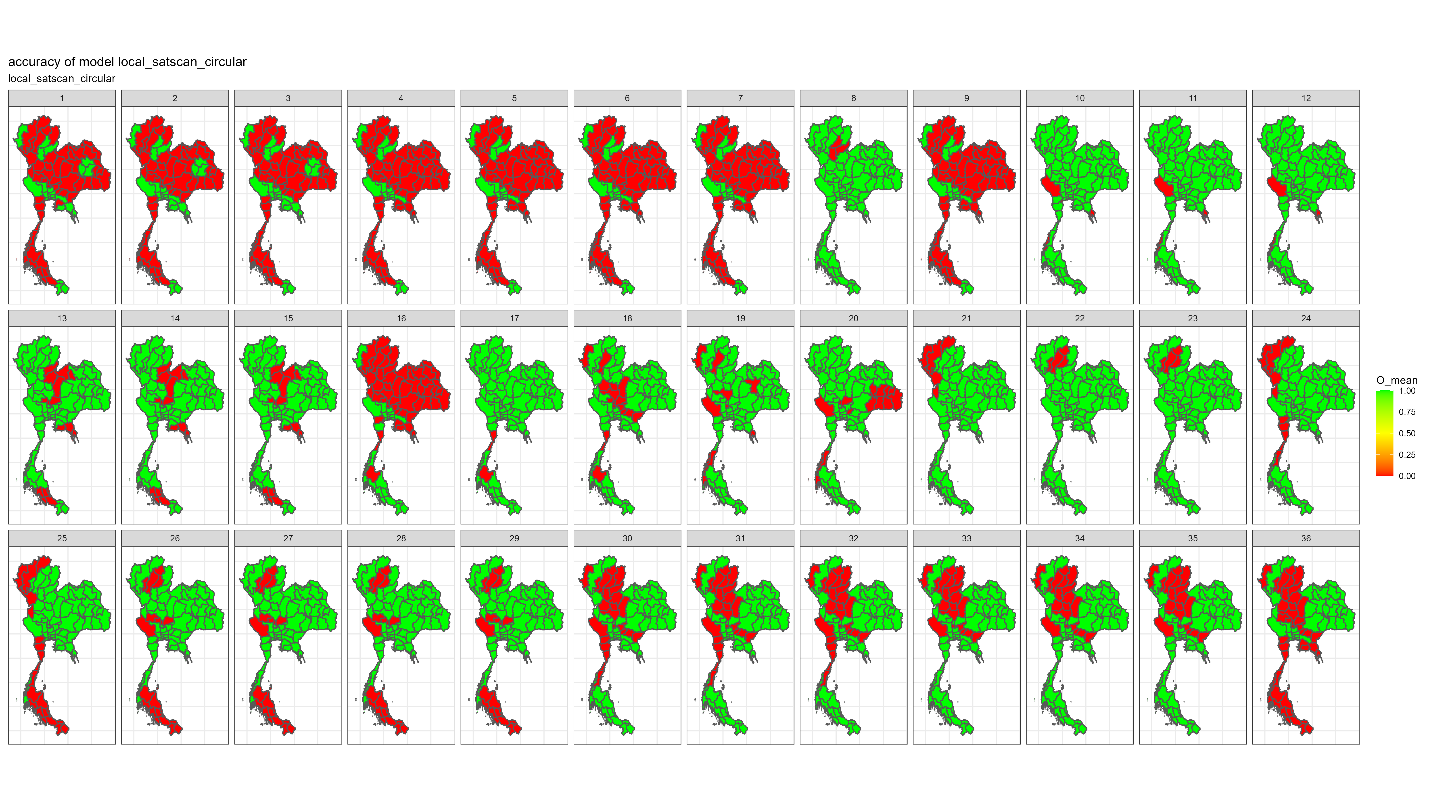


Accuracy maps of spatial SaTScan, circular windowing function, generated using RStudio version 2022.07.0+548 (available at https://posit.co/products/open-source/rstudio/).


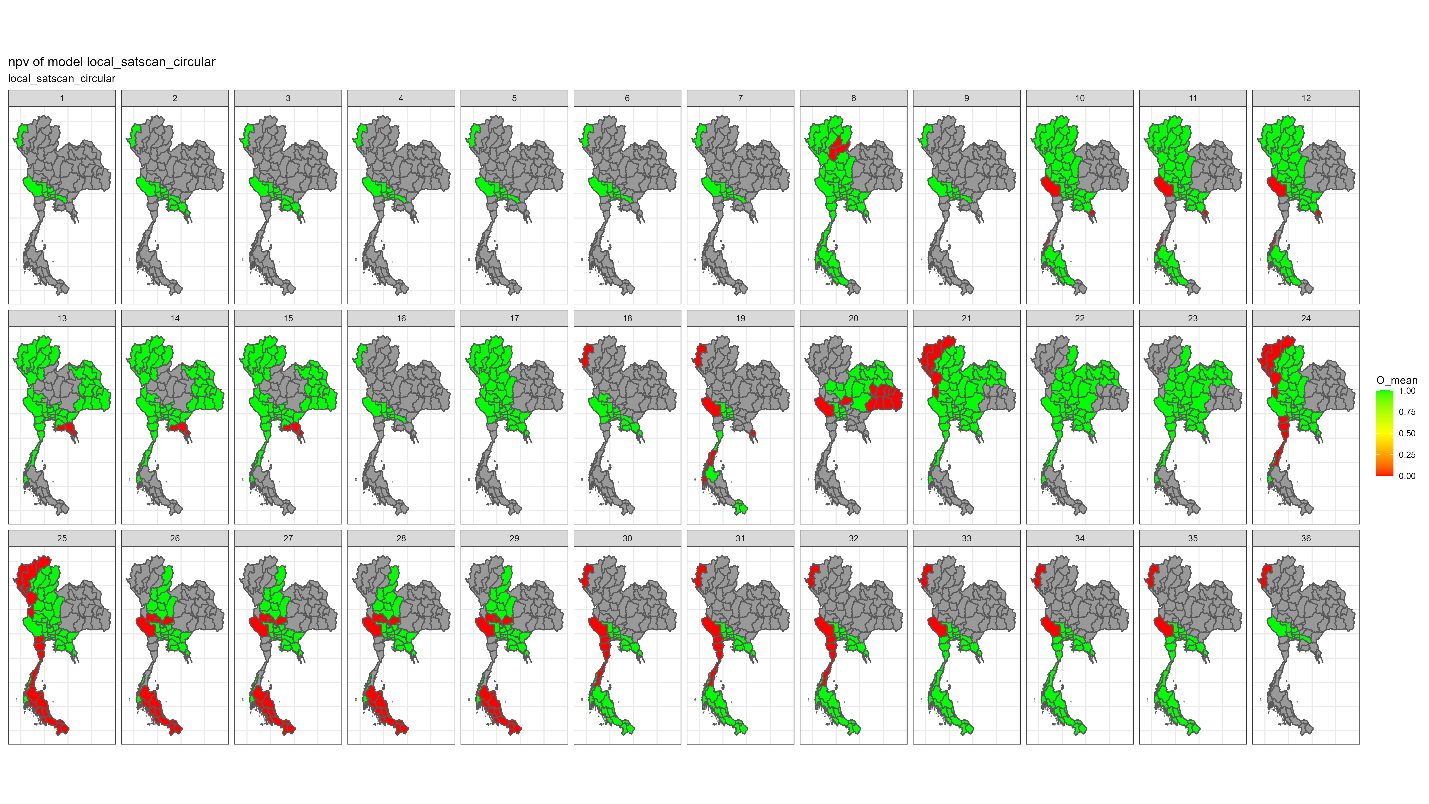


NPV maps of spatial SaTScan, circular windowing function, generated using RStudio version 2022.07.0+548 (available at https://posit.co/products/open-source/rstudio/).


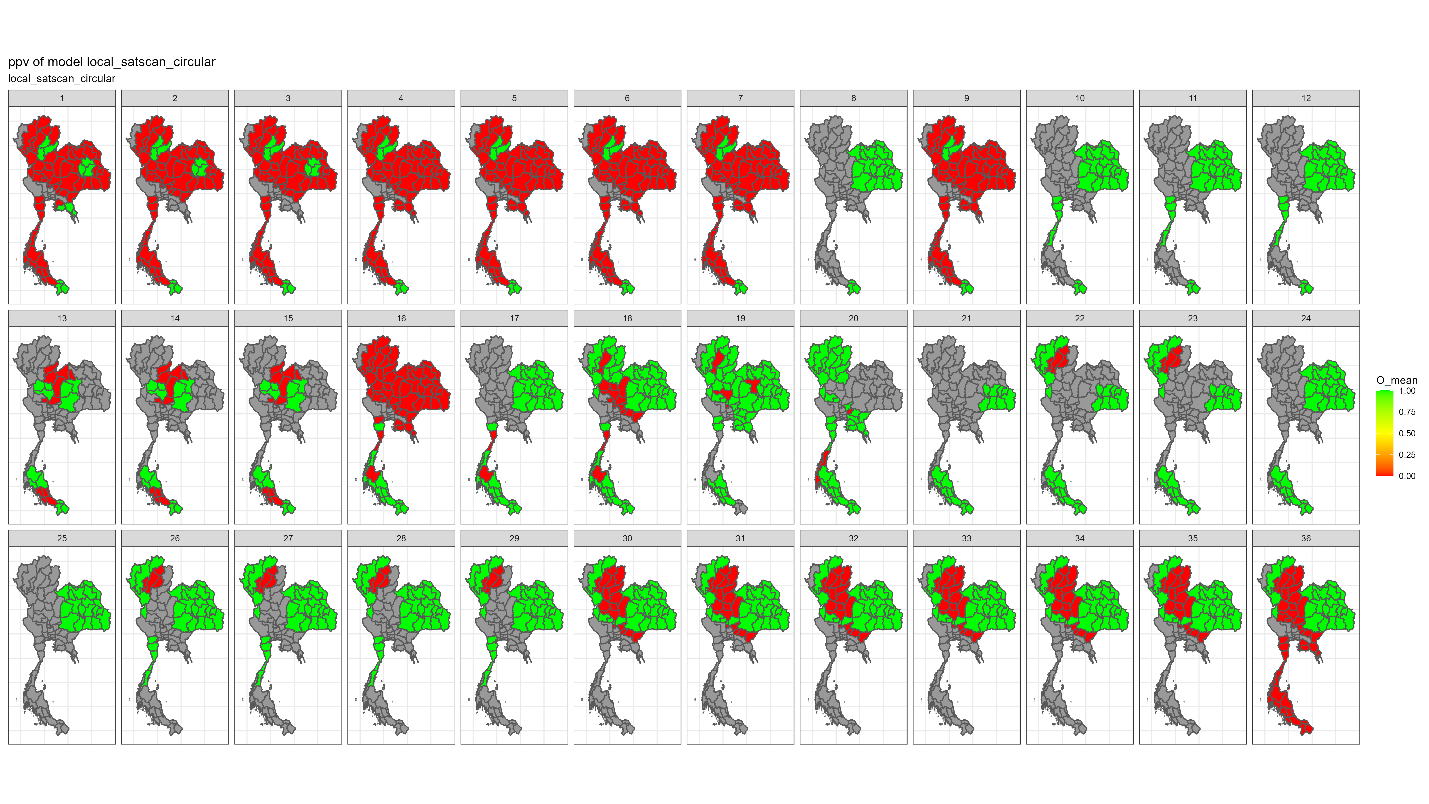


PPV maps of spatial SaTScan, circular windowing function, generated using RStudio version 2022.07.0+548 (available at https://posit.co/products/open-source/rstudio/).


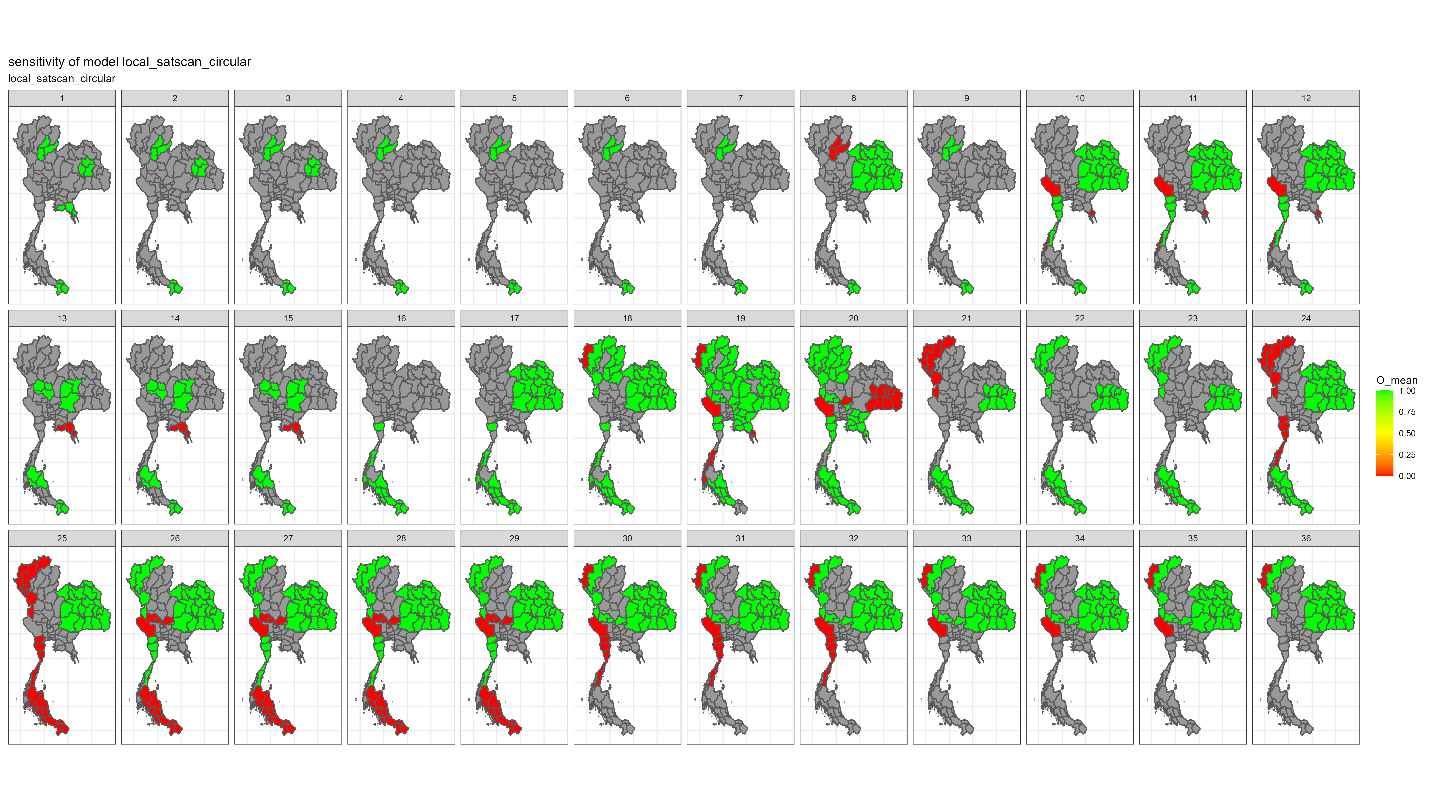


Sensitivity maps of spatial SaTScan, circular windowing function, generated using RStudio version 2022.07.0+548 (available at https://posit.co/products/open-source/rstudio/).


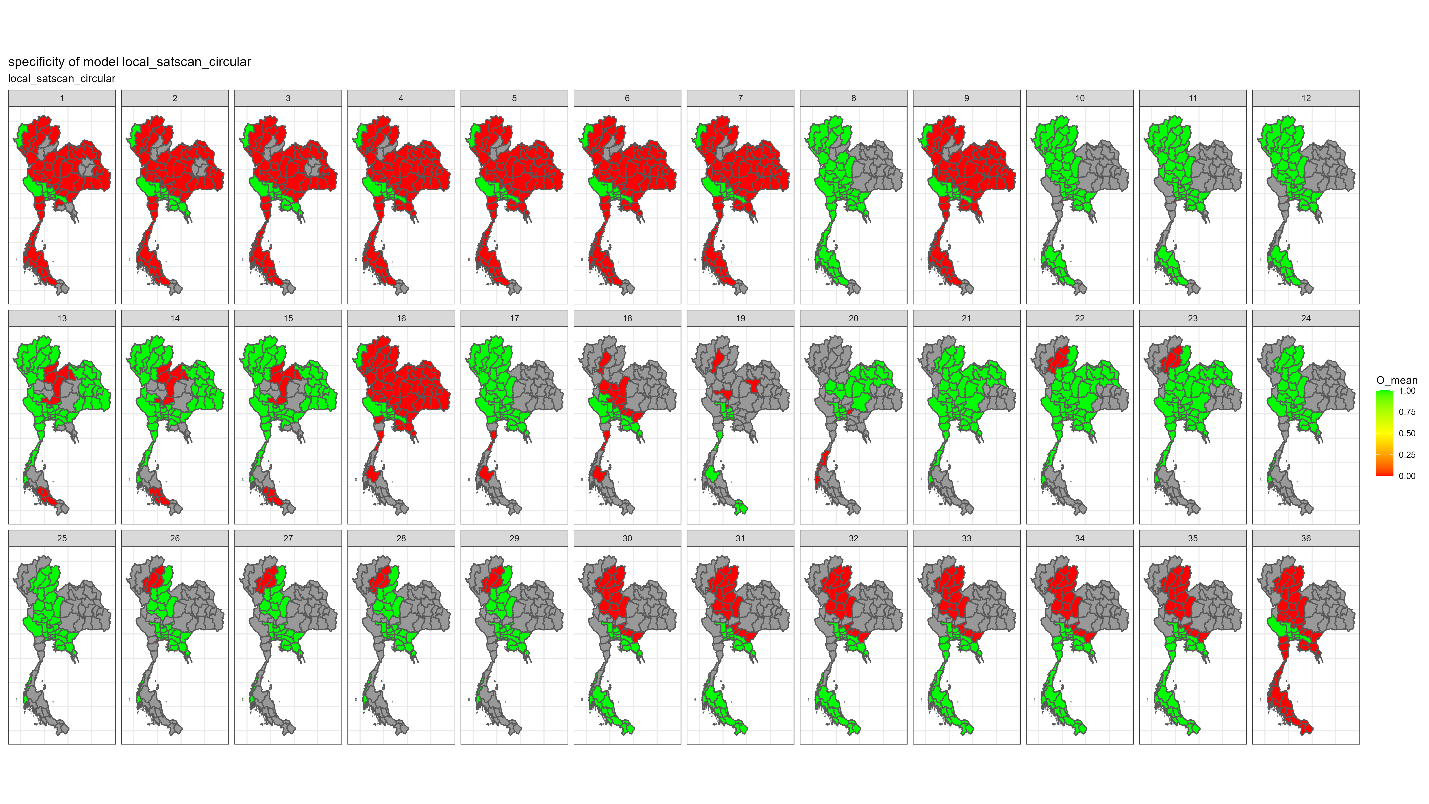


Specificity maps of spatial SaTScan, circular windowing function, generated using RStudio version 2022.07.0+548 (available at https://posit.co/products/open-source/rstudio/).

*Space-time Satscan with circular scanning window (prospective)*


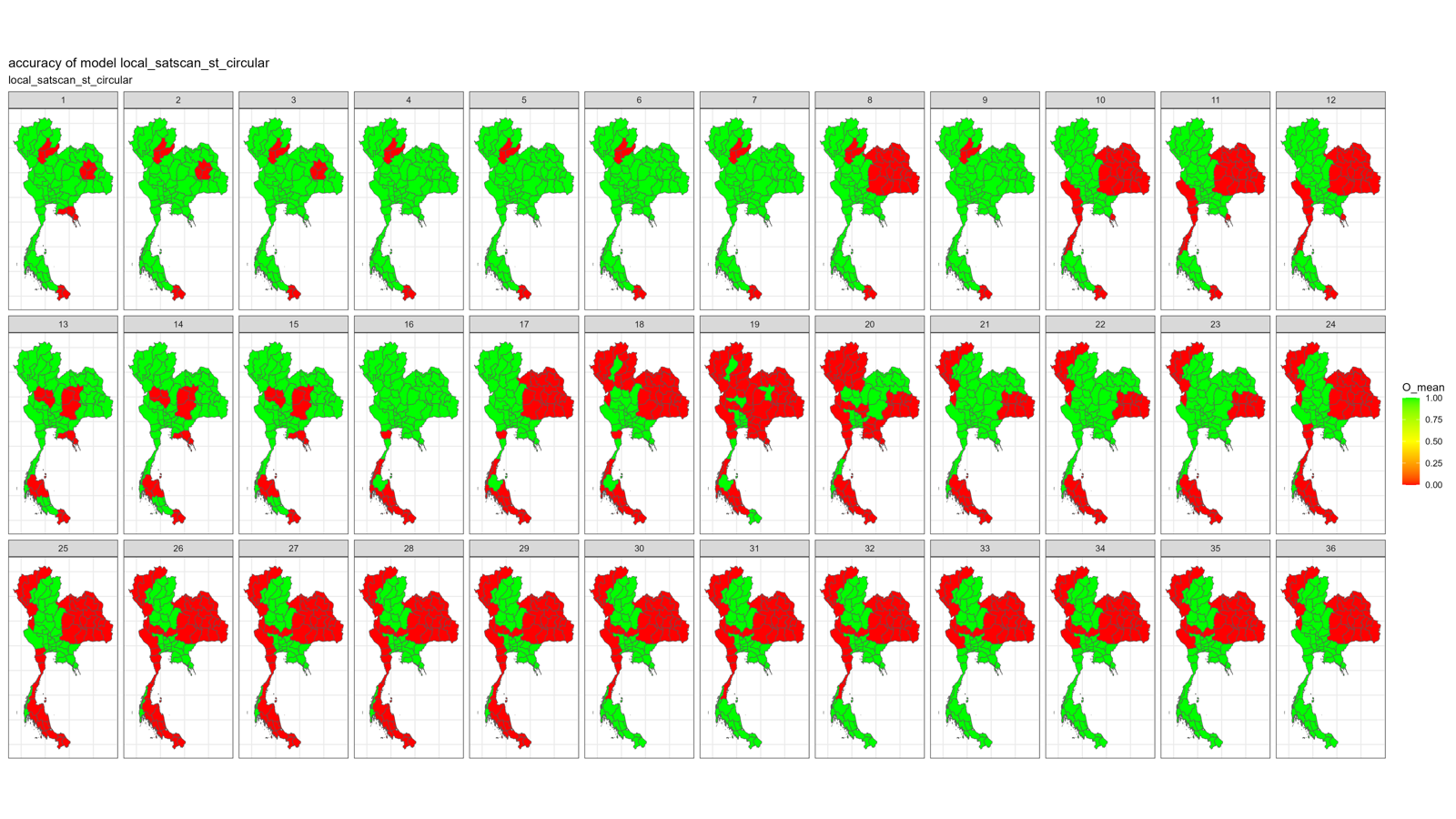


Accuracy maps of retrospective ST SaTScan, circular windowing function, generated using RStudio version 2022.07.0+548 (available at https://posit.co/products/open-source/rstudio/).


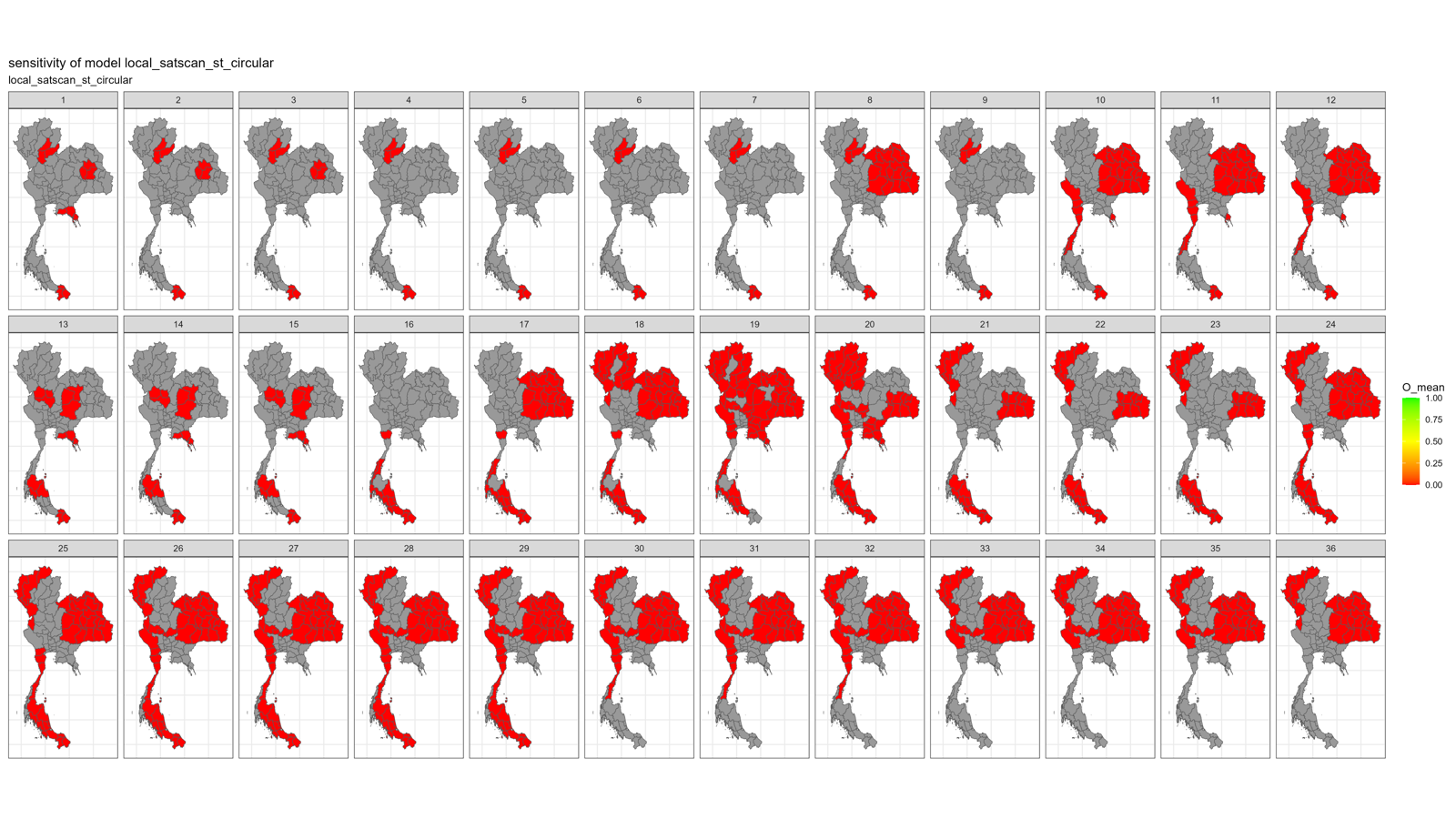


Sensitivity maps of retrospective ST SaTScan, circular windowing function, generated using RStudio version 2022.07.0+548 (available at https://posit.co/products/open-source/rstudio/).


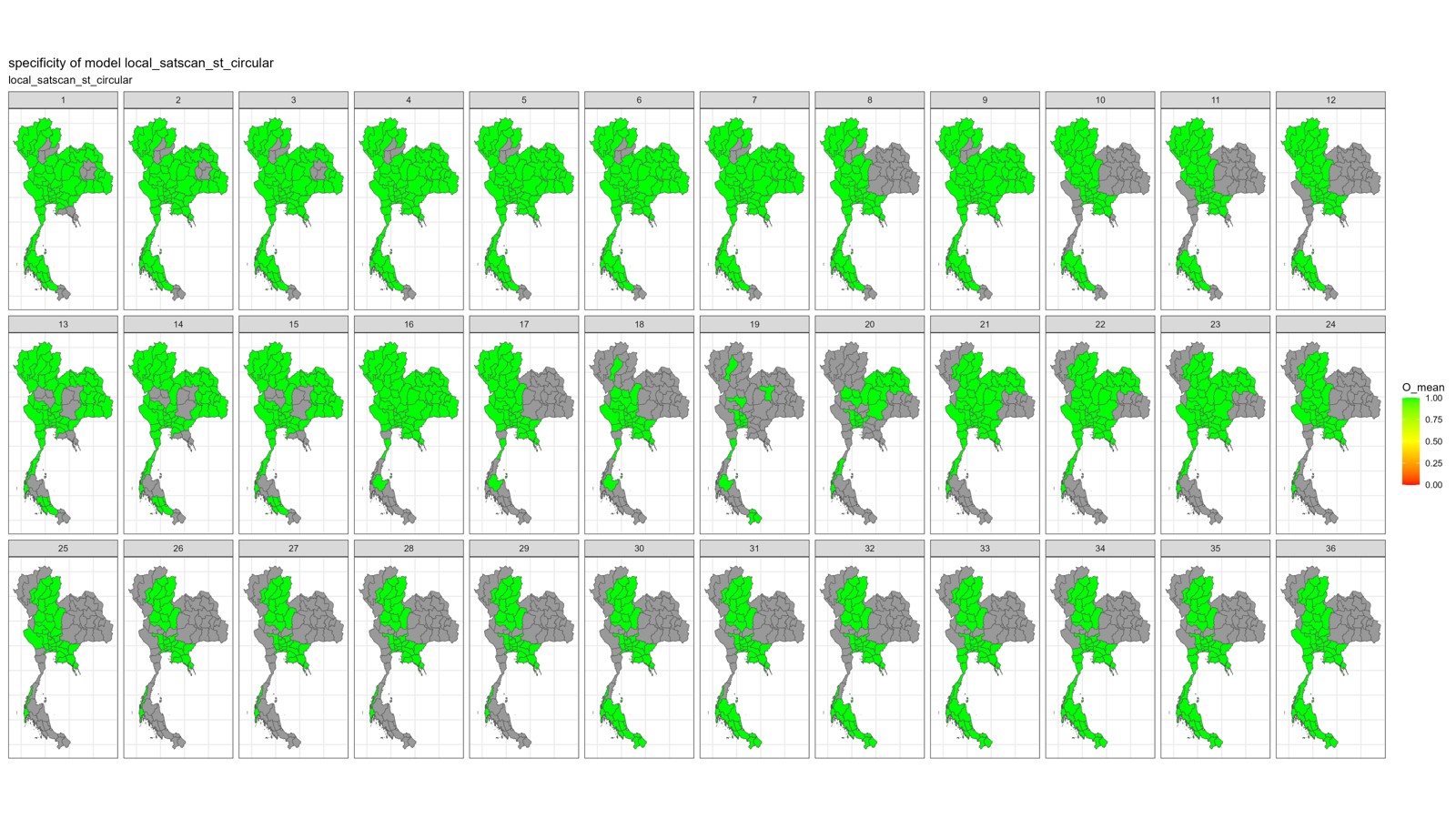


Specificity maps of retrospective ST SaTScan, circular windowing function, generated using RStudio version 2022.07.0+548 (available at https://posit.co/products/open-source/rstudio/).


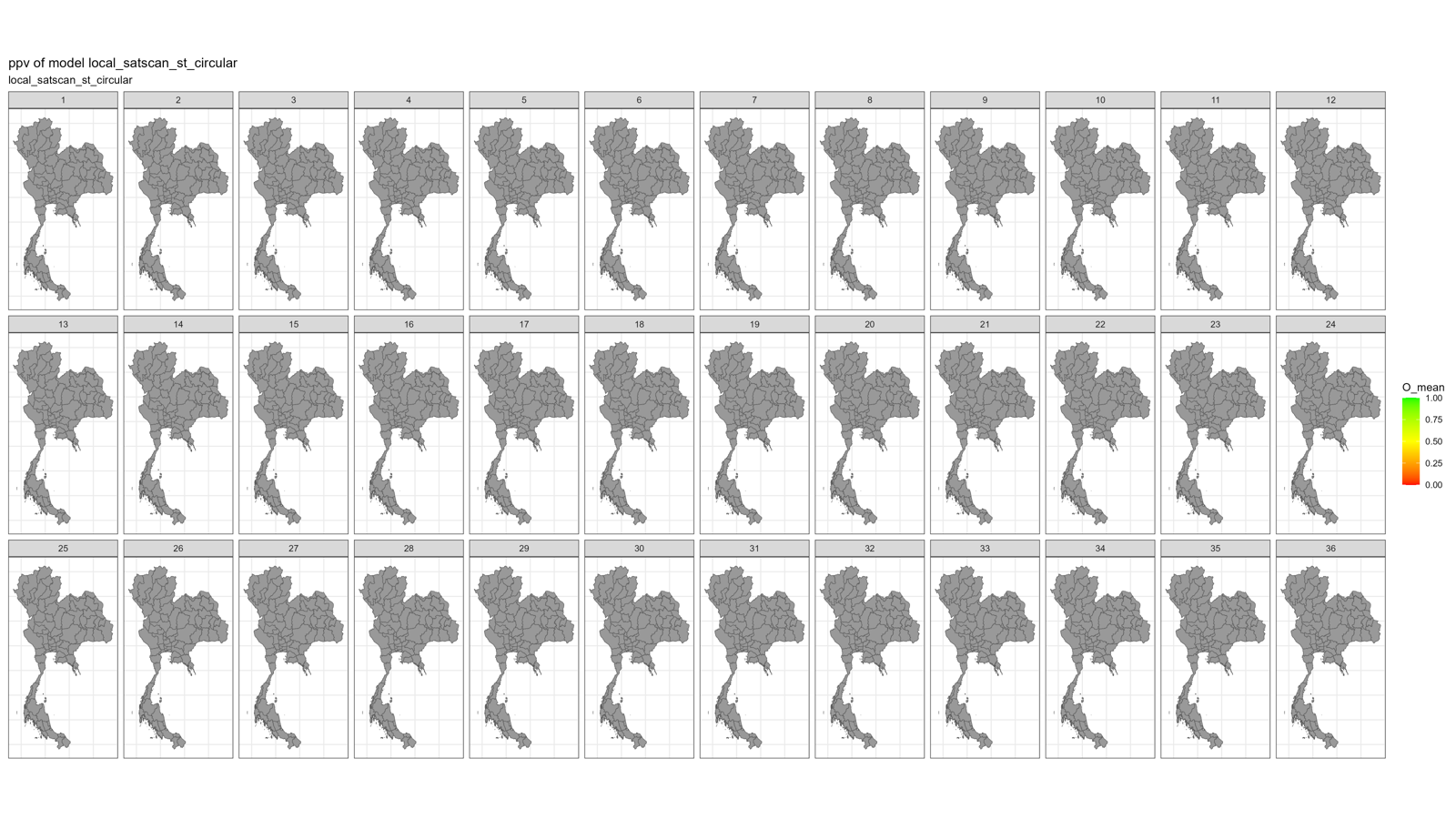


PPV maps of retrospective ST SaTScan, circular windowing function, generated using RStudio version 2022.07.0+548 (available at https://posit.co/products/open-source/rstudio/).


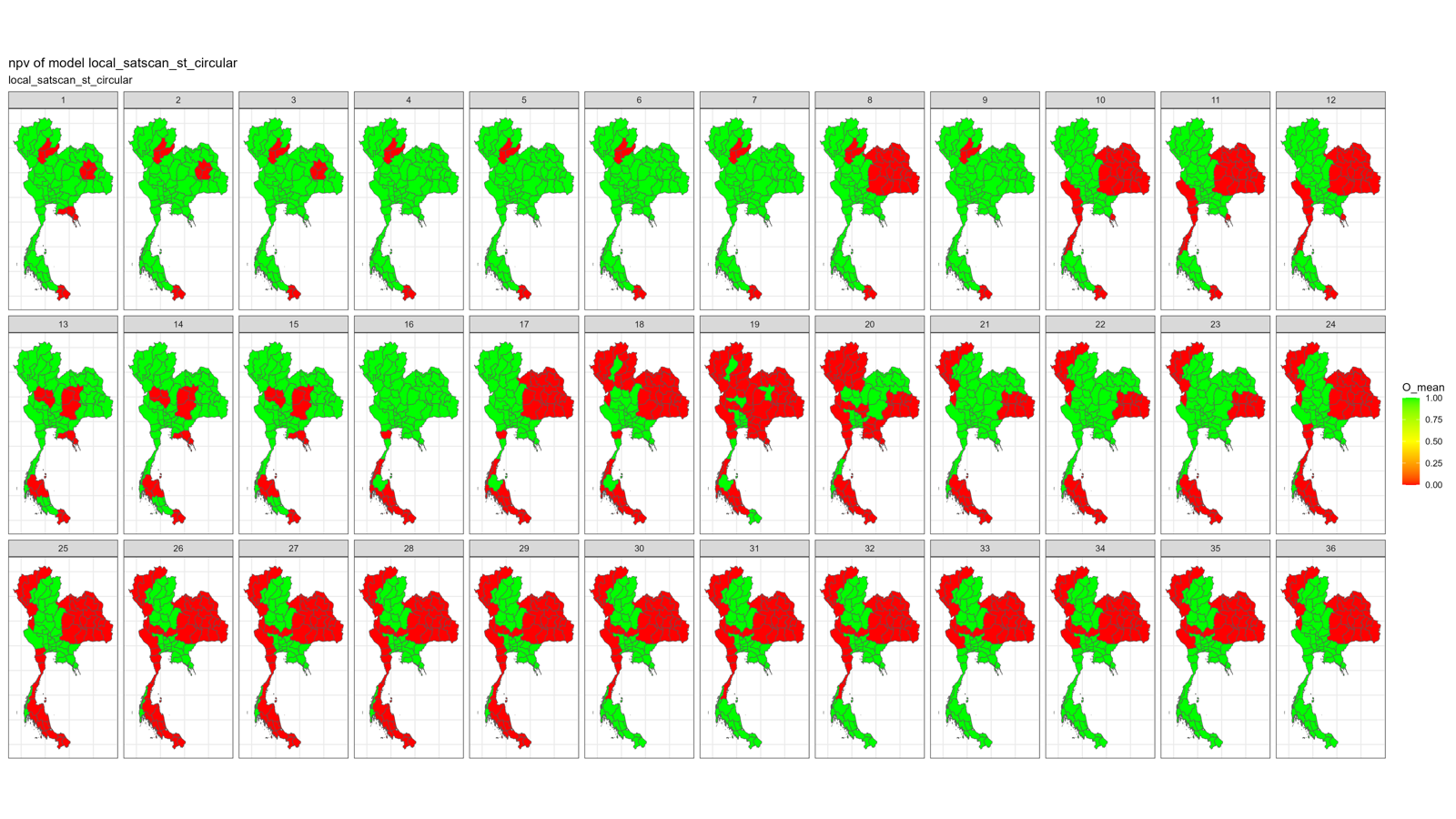


NPV maps of retrospective ST SaTScan, circular windowing function, generated using RStudio version 2022.07.0+548 (available at https://posit.co/products/open-source/rstudio/).

*Space-time Satscan with elliptic scanning window (prospective)*


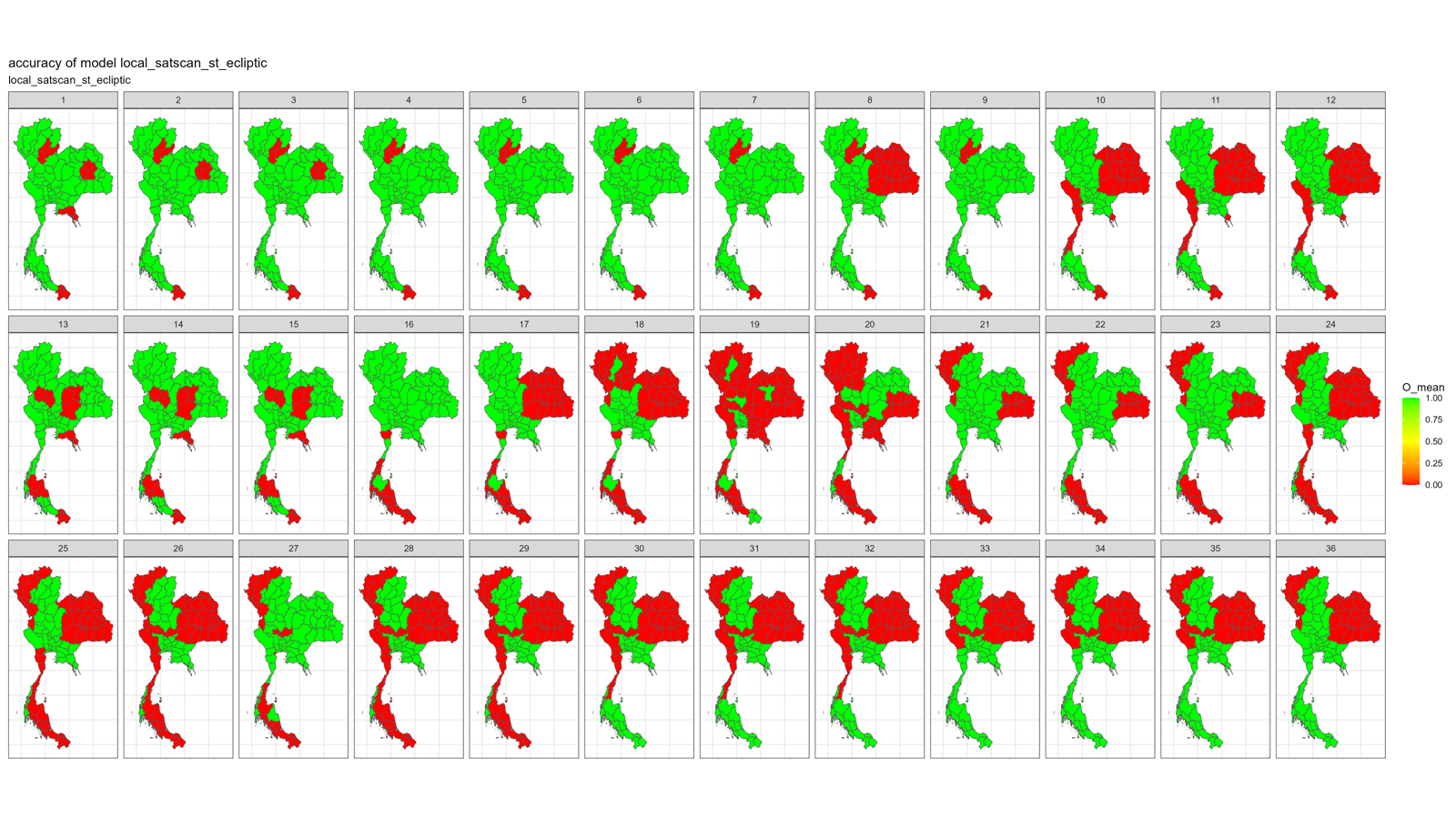


Accuracy maps of prospective ST SaTScan, elliptic windowing function, generated using RStudio version 2022.07.0+548 (available at https://posit.co/products/open-source/rstudio/).


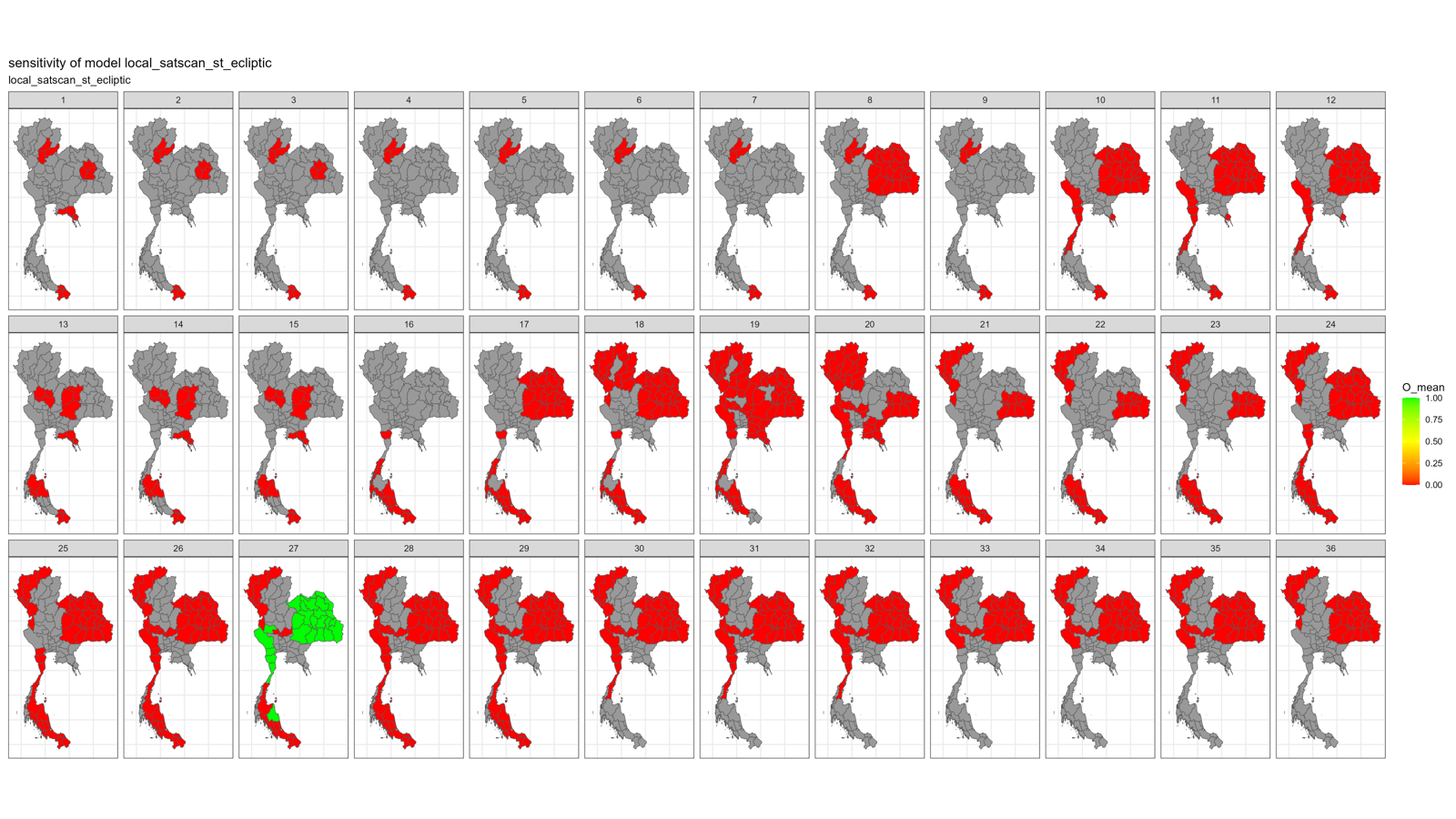


Sensitivity maps of prospective ST SaTScan, elliptic windowing function, generated using RStudio version 2022.07.0+548 (available at https://posit.co/products/open-source/rstudio/).


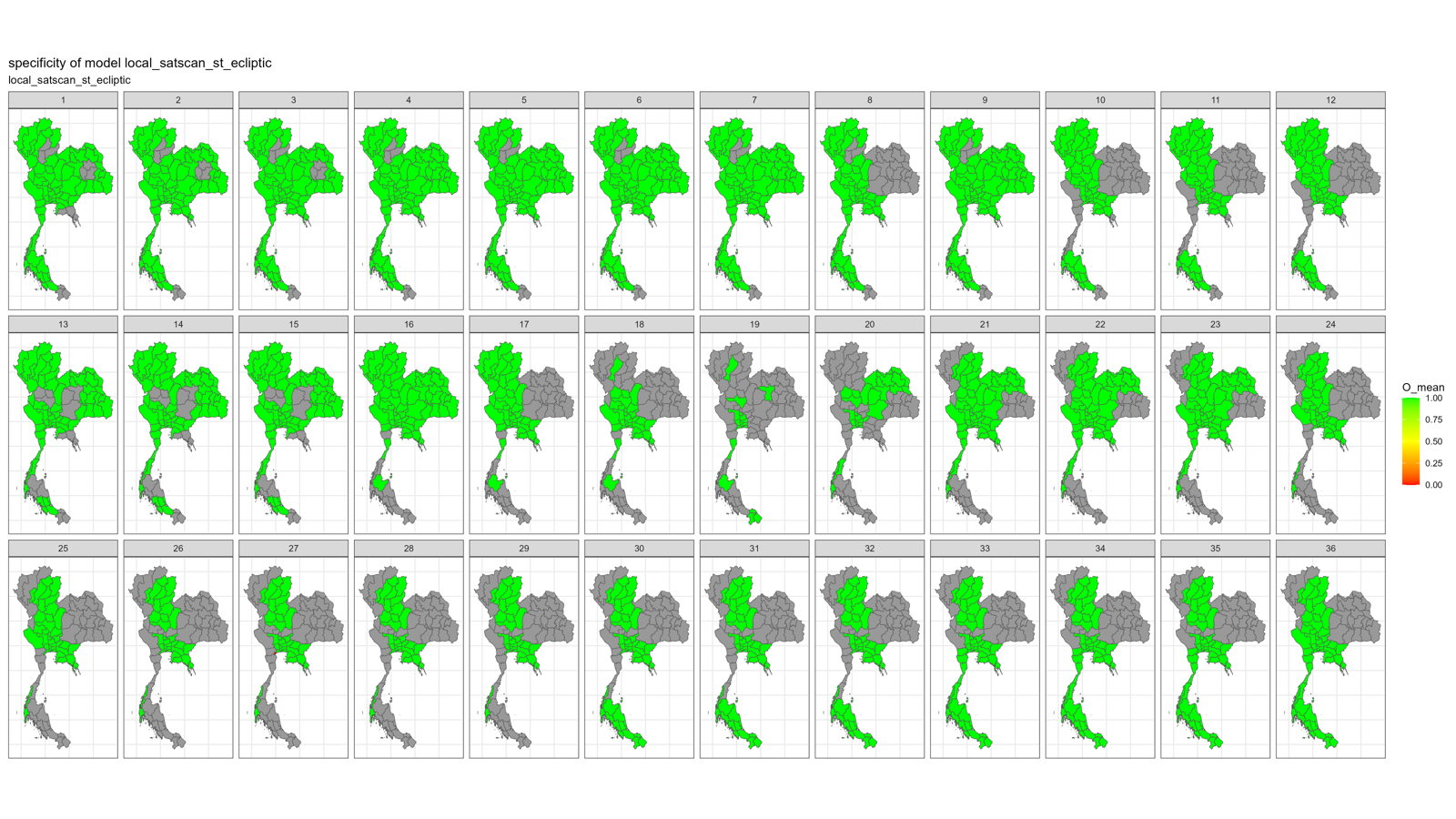


Specificity maps of prospective ST SaTScan, elliptic windowing function, generated using RStudio version 2022.07.0+548 (available at https://posit.co/products/open-source/rstudio/).


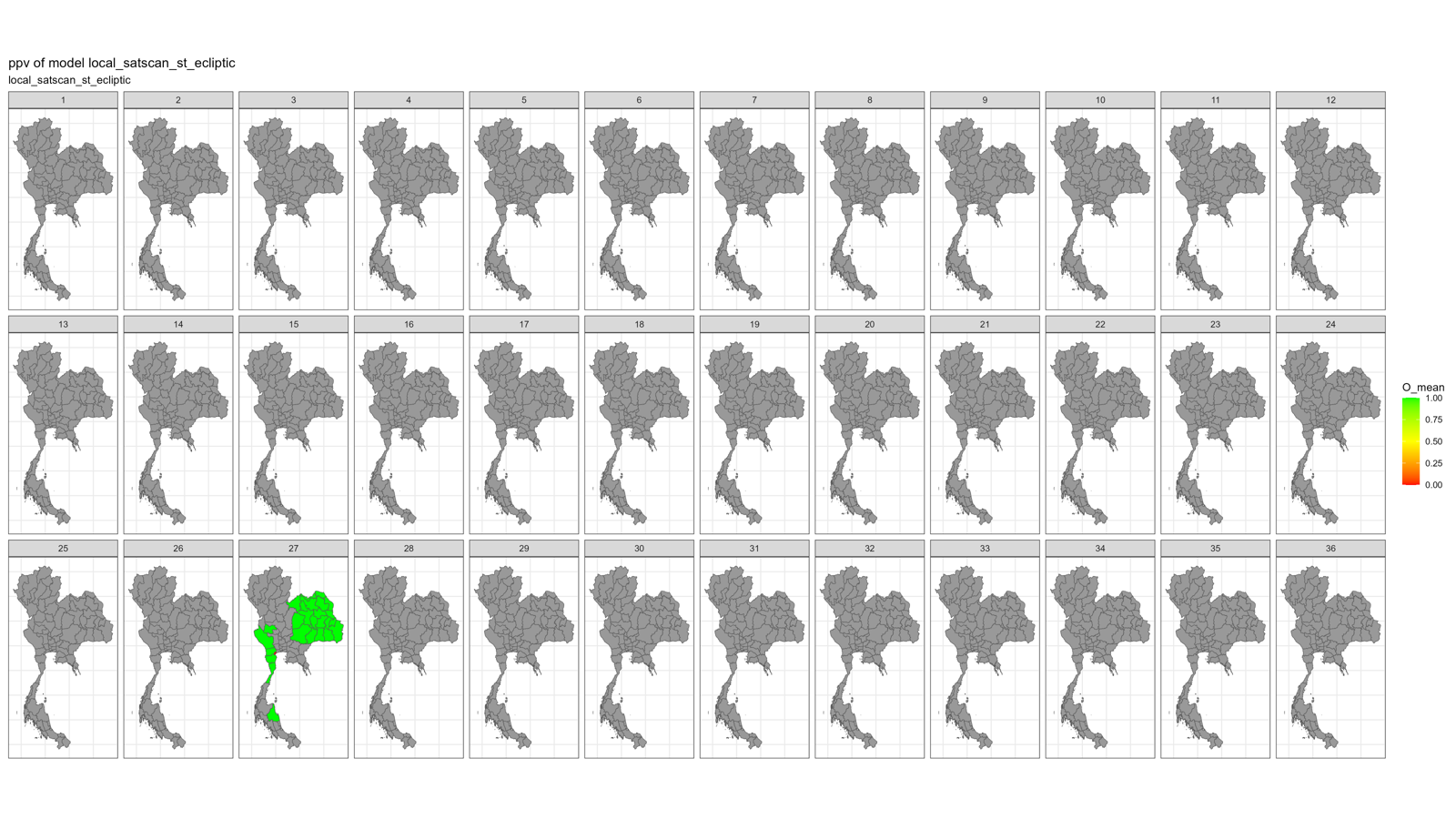


PPV maps of prospective ST SaTScan, elliptic windowing function, generated using RStudio version 2022.07.0+548 (available at https://posit.co/products/open-source/rstudio/).


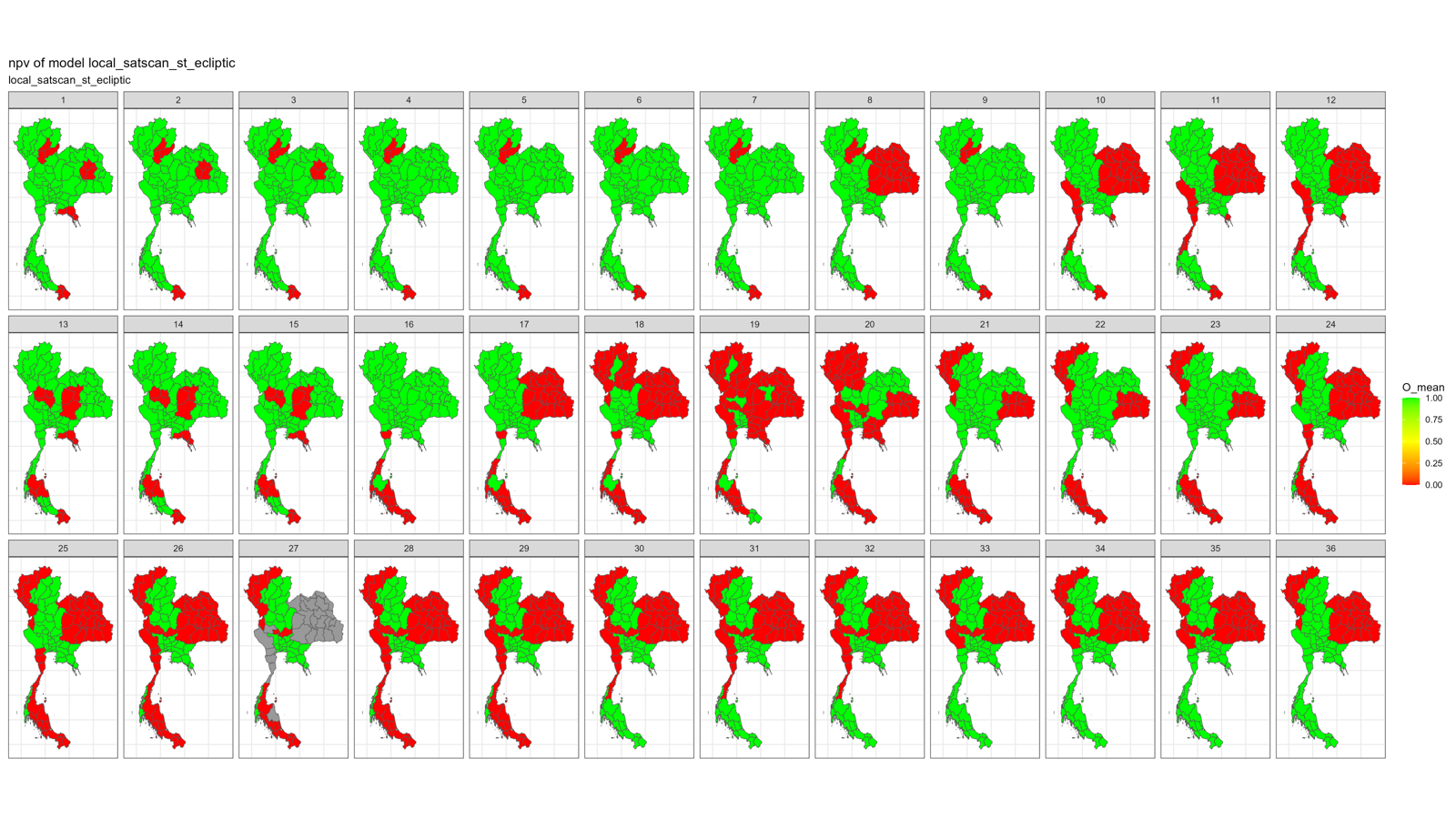


NPV maps of prospective ST SaTScan, elliptic windowing function, generated using RStudio version 2022.07.0+548 (available at https://posit.co/products/open-source/rstudio/).

*Getis ord Gi**


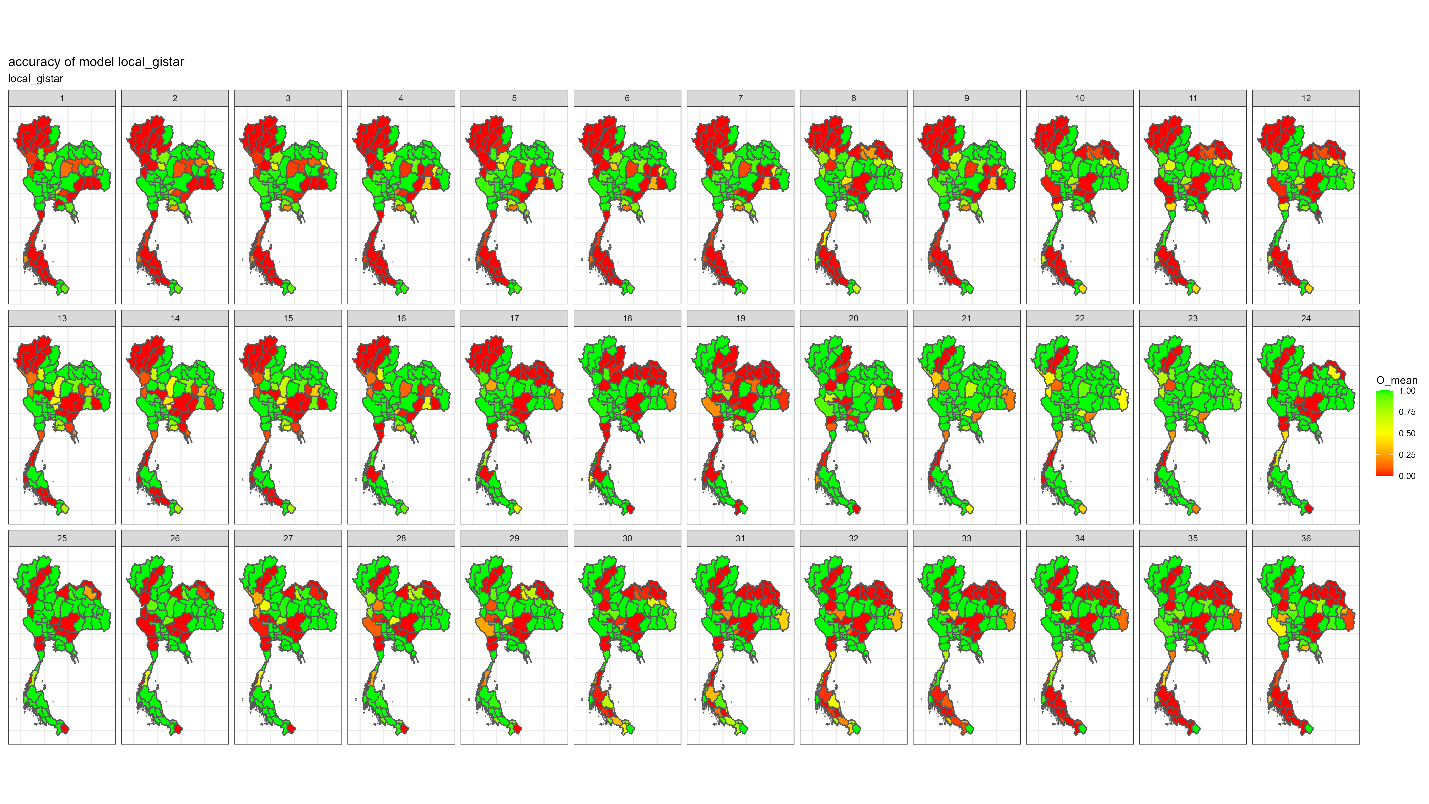


Accuracy maps of Getis ord Gi*, generated using RStudio version 2022.07.0+548 (available at https://posit.co/products/open-source/rstudio/).


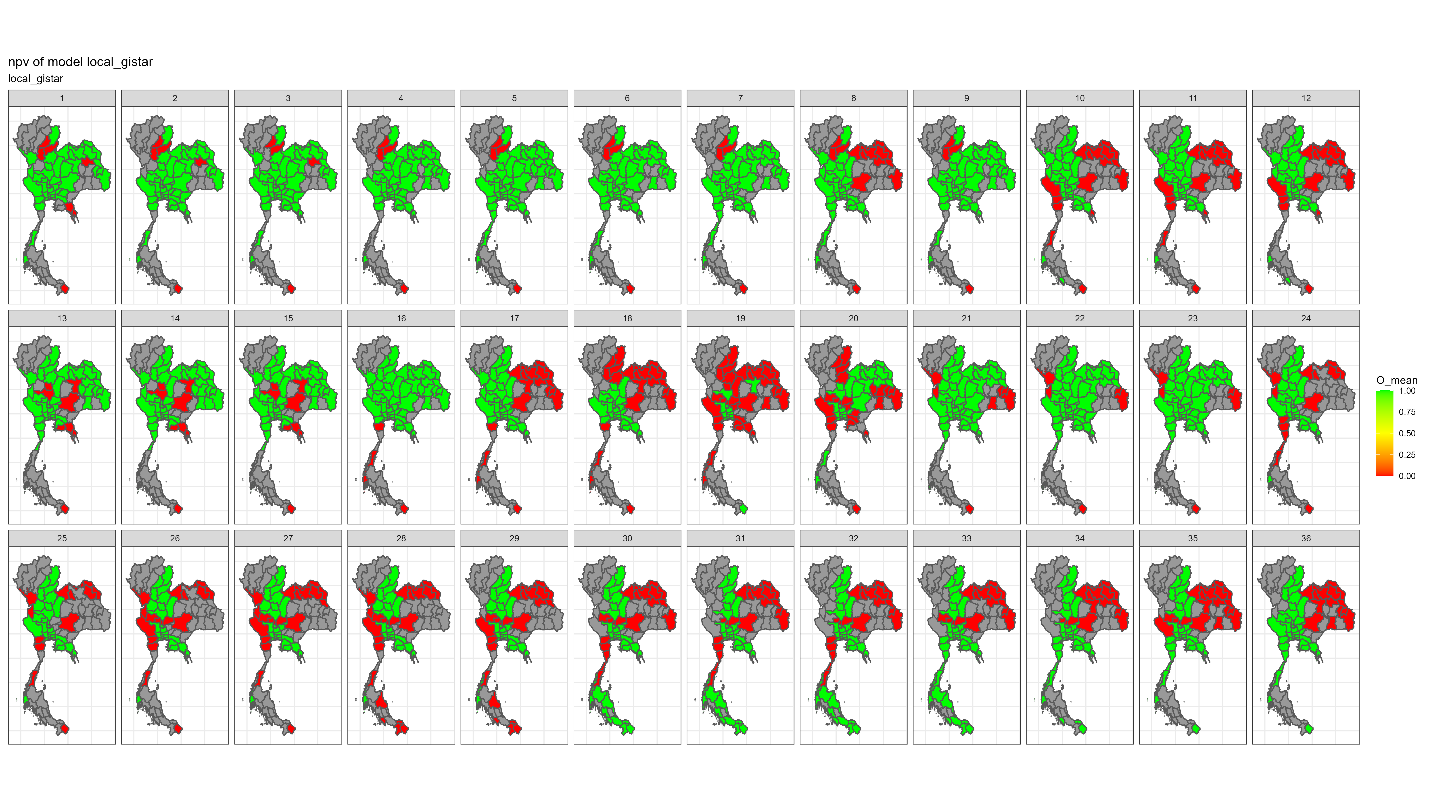


NPV maps of Getis ord Gi*, generated using RStudio version 2022.07.0+548 (available at https://posit.co/products/open-source/rstudio/).


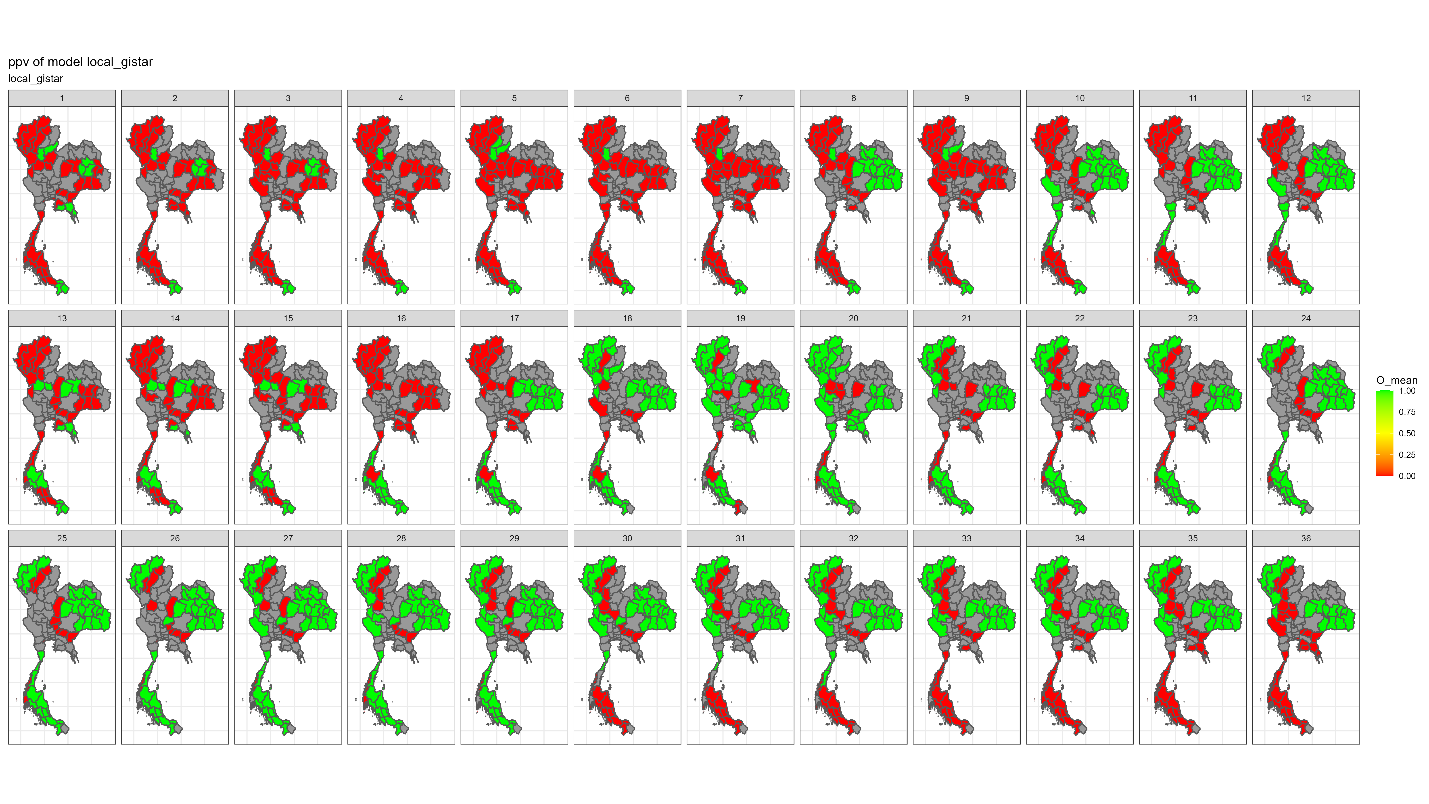


PPV maps of Getis ord Gi*, generated using RStudio version 2022.07.0+548 (available at https://posit.co/products/open-source/rstudio/).


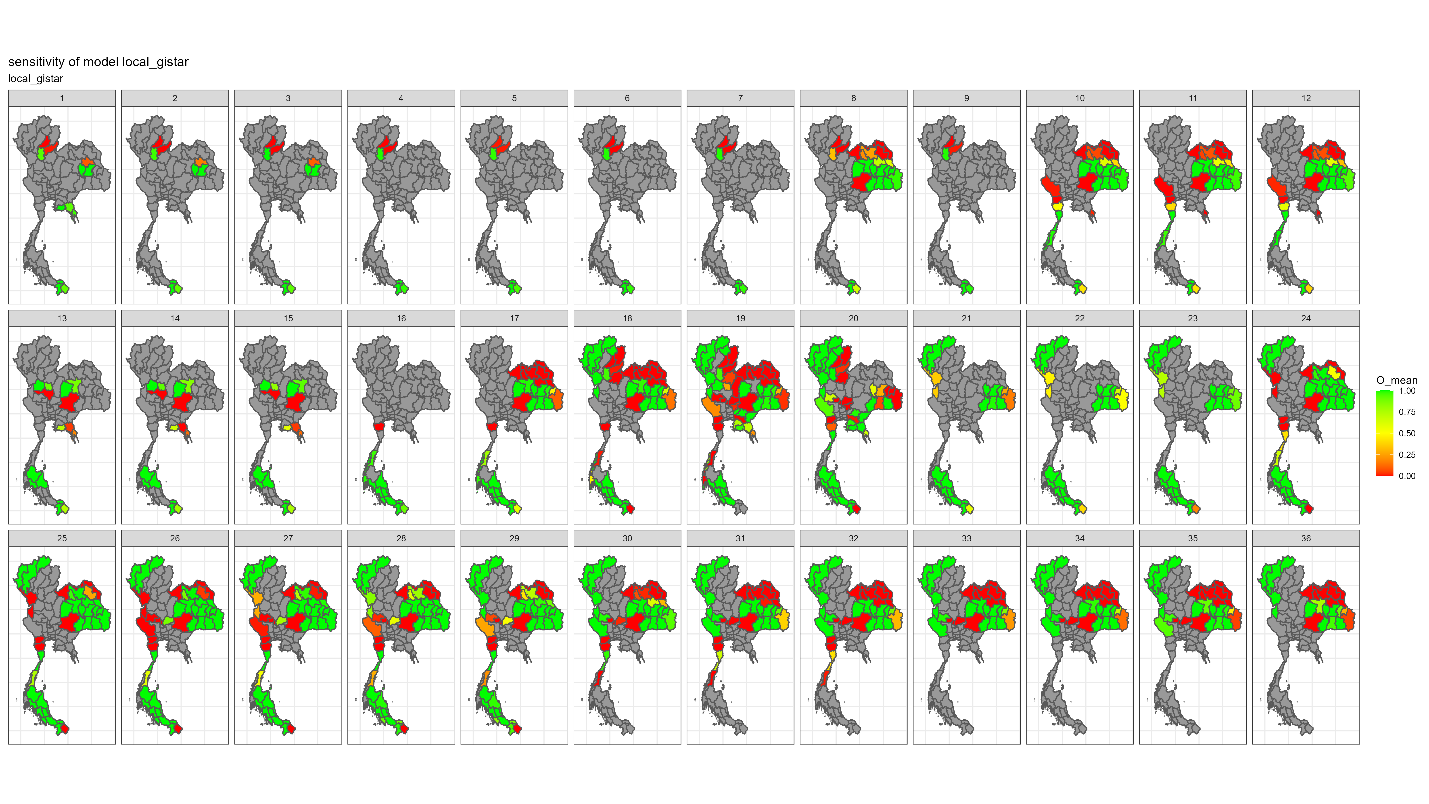


Sensitivity maps of Getis ord Gi*, generated using RStudio version 2022.07.0+548 (available at https://posit.co/products/open-source/rstudio/).


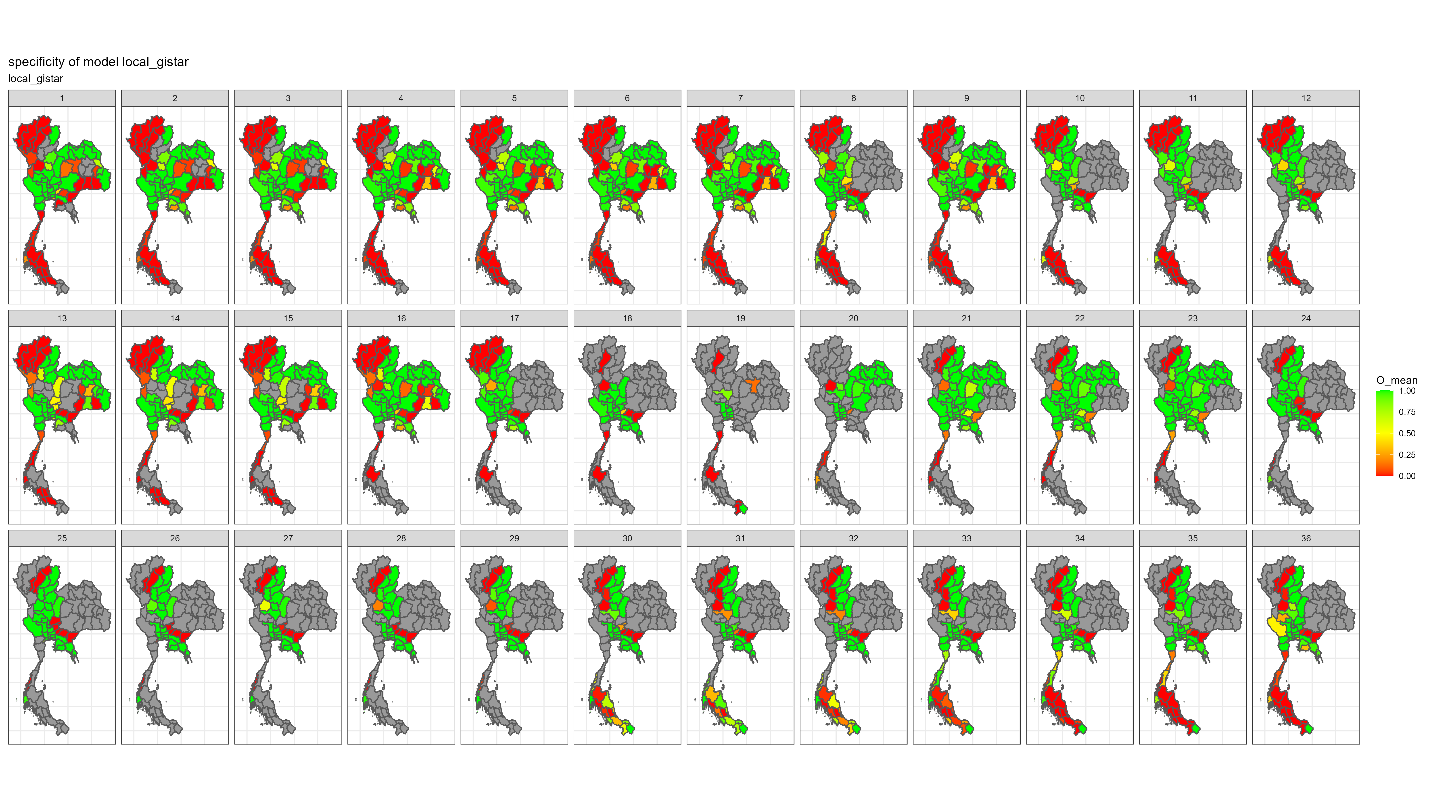


Specificity maps of Getis ord Gi*, generated using RStudio version 2022.07.0+548 (available at https://posit.co/products/open-source/rstudio/).

*Local Moran’s I*


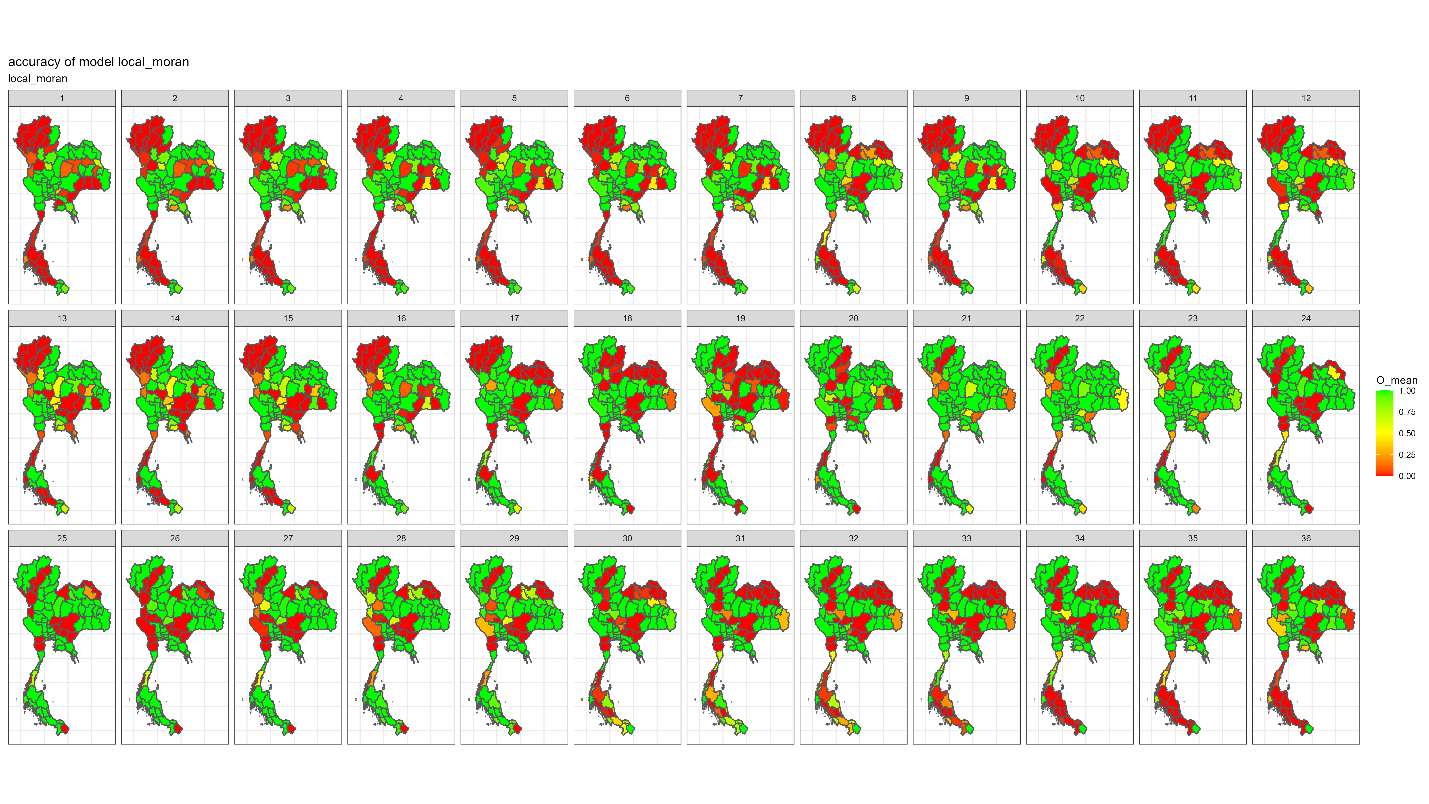


Accuracy maps of local Moran’s I, generated using RStudio version 2022.07.0+548 (available at https://posit.co/products/open-source/rstudio/).


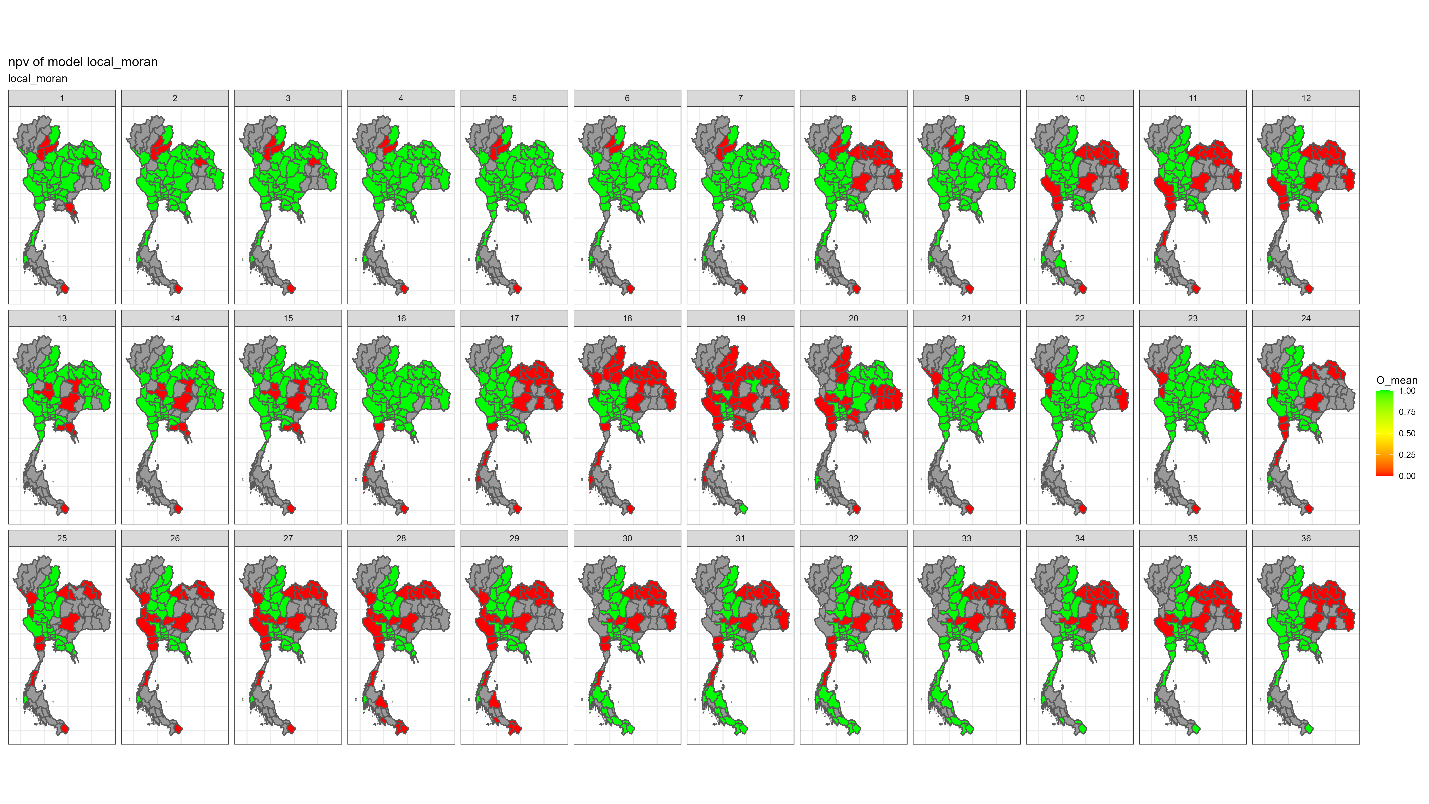


NPV maps of local Moran’s I, generated using RStudio version 2022.07.0+548 (available at https://posit.co/products/open-source/rstudio/).


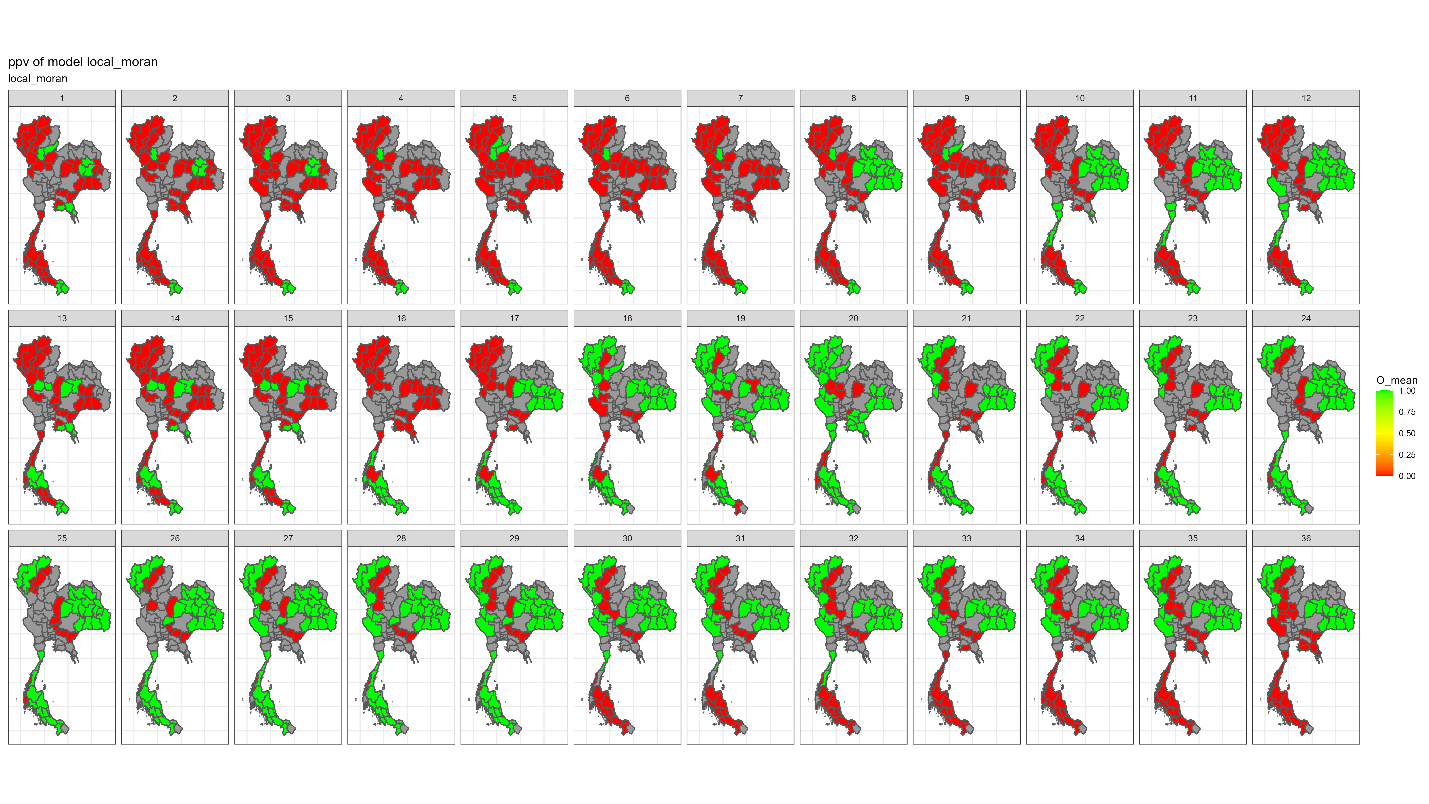


PPV maps of local Moran’s I, generated using RStudio version 2022.07.0+548 (available at https://posit.co/products/open-source/rstudio/).


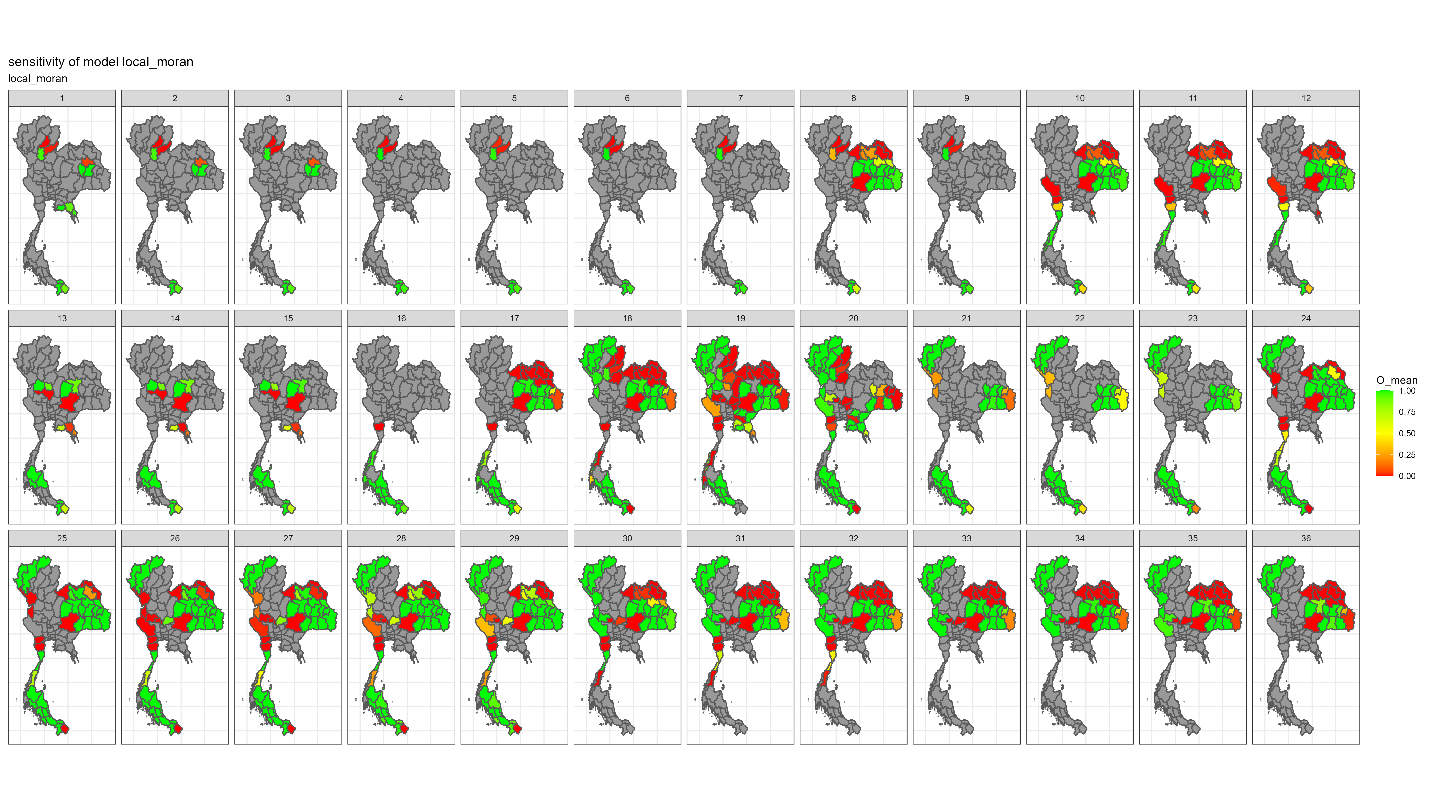


Sensitivity maps of local Moran’s I, generated using RStudio version 2022.07.0+548 (available at https://posit.co/products/open-source/rstudio/).


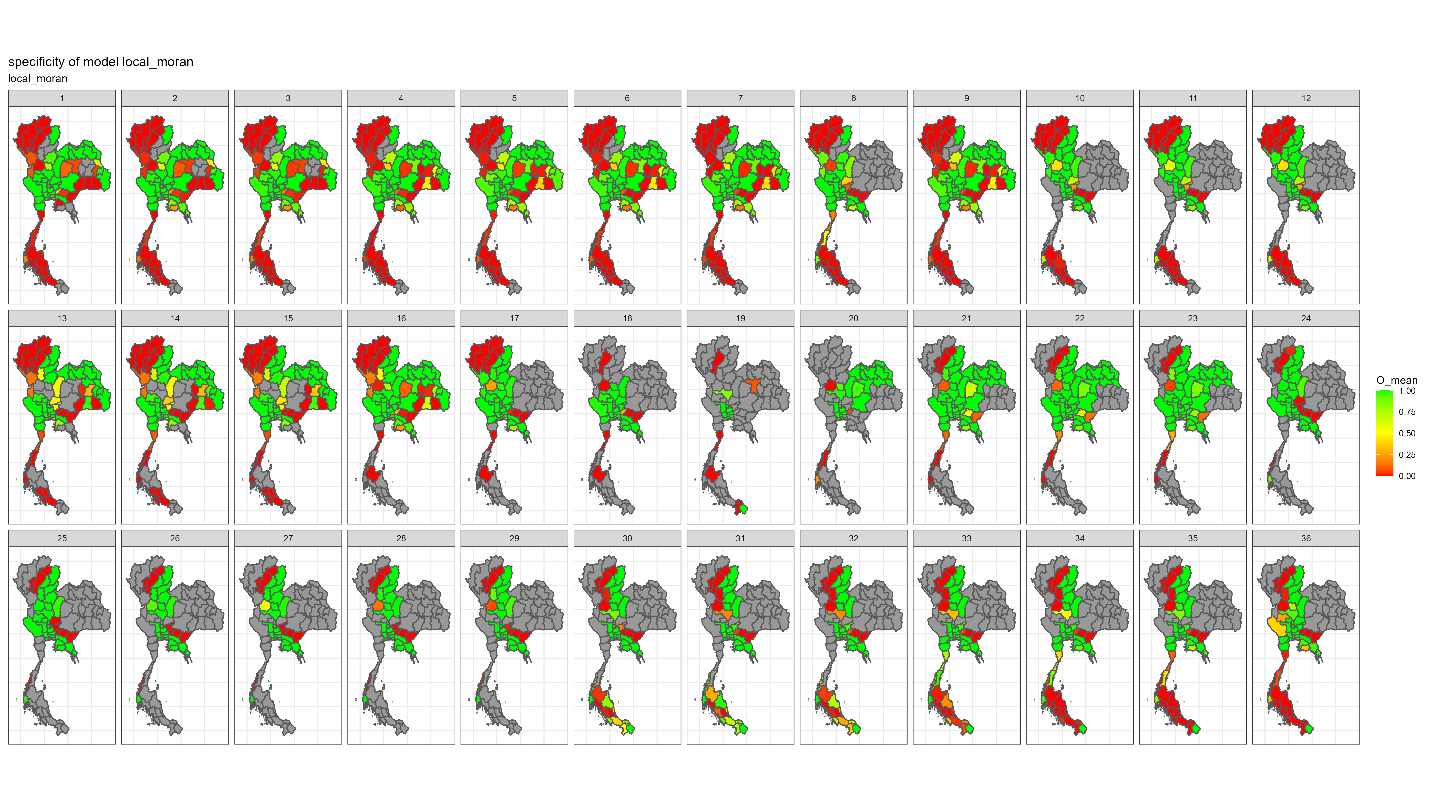


Specificity maps of local Moran’s I, generated using RStudio version 2022.07.0+548 (available at https://posit.co/products/open-source/rstudio/).

**Supplementary document S3: Results from case study of national dengue surveillance in Thailand**

Flexscan Circular maps from case study of national dengue surveillance in Thailand

, generated using RStudio version 2022.07.0+548 (available at https://posit.co/products/open-source/rstudio/).


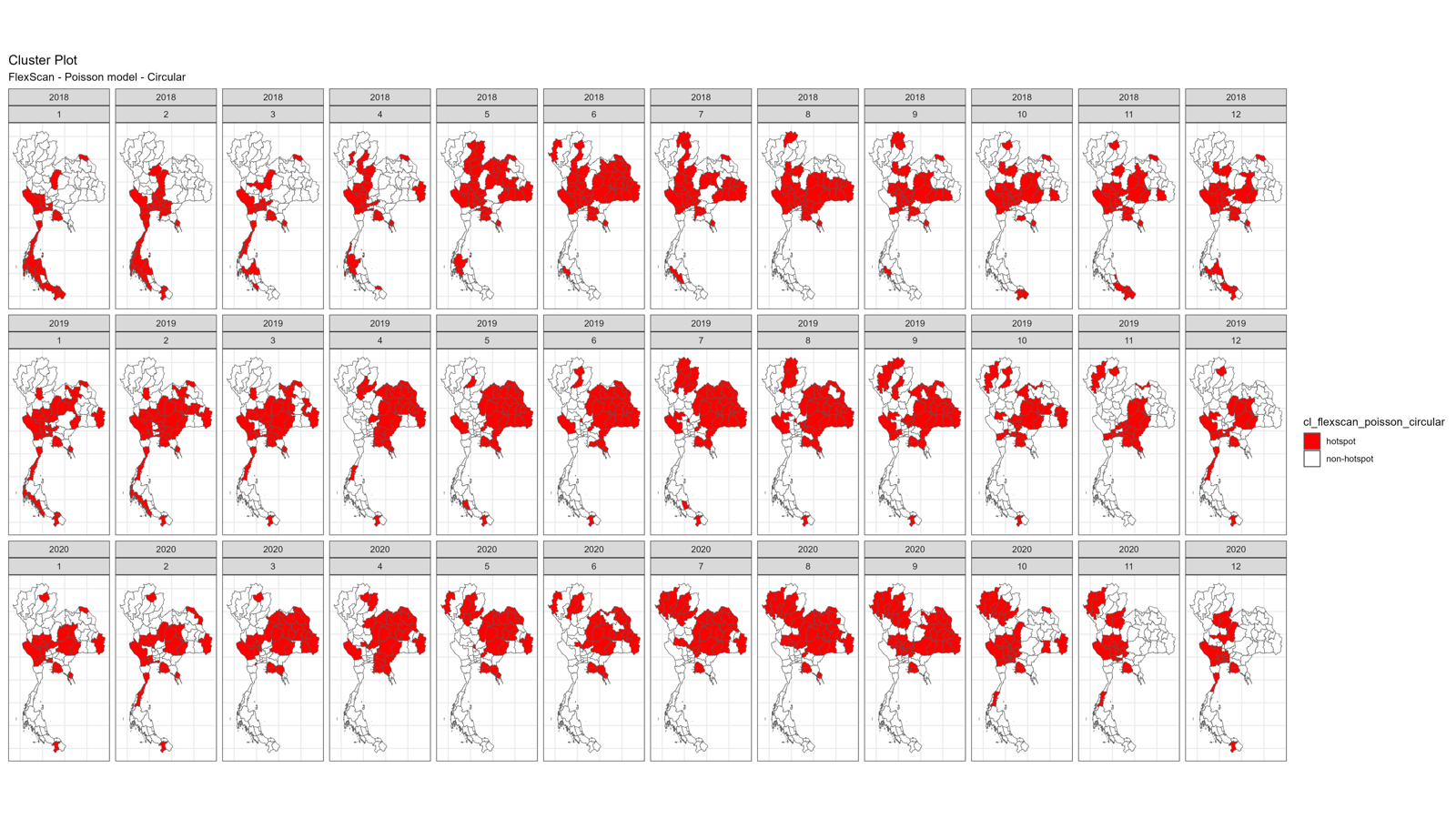


Flexscan Flexible maps from case study of national dengue surveillance in Thailand

, generated using RStudio version 2022.07.0+548 (available at https://posit.co/products/open-source/rstudio/).


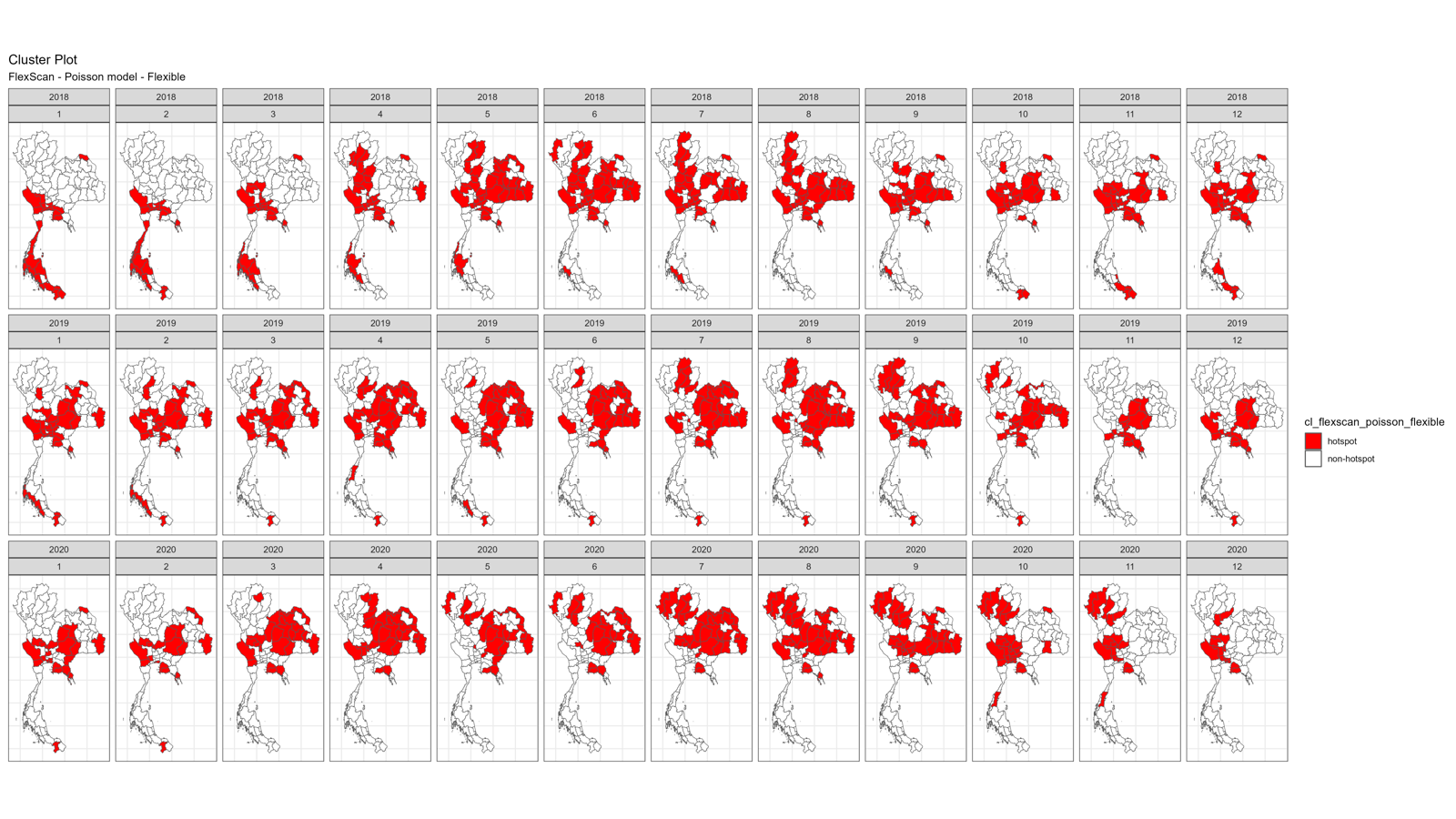


ST Satscan elliptic (retrospective) maps from case study of national dengue surveillance in Thailand, generated using RStudio version 2022.07.0+548 (available at https://posit.co/products/open-source/rstudio/).


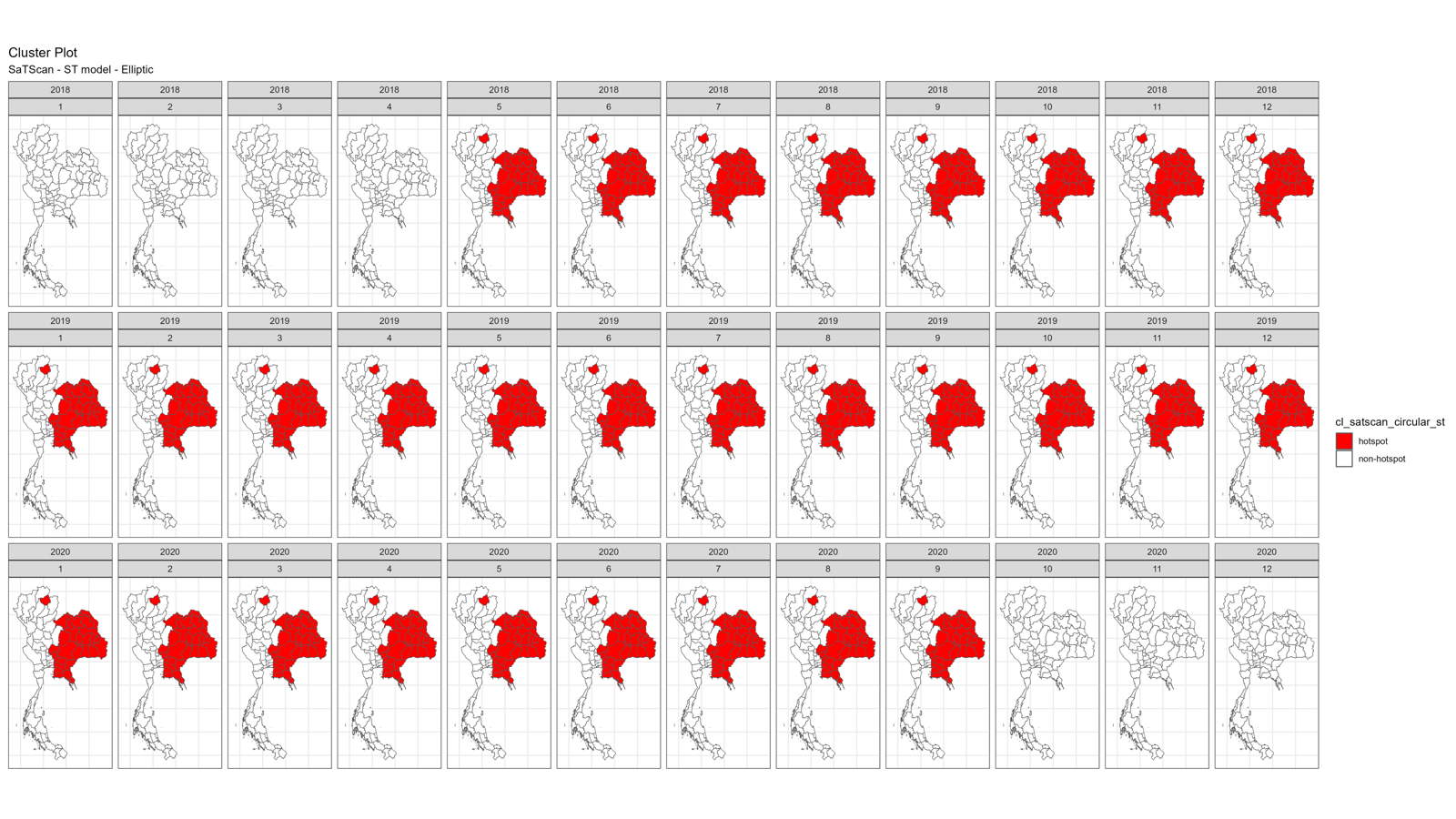


Satscan elliptic maps from case study of national dengue surveillance in Thailand

, generated using RStudio version 2022.07.0+548 (available at https://posit.co/products/open-source/rstudio/).


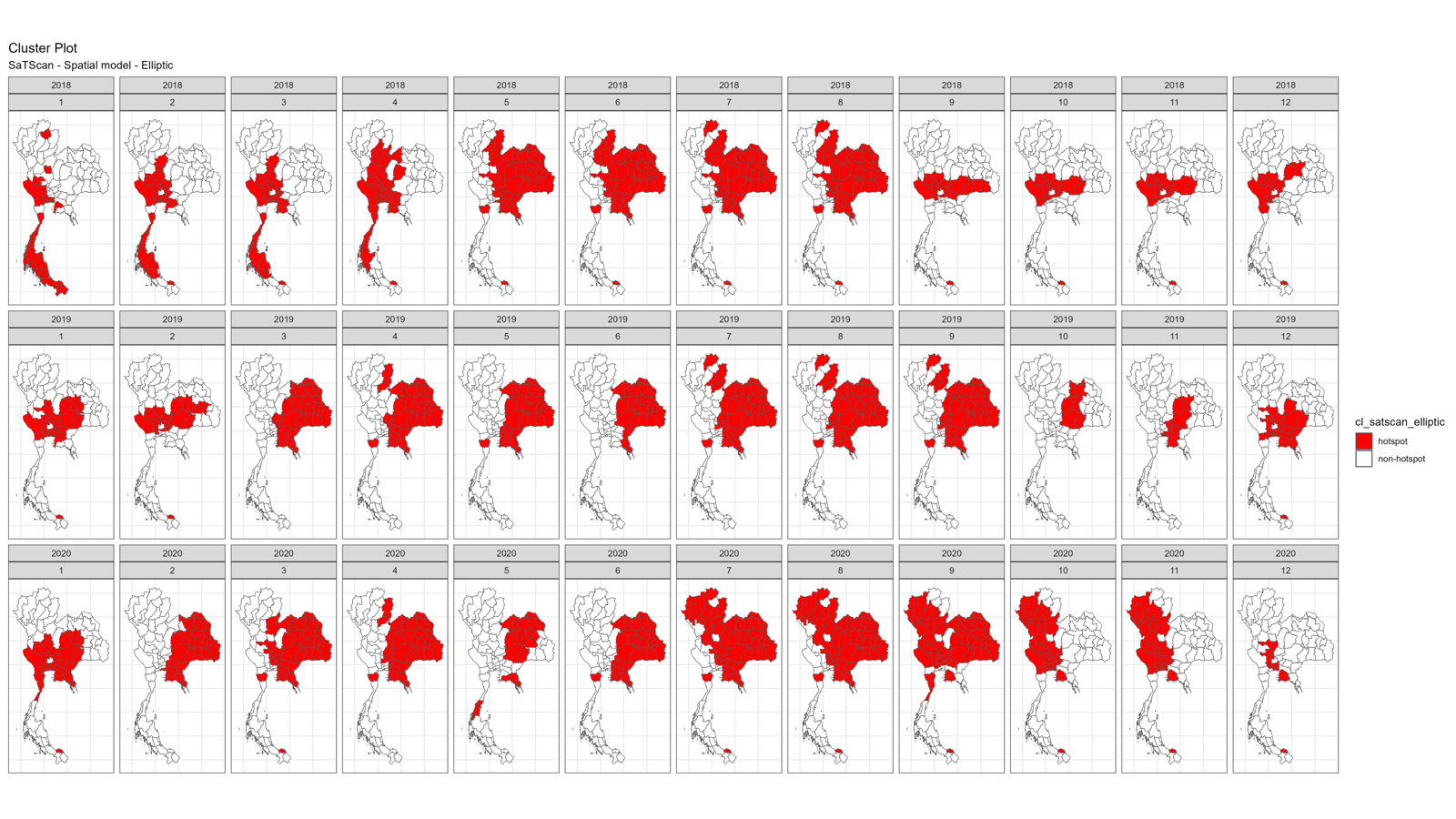


ST Satscan circular (prospective) maps from case study of national dengue surveillance in Thailand, generated using RStudio version 2022.07.0+548 (available at https://posit.co/products/open-source/rstudio/).


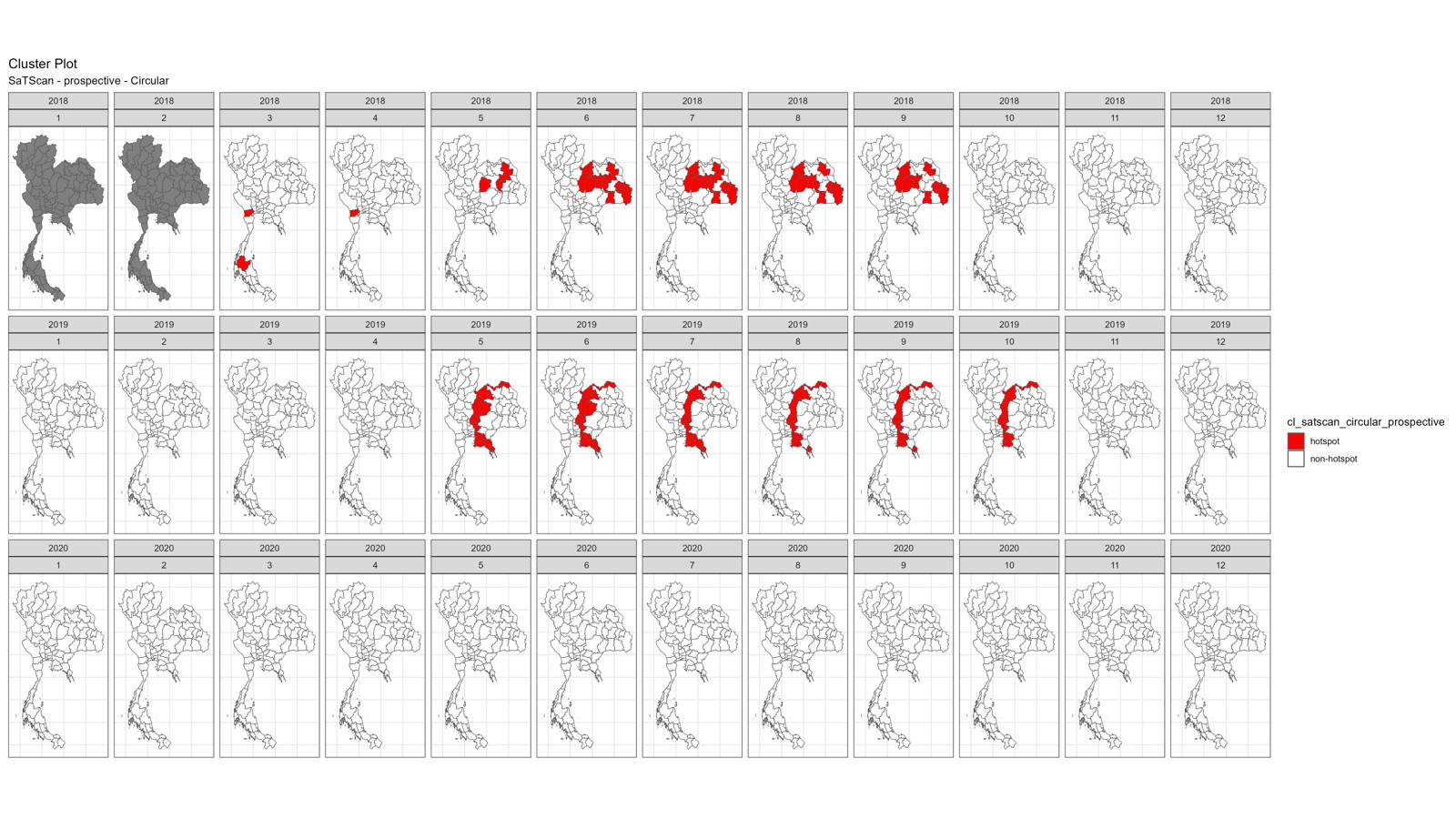


ST Satscan elliptic (prospective) maps from case study of national dengue surveillance in Thailand , generated using RStudio version 2022.07.0+548 (available at https://posit.co/products/open-source/rstudio/).


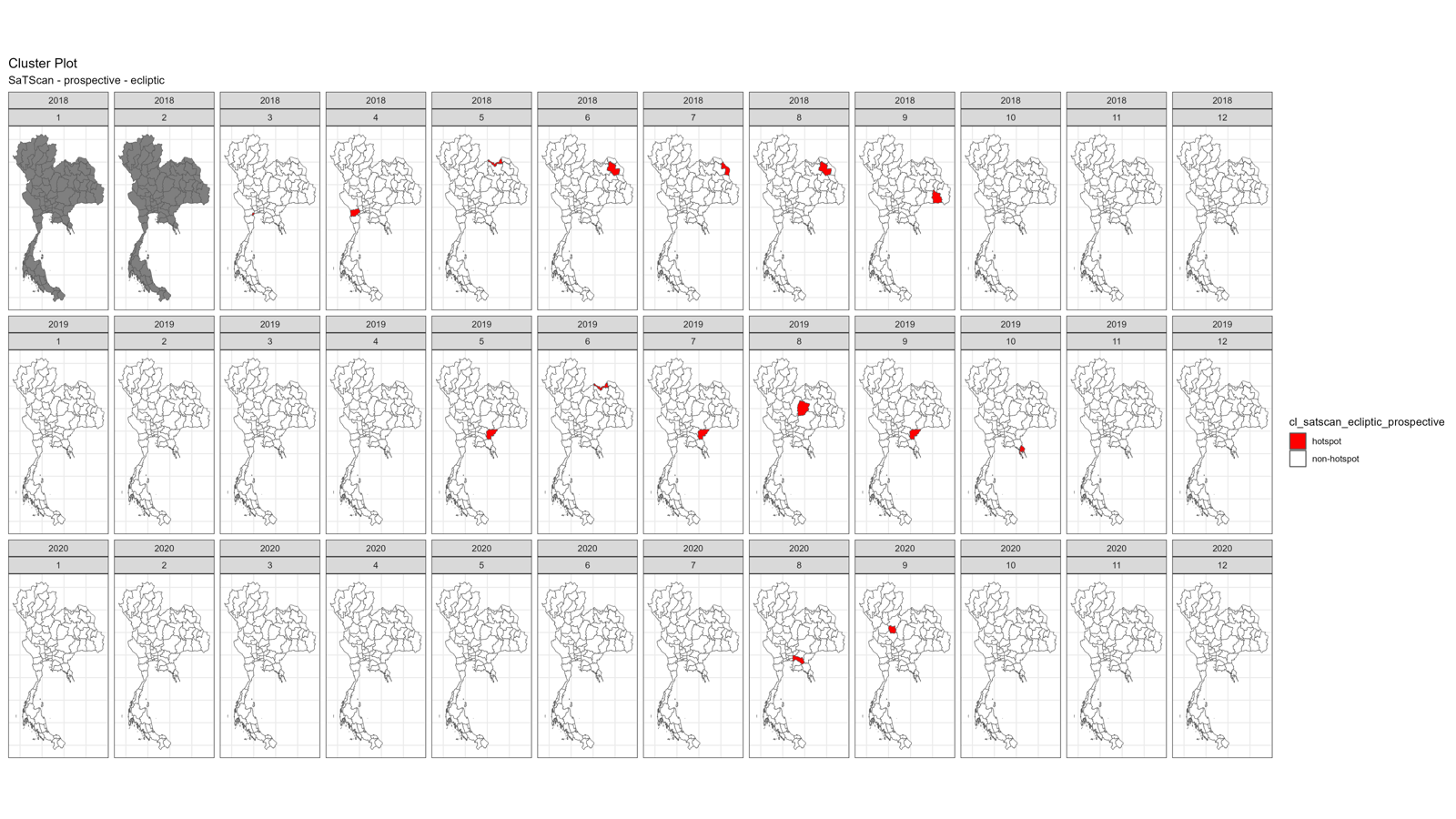


Getis Ord Gi* maps from case study of national dengue surveillance in Thailand

, generated using RStudio version 2022.07.0+548 (available at https://posit.co/products/open-source/rstudio/).


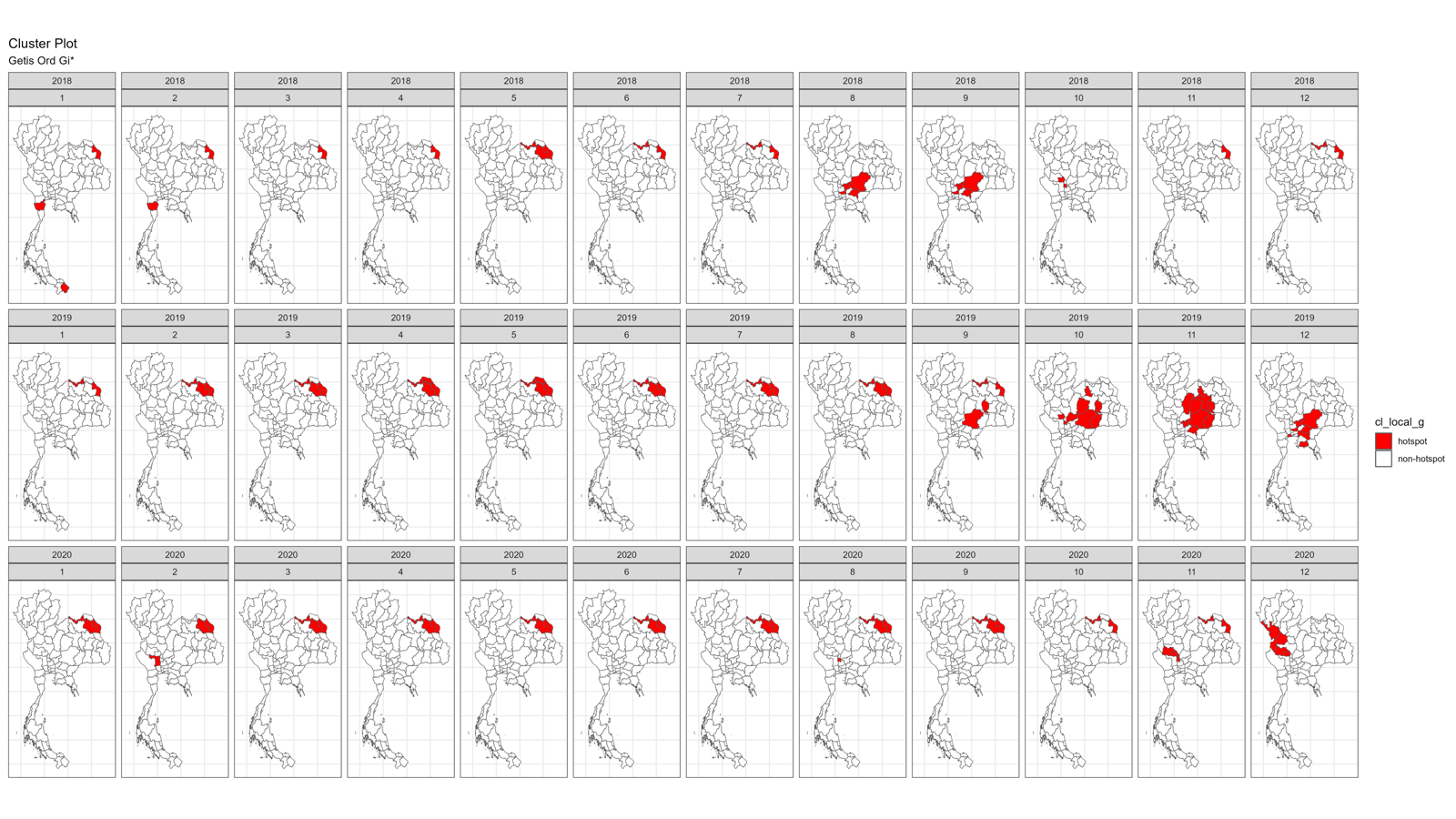


Local Moran’s I maps from case study of national dengue surveillance in Thailand

, generated using RStudio version 2022.07.0+548 (available at https://posit.co/products/open-source/rstudio/).


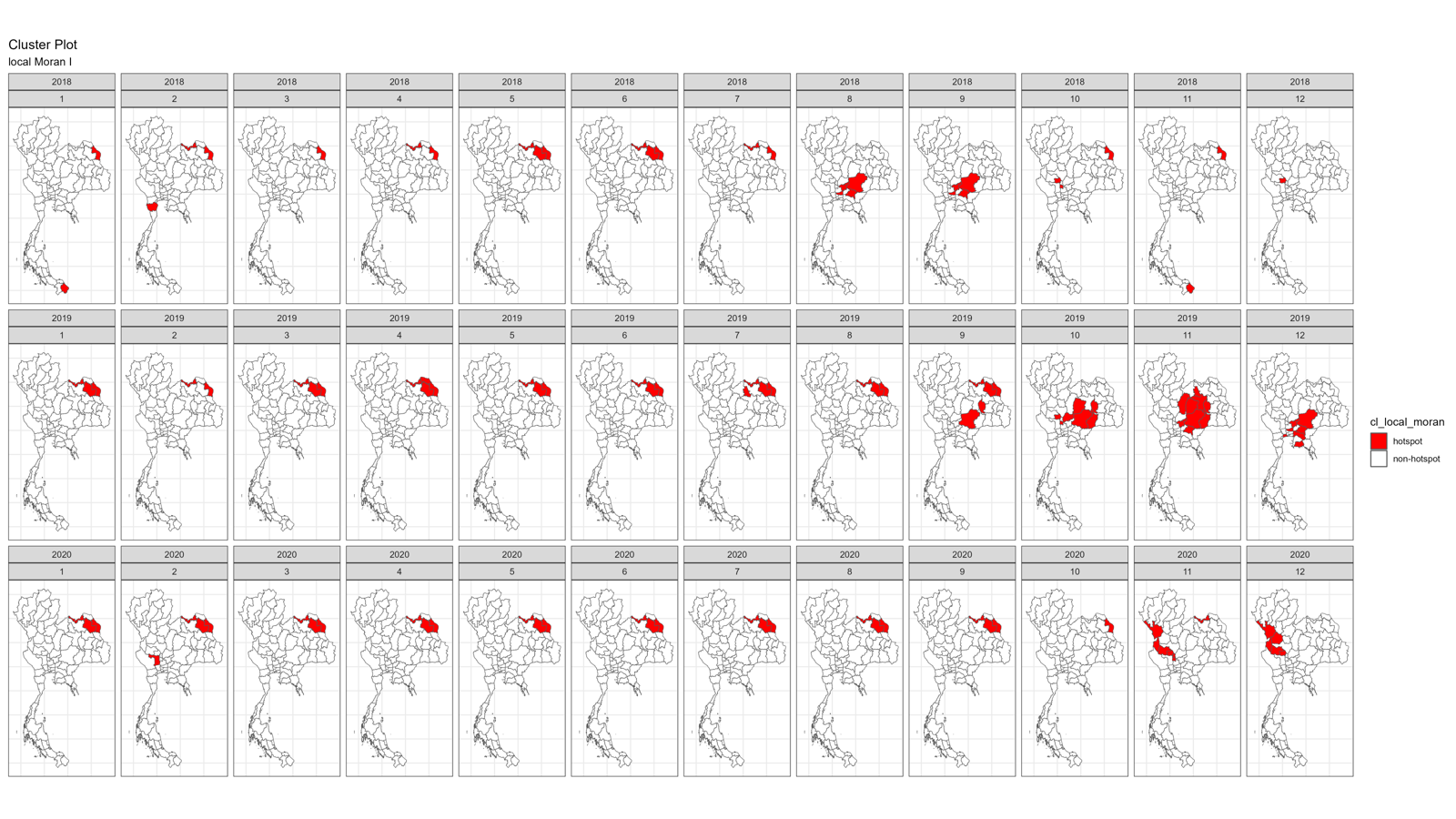


**References**

1. Anselin, L., *Local Indicators of Spatial Association—LISA.* Geographical Analysis, 1995. **27**(2): p. 93-115.

2. Ord, J.K. and A. Getis, *Local spatial autocorrelation statistics: distributional issues and an application.* Geographical analysis, 1995. **27**(4): p. 286-306.

3. Anselin, L. and A. Getis, *Spatial statistical analysis and geographic information systems.* The Annals of Regional Science, 1992. **26**(1): p. 19-33.

4. Kulldorff, M., *A spatial scan statistic.* Communications in Statistics-Theory and methods, 1997. **26**(6): p. 1481-1496.

5. Tango, T. and K. Takahashi, *A flexibly shaped spatial scan statistic for detecting clusters.* International Journal of Health Geographics, 2005. **4**(1): p. 11.

6. Dwass, M., *Modified Randomization Tests for Nonparametric Hypotheses.* The Annals of Mathematical Statistics, 1957. **28**(1): p. 181-187, 7.

7. Kulldorff, M., et al., *A space-time permutation scan statistic for disease outbreak detection.* PLoS Med, 2005. **2**(3): p. e59.

8. Kulldorff, M., *Prospective time periodic geographical disease surveillance using a scan statistic.* Journal of the Royal Statistical Society: Series A (Statistics in Society), 2001. **164**(1): p. 61-72.

9. Tango, T. and K. Takahashi, *A flexible spatial scan statistic with a restricted likelihood ratio for detecting disease clusters.* Statistics in Medicine, 2012. **31**(30): p. 4207-4218.

10. Blangiardo, M., et al., *Spatial and spatio-temporal models with R-INLA.* Spatial and spatio-temporal epidemiology, 2013. **7**: p. 39-55.

11. Lawson, A.B., et al., *Handbook of spatial epidemiology*. 2016: CRC press.

12. Aswi, A., et al., *Bayesian spatial and spatio-temporal approaches to modelling dengue fever: a systematic review.* Epidemiol Infect, 2018. **147**: p. e33.

13. Dunson, D.B., *Commentary: practical advantages of Bayesian analysis of epidemiologic data.* American journal of Epidemiology, 2001. **153**(12): p. 1222-1226.

14. Tango, T., *Disease Mapping: Visualization of Spatial Clustering*, in *Statistical Methods for Disease Clustering*. 2010, Springer New York: New York, NY. p. 33-47.

15. Besag, J., J. York, and A. Mollié, *Bayesian image restoration, with two applications in spatial statistics.* Annals of the institute of statistical mathematics, 1991. **43**(1): p. 1-20.

16. Knorr‐Held, L., *Bayesian modelling of inseparable space‐time variation in disease risk.* Statistics in medicine, 2000. **19**(17‐18): p. 2555-2567.

17. Blangiardo, M. and M. Cameletti, *Spatial and spatio-temporal Bayesian models with R-INLA*. 2015: John Wiley & Sons.

18. Rue, H., S. Martino, and N. Chopin, *Approximate Bayesian inference for latent Gaussian models by using integrated nested Laplace approximations.* Journal of the Royal Statistical Society: Series B (Statistical Methodology), 2009. **71**(2): p. 319-392.

19. Larner, A., *The 2x2 matrix: contingency, confusion and the metrics of binary classification*. 2021: Springer Nature.

20. Altman, D.G. and J.M. Bland, *Statistics Notes: Diagnostic tests 2: predictive values.* Bmj, 1994. **309**(6947): p. 102.
